# Holography Meets Theranostics: DFT/TDDFT Insights into Ru–NO@M_20_ (M = Au, Ag) and Ru–NO@Au_10_Ag_10_(Pc) Nanohybrids as Phase-Switchable Molecular Devices

**DOI:** 10.3390/ijms262412113

**Published:** 2025-12-16

**Authors:** Athanassios Tsipis, Niq Catevas

**Affiliations:** Laboratory of Inorganic Chemistry, Department of Chemistry, University of Ioannina, 45110 Ioannina, Greece; n.katevas@uoi.gr

**Keywords:** ruthenium–nitrosyl complexes, photoinduced linkage isomerism, metal–nanoparticle hybrids (Au_20_; Ag_20_), refractive-index modulation, holography-based theranostics, pyramidal gold/silver nanoparticles

## Abstract

Photo-induced bond linkage isomerization (BLI) in metal–nitrosyl compounds provides a molecular mechanism for controlling light-induced changes in refractive index and phase modulation. In this study, the ground and metastable states of a series of Ru–NO complexes and their Au_20_, Ag_20_, and mixed Au_10_Ag_10_ nanocluster hybrids were investigated by DFT and TDDFT calculations. The photochemical rearrangement between the linear, side-on, and O-bound forms of Ru–NO was examined together with their electronic transitions, oscillator strengths, and characteristic vibrational shifts. From these data, parameters describing radiative efficiency, non-radiative coupling, and metastable-state stability were derived to identify compounds with favorable properties for holography and photonic applications. Particular attention was given to the [(Salen)Ru(NO)(HS)@Au_20_] complex, which shows a strong red-to-NIR response and balanced stability among its linkage isomers. Frequency-dependent polarizabilities α(ω) were calculated for its ground and metastable states and compared with those of the classical holographic material [Fe(CN)_5_NO]^2−^ (nitroprusside). The refractive-index changes derived from α(ω) reveal that the Au_20_–salen hybrid produces a much larger and more strongly wavelength-dependent Δ*n*(*λ*) than nitroprusside. At 635 nm, the modulation reaches approximately 0.06 for the hybrid, compared with 0.02 for nitroprusside. This enhancement reflects the cooperative effect of the Ru–NO chromophore and the Au_20_ nanocluster, which amplifies both polarizability and optical dispersion. The results demonstrate that coupling molecular photo-linkage isomerism with nanoplasmonic environments can significantly improve the performance of molecular systems for holography and optical-phase applications.

## 1. Introduction

It has been more than five decades since the fortuitous discovery of the anticancer activity of cisplatin by Rosenberg [1]. This seminal discovery triggered an intense study on the potential anticancer activity of Platinum as well as of other transition metal complexes [2,3]. The result of these studies was the second-generation Pt anticancer agents, with the most notable being Carboplatin and Nedaplatin, and later on the third-generation Pt anticancer complexes, which include Oxaliplatin, Heptaplatin, and Lobaplatin, amongst others [4,5,6]. These drugs were developed in order to address various issues, such as severe systemic toxicities, kidney damage, overcoming drug resistance, and better toxicity profiles to be better tolerated by patients. Various other transition metal complexes have also been studied as anticancer agents [7], with notable examples being those of Ru [8,9], Au [10,11], as well as of Ir, Re, and Os [12]. Nevertheless, one major challenge in using metallodrugs for cancer treatment is the ability to monitor their biological action inside the body. Monitoring metallodrugs offers a variety of advantages, such as the detection of their accumulation not only on damaged tissues but also on healthy ones, being a major source of the toxic side effects, which are related to the dose and its suitable adjustment [13]. Currently, various methods are used to monitor the antitumor activity of drugs, such as Magnetic Resonance Imaging (MRI), Computed Tomography (CT), and Positron Emission Tomography (PET) [14,15,16,17], with various levels of success. On the other hand, a powerful method to monitor anticancer drugs is luminescence-based imaging [18]. Luminescence offers the advantage of combining diagnosis and therapy using single molecules. Integrating diagnostic imaging and therapy into a single platform offers the ability to realize the so-called theranostics. The term arises from the combination of ‘therapy’ and ‘diagnosis’ [19], and theranostics is a rapidly growing field within the framework of cancer treatment. Transition metal complexes, and particularly those of Ir(III), Ru(II), and Re(I), have been used as theranostics due to their favorable photophysical properties [18]. Thus, many Ir complexes have been studied for bioimaging due to their stability and emission properties [20,21]. Their characteristic is that they have a tendency to accumulate in mitochondria, inducing cell death [18]. On the other hand, Ru complexes have attracted significant attention as both anticancer agents [22], as well as for bioimaging [23], due to their selectivity towards cancer cells, optimal ligand exchange kinetics, and biologically accessed, multiple oxidation states. Recent work has also highlighted ruthenium nitrosyl complexes as photochemical NO donors, molecular photoswitches, and emerging theranostic platforms [24,25,26].

An emerging class of materials is the Transition Metal Complex—Metal Nanoparticles (TMC-MNPs) [27]. These are hybrid materials consisting of a TM complex attached to the surface of a nanoparticle via one of its ligands, which acts as a bridge. The TMC-MNPs hybrid materials are very promising for a plethora of applications, amongst others, as theranostics, since they combine the plasmonic behavior of the metal nanoparticles, already exhibiting fascinating properties, such as strong optical response, with the chemical/photochemical functionality of transition metal complexes. Gold, silver, and bimetallic nanoclusters are now recognized as atomically precise platforms with tunable charge–transfer and optical properties [28,29], and recent computational studies indicate that nanocluster adsorption can modulate molecular electronic and nonlinear optical responses [30]. Thus, the combination of the TM complex with the metal NP in the TMC-MNPs systems enables synergistic effects resulting in enhanced light absorption and emission, energy/charge transfer at the interface, and regulated photoactivation [31]. Due to their good solubility and low toxicity [32], the TMC/MNP hybrid materials are very attractive for use in biological applications, such as imaging, as well as photodynamic therapy (PDT) [33,34].

Transition metal complexes are also promising for use as functional materials for holography [35]. Along these lines, the photoinduced bond-linkage isomerization (BLI) observed in various TM complexes is exploited in order to produce holograms [36,37]. Amongst the first successful attempts was that by Imlau et al. [38], who demonstrated that upon irradiating sodium nitroprusside, [Fe(CN)_5_NO]^2−^ is produced and a full optical write–read–erasure cycle could be achieved, being fundamental for materials-based holography. The photofunctionality of these TM complexes, stems from their ability to undergo NO bond linkage upon irradiation which excites them from their ground state (GS), with NO η^1^-bound via its N donor atom to metal (η^1^-NO), to an excited state (GS*) from where two metastable species are formed namely the MSI with NO η^1^-bound via its O donor atom to metal (η^1^-ON) as well as the MSII where NO is η^2^-bound via both N and O donor atoms simultaneously (η^2^-NO) [35]. Another notable example of use of a TM complex for holography is the [Ru(bpy)_2_(SO)]^+^ Ru sulfoxide complex, where its photofunctionality arises from an SO, rather than an NO, bond ligand isomerization [39,40].

Motivated by the fascinating properties of the TMC-MNPs hybrid materials, we thought it would be interesting to investigate their potential use in optical imaging, employing a novel approach, not common in molecular theranostics. This new approach is a holography-based detection, which, instead of measuring luminescence, would rely on monitoring changes in the phase or refractive index of light passing through materials. Hybrid molecular–plasmonic architectures have recently been identified as promising photonic platforms where molecular switching is coupled with locally enhanced optical fields [41,42]. Simultaneously, advances in nanoscale holography and multidimensional optical encoding demonstrate how phase-based readout mechanisms can be exploited in adaptive information storage materials [43,44,45]. These developments parallel the growth of plasmonic nanomaterials in theranostic imaging and targeted treatment [42,46]. Along these lines, TMC-MNPs hybrid materials are very attractive for such a purpose since their structure, comprising the coupling of a metal nanocluster to a photoactive TM unit, is expected to generate strong, holographically visible signals. This rudimentary theoretical study could form the basis for initiating studies on a whole new class of theranostics, based on holography instead of the classic luminescence theranostics. Accordingly, we set out to study, by means of DFT/TDDFT calculations, a series of TMC-MNPs hybrid materials with the general formula [(L)Ru(NO)(HS)@M_x_M′_20−x_] (L = salen, bpb, porph, or phthalocyanine, M, M′ = Au, Ag, x = 0, 10 or 20,) where an octahedral Ru(II) nitrosyl is bound to a M_20_ nanocluster via its axial thiolate ligand. For the mixed-metal systems (Au_10_Ag_10_), only the phthalocyanine (L = Pc) derivatives were included in this study. These complexes could be thought of as models of TMC-MNPs hybrid materials where the M_20_ nanocluster is used to simulate the bulk nanoparticle surface, or they could even be synthesized as such, since, for example, atomically precise Au_20_ NPs have already been isolated and characterized, demonstrating the feasibility of defined small-size NPs [34,35,47,48]. In addition, [(L)Ru(NO)(HS)@M_x_M′_20−x_], bearing the Ru-NO unit, could also act as photoNORMs (photoactivated NO-releasing molecules) and be used in photodynamic therapy (PDT), where, upon irradiation, they could release NO, acting as photoNORMs (photo NO releasing molecules). If this NO-releasing process alters the optical properties of these hybrid materials, then holography might detect the switching between states. The primary objectives of this study are: (a) to investigate the ground-state (GS) electronic structure of the hybrid complexes of [(L)Ru(NO)(HS)@M_20_]; (b) to identify and characterize the metastable linkage isomers MSI and MSII; (c) to compute and analyze the absorption spectra of the GS, as well as of the MSI and MSII states; and (d) to evaluate the influence of the metallic nanoparticle (NP) nature on their photophysical behavior. To the best of our knowledge, this work represents the first systematic exploration of a fundamentally new and unconventional approach that offers an alternative mode of theranostic operation compared with the fluorescence-based detection typically used in drug-delivery systems. The present results suggest that transition-metal-complex/metal-nanoparticle (TMC–MNP) hybrids could form the basis of a next-generation class of “holography theranostics”, where optical information is encoded and read through phase-based rather than luminescence-based mechanisms. This concept paves the way toward a new direction in molecular imaging and drug monitoring that relies on controllable refractive-index modulation instead of emissive contrast.

## 2. Results and Discussion

### 2.1. Structural Parameters

#### 2.1.1. Benchmarking the Computational Protocol

**Figure 1:**
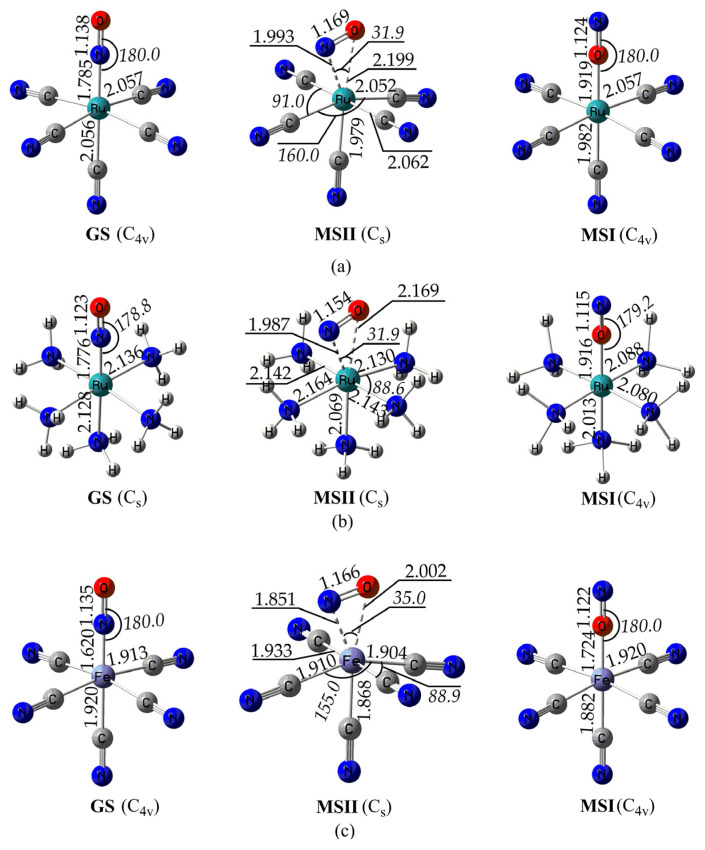
Geometries of the ground (GS), metastable I (MSI), and metastable II (MSII) states of (**a**) [(CN)_5_Ru(NO)]^2−^, (**b**) [(NH_3_)_5_Ru(NO)]^3+^, and (**c**) [(CN)_5_Fe(NO)]^2−^ calculated at the PBE0/LANL2DZ(Ru)/6-31G(d,p)/PCM(water) level of theory.

The geometries of all species studied were fully optimized, employing the PBE0/LANL2DZ(M)U6-31G(d,p)(E)/PCM(water) computational protocol (see Computational Details section for details). Before applying this DFT computational methodology to the [(L)Ru(NO)(HS)@M_x_M′_20−x_] hybrid complexes of interest, we set out to benchmark it on a set of three model complexes. These complexes are the [(CN)_5_Ru(NO)]^2−^, the [(NH_3_)_5_Ru(NO)]^3+^, and the [(CN)_5_Fe(NO)]^2−^, for which there are experimental as well as previous theoretical data available. Figure 1 depicts the geometries of these model complexes calculated with our computational protocol, namely, the PBE0/LANL2DZ(M)U6-31G(d,p)(E)/PCM(water). In addition, selected structural parameters are given in Table 1 and Table S1 (see Supplementary Materials) for direct comparison with the respective X-ray experimental data as well as with data calculated with other DFT methods.

Inspection of Figure 1 and Table 1 shows that the ground-state (GS) geometry of the [(CN)_5_Ru(NO)]^2−^ model complex, calculated with the PBE0/LANL2DZ(M)U6-31G(d,p)(E)/PCM(water), is in very good agreement with the X-ray experimental data and in line with previous theoretical results using different DFT methods [49,50]. Thus, the maximum deviation for bond lengths is only 0.012 Å. On the other hand, our computational protocol, in line with other DFT methods used earlier [49], predicts a perfectly linear <Ru-N-O bond angle (180°) in contrast to the X-ray experimental value, deviating from linearity (174.4°).

Next, the GS-optimized geometry of the [(NH_3_)_5_Ru(NO)]^3+^ model complex, using the present DFT method, is also in line with the experiment outlined in [51], with bond lengths differing at a maximum of 0.04 Å. Again, in line with other previous DFT studies, the <Ru-N-O bond angle is predicted to be nearly linear (178.8°) while experiment gave a higher deviation from linearity (172.8°).

Finally, the GS-optimized geometry of the [(CN)_5_Fe(NO)]^2−^ Fe(II) complex is in very good agreement with experiment, with bond lengths differing at most 0.046 Å and a predicted <Fe-N-O bond angle (180°) in excellent agreement with the X-ray value (179.8°) [52]. It bears noting that the optimized geometries obtained upon employing a higher-level DFT method, namely, the PBE0/LANL2TZ(M)U6-311G(2d,2p)(E)/PCM(Water), gave only marginal differences of ≤0.02 Å and ≤2°, confirming that the LANL2DZ-based structures used throughout the work are chemically reliable and that the mechanistic conclusions are basis-set independent.

#### 2.1.2. Ground-State-Optimized Geometries of the [(L)Ru(NO)(HS)@M_x_M′_20−x_] Complexes

**Figure 2:**
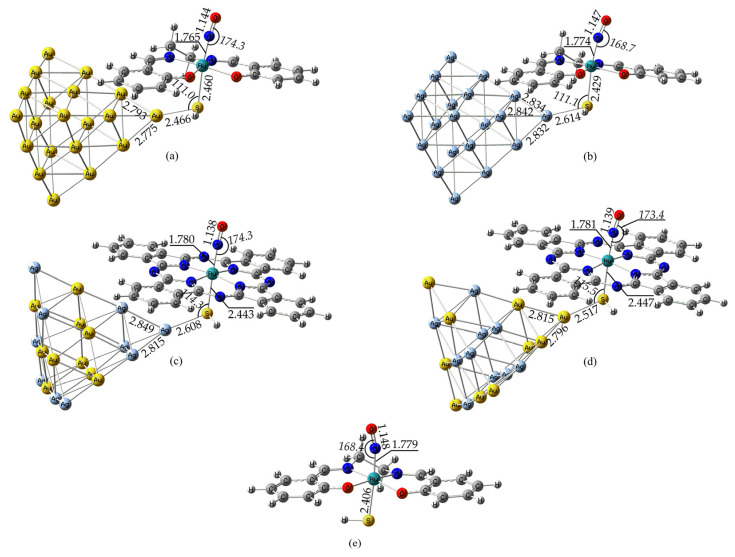
Optimized GS geometries for some selected representative complexes: (**a**) [(Salen)Ru(NO)(HS)@Au_20_], (**b**) [(Salen)Ru(NO)(HS)@Ag_20_], (**c**) [(Pc)Ru(NO)(HS)@Ag_10_Au_10_] (Type-3 isomer [41], (**d**) [(Pc)Ru(NO)(HS)@Au_10_Ag_10_] (Type-5 isomer, [41]), and (**e**) [(Salen)Ru(NO)(HS) complex calculated at the PBE0/LANL2DZ(Ru)U6-31G(d,p)/PCM(water) level.

Having established the validity of our computational protocol, we proceed with the optimization of [(L)Ru(NO)(HS)@M_x_M′_20−x_] systems under study. The GS-optimized geometries for some representative [(L)Ru(NO)(HS)@M_x_M′_20−x_] hybrid complexes are depicted schematically in Figure 2, while the rest are given as Supplementary Materials (Figures S1–S6). Inspection of Figure 2 and Figure S1–S6 reveals that the nature of the NP does not significantly affect the structural parameters. Thus, the bond lengths within the Ru-N-O framework, i.e., the *R*_e_(N-O) and *R*_e_(Ru-N) bond lengths, show little variation upon changing either the NP or the equatorial ligand, lying in the ranges 1.137–1.148 Å and 1.759–1.785 Å, respectively. The same also holds for the *R*_e_(Ru-S) bond lengths lying in the range 2.429–2.517 Å. The most striking differences are observed for the bond distances connecting the NP to the thiol ligand, i.e., the *R*_e_(S-M) bond lengths, as well as the <Ru-N-O bond angle. Thus, upon changing the NP, the *R*_e_(S-M) changes significantly by up to 0.15 Å while <Ru-N-O changes up to 6°. Nonetheless, in all cases, the <Ru-N-O bond angle remains in the range 168–174°, indicating that the NO axial ligand should have a cationic form, consistent with a {RuNO}^6^ Enemark—Feltham notation. It should also be noted that the *R*_e_(N-O) values are significantly lower than those found for the ‘free’ NO^+^ cation, being 1.067 Å at the same level of theory. This reflects the typical behavior exhibited by {Ru–NO}^6^ systems, where, due to the π-backbonding from the metal, the NO π* orbital weakens and subsequently lengthens the N-O bond, imparting partially neutral or even anionic NO character with a delocalized nature of the bonding for the Ru-N-O framework. Finally, it bears noting that the structural parameters pertaining to the equatorial ligands remain practically unaffected.

Next, in order to check the effect of the NP, we also calculated the geometry of the [(Salen)Ru(NO)(HS)]-representative complex. Inspection of Figure 2 reveals that in this parent complex, the structural parameters do not change significantly upon comparison with the respective hybrid complexes bearing the M_20_ gold or silver NPs.

#### 2.1.3. Metastable-State-Optimized Geometries of the [(L)Ru(NO)(HS)@M_x_M′_20−x_] Complexes

**Figure 3:**
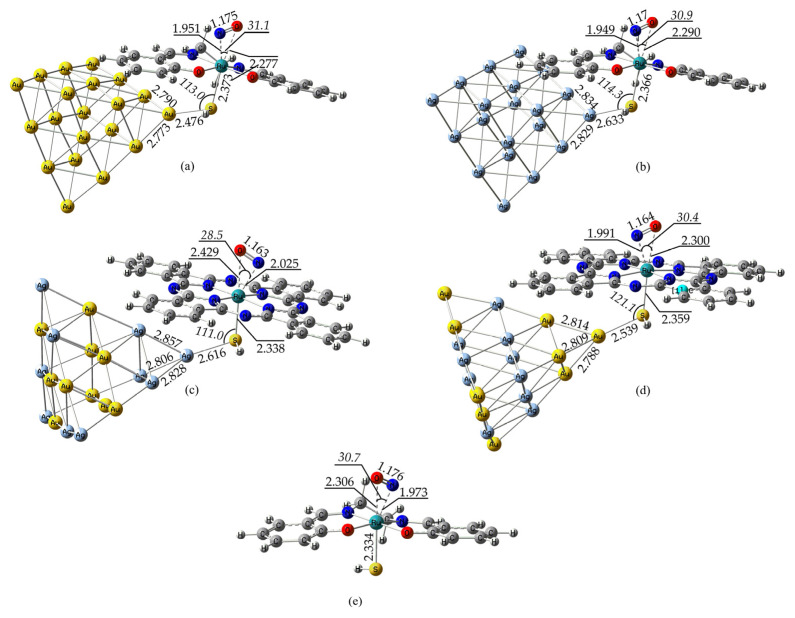
Optimized metastable state (MSII) geometries for some selected representative complexes: (**a**) [(Salen)Ru(NO)(HS)@Au_20_], (**b**) [(Salen)Ru(NO)(HS)@Ag_20_], (**c**) [(Pc)Ru(NO)(HS)@Ag_10_Au_10_] (Type-3 isomer [41], (**d**) [(Pc)Ru(NO)(HS)@Au_10_Ag_10_] (Type-5 isomer, [41]), and (**e**) [(Salen)Ru(NO)(HS)] complexes calculated at the PBE0/LANL2DZ(Ru)U6-31G(d,p)/PCM(water) level.

**Figure 4:**
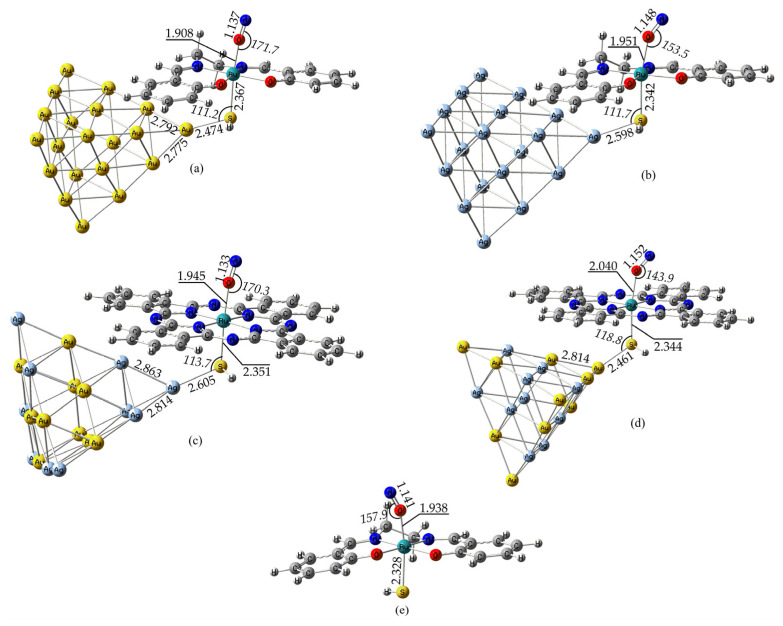
Optimized metastable state (MSI) geometries for some selected representative complexes: (**a**) [(Salen)Ru(NO)(HS)@Au_20_], (**b**) [(Salen)Ru(NO)(HS)@Ag_20_], (**c**) [(Pc)Ru(NO)(HS)@Ag_10_Au_10_] (Type-3 isomer [53], (**d**) [(Pc)Ru(NO)(HS)@Au_10_Ag_10_] (Type-5 isomer, [53]), and (**e**) [(Salen)Ru(NO)(HS)] complexes calculated at the PBE0/LANL2DZ(Ru)U6-31G(d,p)/PCM(water) level.

During the NO BLI process and upon irradiation, two metastable species were formed, namely, the MSII and the MSI; we set out to study these systems by employing the PBE0/LANL2DZ(M)U6-31G(d,p)(E)/PCM(water) computational protocol. The optimized geometries of the MSII and MSI species formed by the representative complexes depicted in Figure 2 are shown in Figure 3 and Figure 4, respectively. For comparison, we also give the MSII and MSI species formed by the model complexes in Figure 1. In addition, Figure 3 and Figure 4 also contain the MSII and MSI species for the parent salen complex in order to study the NP effect on the NO BLI process.

Examining Figure 3 reveals that there are significant structural differences between the MSII and GS species. To begin with, the n^2^-NO ligand in the former is lengthened by about 0.3 Å as compared to the respective NO ligand in the latter. This is probably due to an increased Ru(dπ) → NO(π*) backdonation relative to the linear N-bound NO in GS. The Ru-N and Ru-O distances between the n^2^-NO ligand and the Ru metal center are quite different, with the latter being significantly longer than the former. As a result, the N donor atom of the n^2^-NO ligand lies nearer to the Ru metal center in all MSII species examined. The n^2^-NO ligand forms a very acute <Ru-N-O bond angle calculated to be around 30°. 

The very acute ∠Ru–N–O observed for the η^2^-NO coordination is indicative of the side-on overlap of the Ru dπ set with the two orthogonal NO π* orbitals. This Dewar–Chatt–Duncanson-type interaction increases π* population, lowers the N–O bond order, and favors a bent/side-on geometry. In Enemark–Feltham terms, the description shifts away from a {RuNO}^6^, NO^+^-like picture toward higher {RuNO}ⁿ electron counts with appreciable NO^−^/NO^•^ character. Another striking structural difference is the shorter Ru-S bonds observed for the MSII species as compared to the GS species. This Ru-S bond shrinkage indicates that the side-on NO ligand should exhibit a weaker trans influence as compared to the linear n^1^-NO ligand in GS species. No other significant structural changes could be observed, and both the M-S distance connecting the NPs with the Ru metal center via the thiol ligand, as well as the NPs and the equatorial ligands, remain practically unaffected upon going from GS to MSII species. Finally, the same pattern of structural changes between GS and MSII could also be observed for the parent salen complex.

For comparison, we have also calculated the MSII species for the model complexes (Figure 1). In all three model complexes examined, the side-on n^2^-NO ligand exhibits a slightly shorter N-O bond length. The slightly shorter N–O bond lengths calculated for the model complexes reflect the weaker Ru → NO π-backdonation, whereas elongation in the Ru–NO@M_20_ hybrids demonstrates that coupling with the metallic nanocluster enhances π* population on NO, evidencing the role of the nanoparticle-induced modulation of NO bonding. In three model complexes, the <M-N-O bond angle is acute and similar to that found for the hybrid complexes, i.e., ca. 30–35°. Therefore, the NPs should primarily be involved in modulating the extent of the Ru → NO π* backdonation, rather than changing the n^2^ bonding geometry. The magnitude of the <M-N-O bond angles, being similar for all complexes studied, with or without NPs, induces optimal overlap between the Ru dπ (t_2g_) orbitals and the NO π* orbitals, characteristic of the n^2^-NO bonding mode.

Finally, Figure 3 also depicts the optimized geometry of the MSII [(Salen)Ru(NO)(HS)] species, being the parent complex of the [(Salen)Ru(NO)(HS)@Au_20_] hybrid complex, without bearing the Au_20_ NP. Remarkably, the <Ru-N-O bond angle as well as the N-O bond length of the n^2^-NO ligand are quite similar to those found in the respective hybrid complex. Thus, we could conclude that the acute <M-N-O, observed across all systems studied (i.e., the model complexes, the hybrid complexes, and the parent complex without NP), indicates the optimal orbital overlap for this geometrical arrangement, while the N-O bond length should basically be modulated by the overall ligand field strength of these complexes.

Next, let us examine the optimized geometries of the MSI species with a nitrosyl ligand coordinated to the Ru metal center via a linear n^1^-ON mode. Inspecting Figure 4, we observe that the <Ru-N-O bond angle varies significantly depending upon the nature of the NP. Accordingly, in the [(Salen)Ru(NO)(HS)@Au_20_] hybrid, bearing the pure gold NP, the <Ru-N-O bond angle remains nearly linear, similar to that found for the GS species (171° vs. 174°). In contrast, in the [(Salen)Ru(NO)(HS)@Ag_20_] hybrid, bearing the pure silver NP, the <Ru-N-O bond angle significantly departs from linearity, being around 154°. Although this angle is generally found to be more acute in the Ag_20_ systems in GS as compared to their Au_20_ NP counterparts, its value still points to a cationic NO^+^ ligand. However, for the MSI species with Ag_20_, this angle becomes even more acute, indicating that the nitrosyl ligand should exhibit a NO^−^/NO^•^ character.

Remarkably, the MSI [(Salen)Ru(NO)(HS)] species exhibit a geometry similar to that found for the MSI [(Salen)Ru(NO)(HS)@Ag_20_] and MSI Type-5 isomeric [(Pc)Ru(NO)(HS)@Au_10_Ag_10_] species, and especially, a <Ru-N-O bond angle equal to 158°, significantly departing from linearity and signifying a NO^−^/NO^•^ character. This, in contrast to the MSI [(Salen)Ru(NO)(HS)@Au_20_] and Type-3 isomeric [(Pc)Ru(NO)(HS)@Ag_10_Au_10_] species, where this angle is near linear, signifying the effect of the NP nature on the N-O bond length.

### 2.2. Vibrational Spectra

**Figure 5:**
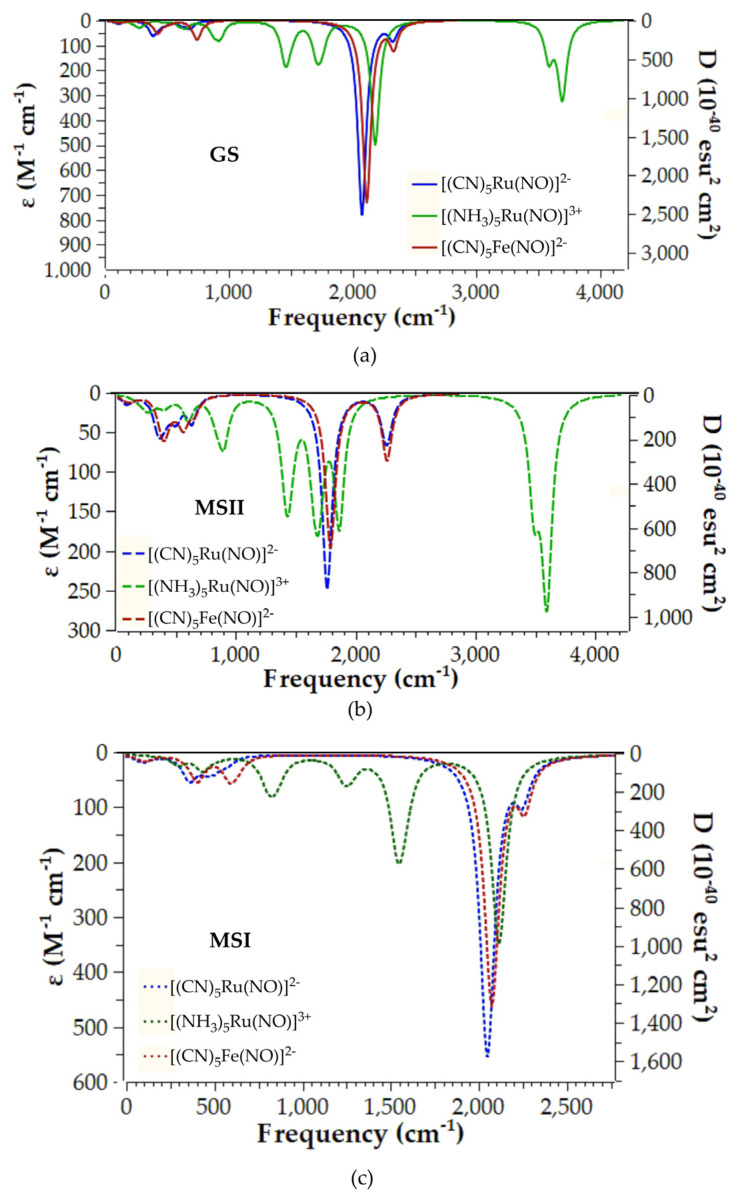
Vibrational spectra of (**a**) GS, (**b**) MSII, and (**c**) MSI species of the model complexes calculated at the PBE0/LANL2DZ(Ru)U6-31G(d,p)/PCM(water) level.

**Figure 6:**
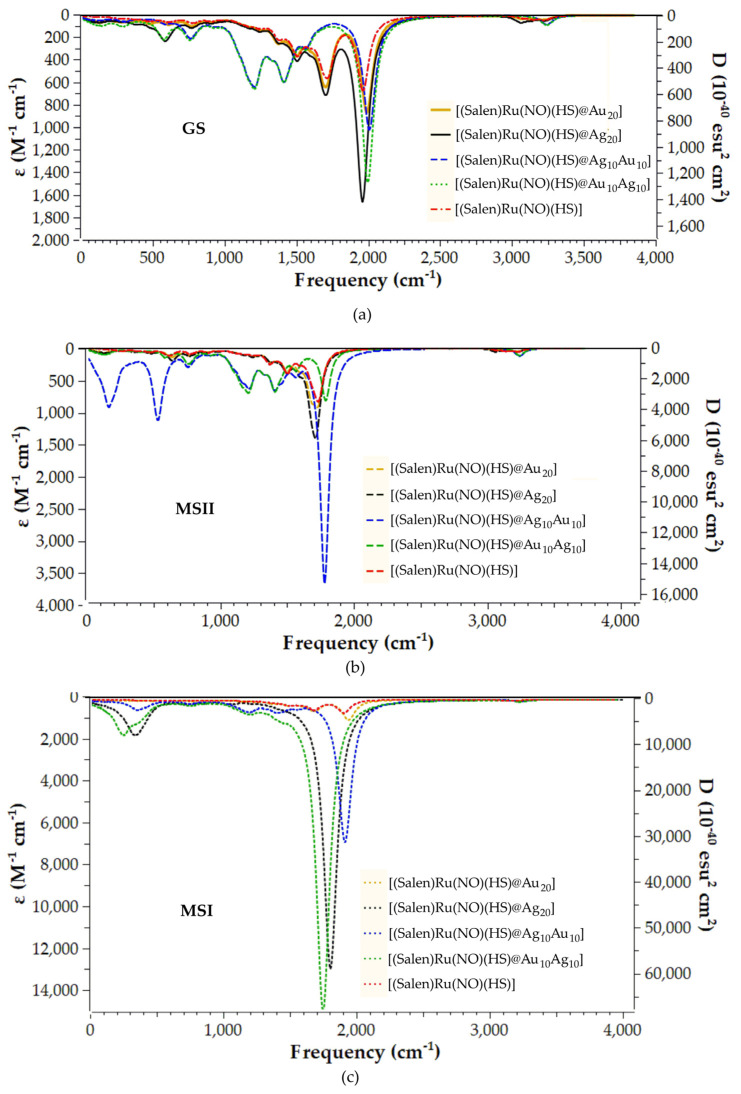
Vibrational spectra of (**a**) GS, (**b**) MSII, and (**c**) MSI species of the representative hybrid and parent complexes calculated at the PBE0/LANL2DZ(Ru)U6-31G(d,p)/PCM(water) level.

The vibrational spectra of all species studied were calculated employing the PBE0/LANL2DZ(M)U6-31G(d,p)(E)/PCM(water) computational protocol in order to assist in identifying the metastable species MSI/MSII in a future experimental study of these hybrid complexes. Figure 5 and Figure 6 depict the vibrational spectra for the model complexes and their respective representative hybrid and parent complexes.

Table 2 presents the NO stretching vibrational frequency, v_s_(N-O), for all complexes under study observed in the simulated vibrational spectra. Finally, the simulated vibrational spectra for the rest of the hybrid complexes under study are depicted in Figures S7–S12 in the Supplementary Materials.

Let us first examine the simulated vibrational spectra of the three model complexes. Inspection of Figure 5a reveals that the GS species exhibit basically one intense band in the region 2000–2300 cm^−1^. This band corresponds to the v_s_(N-O) stretching mode, and perusal of Table 2 shows that it is in excellent agreement either with experiment or with previous theoretical studies using a different DFT method. The same also holds true for the calculated vibrational spectra of both the MSII and MSI species, which are depicted in Figure 5b and Figure 5c, respectively. More specifically, the simulated vibrational spectra of the [(CN)_5_Ru(NO)]^2−^ and [(CN)_5_Fe(NO)]^2−^ model complexes in their MSII state (blue and red dashed lines in Figure 5b) show basically one intense band, attributed to the v_s_(N-O) vibrational mode. In contrast to GS, this vibrational mode appears well below 2000 cm^−1^ in line with previous available experimental/theoretical data (Table 2). On the other hand, the simulated vibrational spectrum of the [(NH_3_)_5_Ru(NO)]^3+^ model complex differs from those calculated for the other two model complexes, showing a multitude of bands. Most of them are related to the N-H bonds of the NH_3_ ligands. The v_s_(N-O) vibrational mode for [(NH_3_)_5_Ru(NO)]^3+^ model complex appears in the same region as for the other two model complexes, i.e., around 1783 cm^−1^, in line with previous theoretical data (Table 2).

Next, the simulated vibrational spectra of all three model complexes in the MSI state are much simpler and similar to those calculated for the GS. Accordingly, they mainly exhibit a very intense band around 2000 cm^−1^ corresponding to the v_s_(N-O) vibrational mode, which is in line with previously reported data (Table 2).

Finally, let us now proceed to analyzing the simulated vibrational spectra of the hybrid complexes in GS, MSII, and MSI states (Figure 6). Examining Figure 6a, we see that the simulated vibrational spectra for the GS of complexes bearing either gold or silver NPs, as well as those calculated for the parent Ru complex (without NP), are quite similar, showing one intense band just below 2000 cm^−1^ (see also Table 2). The same could also be observed for the [(Salen)Ru(NO)(HS)] parent complex. This band corresponds in all cases to the v_s_(N-O) vibrational mode, similar to the model complexes (vide supra), appearing, however, at slightly lower energies. This red shift is also observed in the simulated vibration spectra of both MSII and MSI states of the Ru hybrids and parent complexes. It should be stressed here that the observed v_s_(N-O) values are primarily determined by the {MNO} electron count and the Ru–NO bonding motif. Thus, the red shift in the v_s_(N-O) values as compared to the model systems could be explained by the differences in the trans effect upon introducing the thiol-bridged gold/silver NPs, resulting in an increase in Ru → NO backdonation and a lowering of v_s_(N-O).

### 2.3. Absorption Spectra

**Figure 7:**
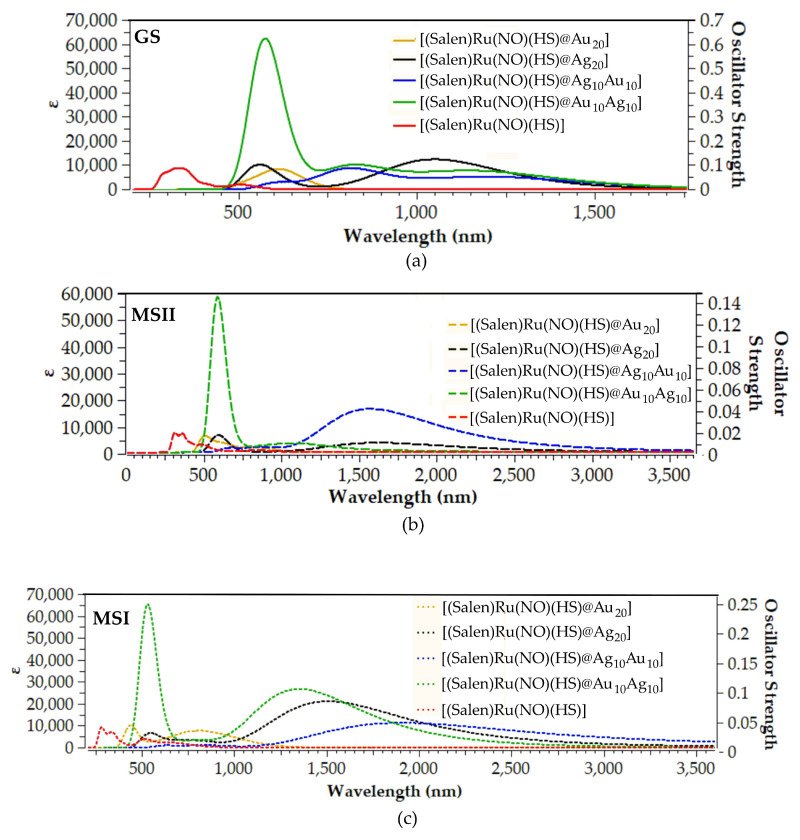
Absorption spectra of (**a**) GS, (**b**) MSII, and (**c**) MSI species of representative hybrid and parent complexes calculated at the TD-PBE0/LANL2DZ(Ru)U6-31G(d,p)/PCM(water) level.

The use of the hybrid materials as holographic theranostics, upon study, requires knowledge of their absorption spectra. Accordingly, we simulated the UV-Vis absorption spectra of the hybrid complexes under investigation. Figure 7 depicts the absorption spectra of some representative hybrid complexes in their GS, MSII, and MSI states, calculated at the TD-PBE0/LANL2DZ(Ru)U6-31G(d,p)/PCM(water) level.

The simulated absorption spectra for the rest of the complexes under investigation are given in Figures S13–S18 in the Supplementary Materials.

Upon examination of Figure 7a, we observe that the GS simulated absorption spectra of the representative hybrid and parent complexes exhibit bands that span across the visible up to the NIR region. The hybrid complexes exhibit two bands, i.e., a low energy in the visible region and a high energy within the NIR region. A notable exception is the [(Salen)Ru(NO)(HS)@Au_20_] hybrid complex, bearing the Au_20_ NP, exhibiting only one band in the visible region. Finally, the parent Ru complex, bearing no NP, also shows two bands, both in the visible region.

**Figure 8:**
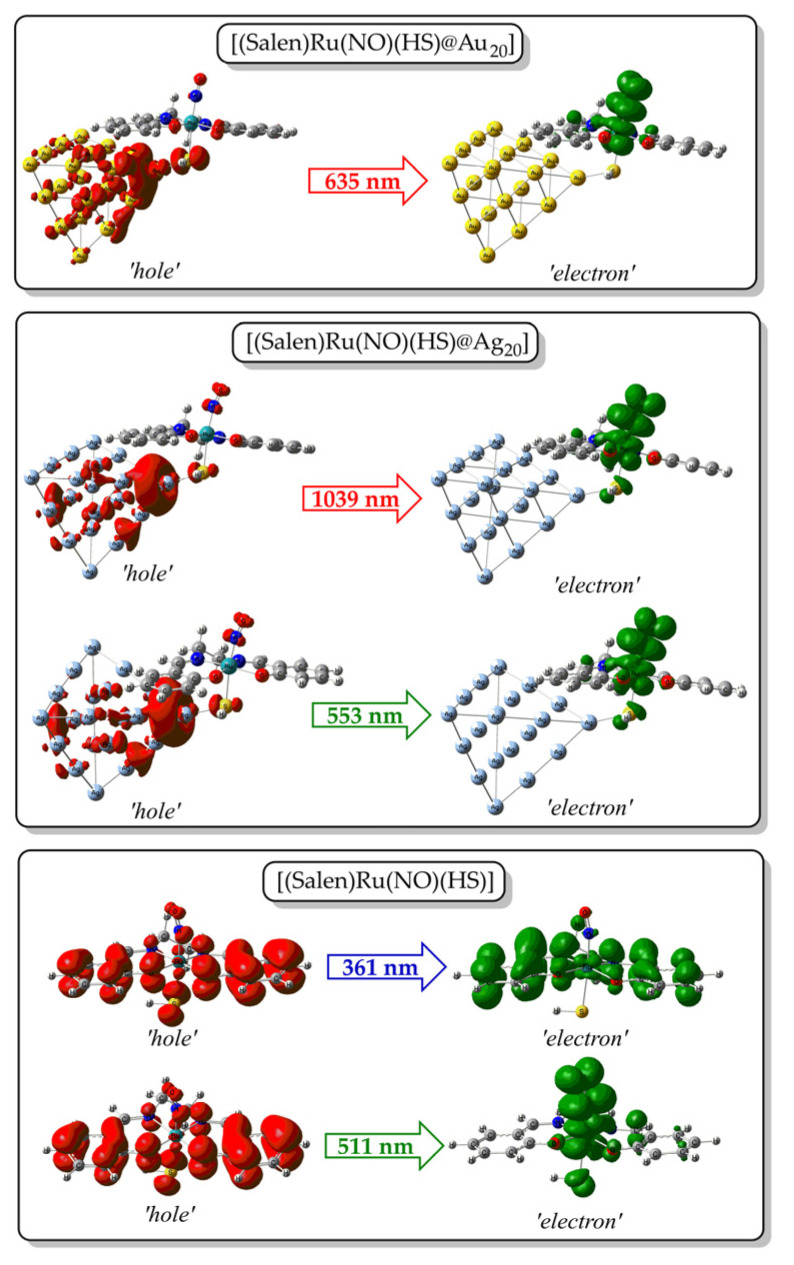
Three-dimensional surfaces of MOs involved in relevant electronic excitations in the simulated absorption spectra of the GS species of the representative hybrid and parent complexes.

The band around 635 nm observed in the simulated absorption spectrum of [(Salen)Ru(NO)(HS)@Au_20_] is dominated by an electronic transition peaking at 635 nm, which in turn arises from a HOMO → LUMO excitation. To assist in the band assignment, we calculated the ‘hole’/’electron’ distributions upon excitation, and Figure 8 depicts the respective 3D surfaces. Accordingly, for the electronic transition at 635 nm, electron density is transferred from the Au_20_-SH unit towards the Ru-N-O unit. Thus, the band at 635 nm could be assigned as MLCT/MM’CT/LL’CT.

Upon replacing the Au_20_ NP with either Ag_20_ or mixed Au_10_Ag_10_ NPs, the simulated absorption changes significantly, exhibiting two instead of one main absorption bands. Thus, in the simulated absorption spectra of the silver-containing NPs, the high-energy band within the visible region, found also for the [(Salen)Ru(NO)(HS)@Au_20_] hybrid complex, is retained, but also a second, low-energy band appears well within the NIR region. For example, the [(Salen)Ru(NO)(HS)@Ag_20_] hybrid complex exhibits a high-energy band around 550 nm and a low-energy band around 1050 nm. The former is dominated by an electronic transition at 553 nm, while the latter is dominated by an electronic transition at 1039 nm. Based upon the ‘hole’/’electron’ distributions, both of these two transitions exhibit an MLCT/MM’CT character, with electron density flowing from the Ag_20_ NP towards the Ru-N-O unit. It bears noting that the same behavior is also observed for the systems bearing mixed gold/silver NPs with a remarkable, almost 6-fold intensity increase for the high-energy band in the visible region for the [(Salen)Ru(NO)(HS)@Au_10_Ag_20_] Type-5 isotopomer.

On the other hand, the representative [(Salen)Ru(NO)(HS)] parent complex also exhibits two bands, one low-energy band around 350 nm, well within the UV region, and a second one around 520 nm in the visible region. The ‘hole’/’electron’ distributions for these transitions (Figure 8) reveal that the high-energy band in the UV region is assigned as intraligand (IL), while the low-energy band in the visible region could be assigned as LMCT/LL’CT. Obviously, the presence of the NP drives the absorptions towards red/NIR.

### 2.4. Photo-Rearrangement Mechanism

**Figure 9:**
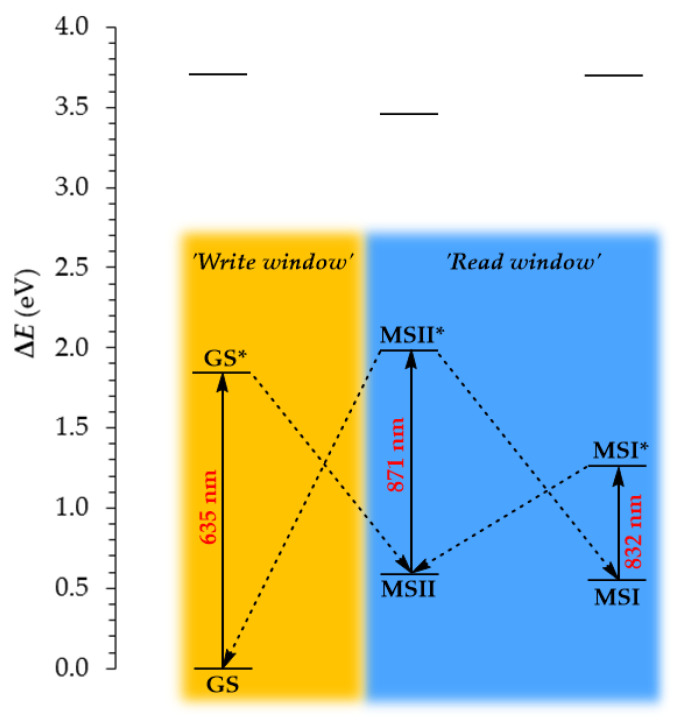
Photo-rearrangement mechanism in [(Salen)Ru(NO)(HS)@Au_20_] hybrid complex. Photoexcitation (solid arrows) populates metastable MS states, while dashed arrows denote thermal or vibronic back-conversion.

Let us now delve into the photo-rearrangement mechanism for the photogeneration of the nitrosyl linkage isomers, necessary for realizing the materials-based holograms. We will at first examine this mechanism for the representative [(Salen)Ru(NO)(HS)@Au_20_] hybrid complex, and then we will extract trends to reveal the optimal candidate complex for use as a holography material. Figure 9 depicts the photo-rearrangement mechanism for nitrosyl-species generation by the [(Salen)Ru(NO)(HS)@Au_20_] hybrid complex, upon irradiation. It should be noticed that the same mechanistic pattern applies to all complexes under study, and only the exact excitation energies vary, as reported in Table 3.

For the [(Salen)Ru(NO)(HS)@Au_20_] hybrid, the lowest bright excitation occurs at 635 nm (S_2_, f = 0.08), whereas S_1_ at 661 nm is essentially dark, consistent with spin- and Laporte-forbidden character of low-lying d–d and back-bonding excitations in second-row Ru complexes. Accordingly, the GS* in Figure 2 corresponds to the optically significant S_2_ state. Next, photoexcitation is followed by an ultrafast internal conversion as well as by spin–orbit-induced intersystem crossing populating the S_1_ and T_1_ states, respectively. Nonetheless, the S_2_ → S_1_ → T_1_ relaxation cascade culminates the system in the MSII state, funneling towards the N → O linkage isomerization and leading to the ON MSI form responsible for the holographic signal. The GS → GS* transition is the so-called, in holography, ‘*write window*’, which encompasses the photochemical/photoinduced transitions between the GS and the MS states. Thus, the ‘*write window*’ for the [(Salen)Ru(NO)(HS)@Au_20_] hybrid complex is within the red and specifically in the 620–650 nm region (orange shaded region in Figure 9). On the other hand, the optically significant, ‘bright excited states’, for the metastable MSII and MSI species are the S_1_ (*f* = 0.01, MSII*) and S_4_ (*f* = 0.05, MSI*), respectively. The MSII → MSII* and MSI → MSI* excitations could occur within the 830–870 nm region, being the so-called, in holography, ‘*read window*’ (Figure 9). It bears noting that a ‘*read window*’ in the NIR region is very advantageous in theranostics due to its favorable tissue optics and ease of application using commodity lasers, while, for a possible ‘holographic theranostic’, it offers good separation from the ‘write window’ (for non-destructive read).

Next, we will examine the non-radiative relaxation channels between the GS, MSII, and MSI species. These are the possible thermally activated, non-radiative channels (dashed arrows, Figure 9) between the GS, MSII, and MSI states and the electronically excited ground state or metastable species. For example, the GS* → MSII channel describes the population of the MSII state upon vibronic coupling of the electronically excited ground state, GS*, with the MSII state, thus initiating the photo-isomerization step. On the other hand, the MSII* → GS channel corresponds to the thermal back-conversion, leading to the self-erasure of the holographic pattern. Finally, both the MSI* → MSII and MSII* → MSI processes are responsible for the bidirectional internal conversion between the metastable species. The non-radiative relaxation pathways could be estimated, at least semi-quantitatively, upon calculation of the adiabatic energy separations between minima and the absorption centroids spacing and overlap, as well as from the coupling strength, based on v_s_(N-O) and v_s_(Ru-N) stretching vibrational frequencies. In this way, we could extract trends amongst the systems under study with respect to their potential for use in materials-based holography. It bears noting that, according to the energy gap law [55,56], larger energy gaps and smaller spectral overlaps correlate with slower non-radiative rates. Table 3 presents the parameters related to the radiative and non-radiative decay processes occurring during the photo-rearrangement mechanism, for all systems under study, which are similar to those depicted in Figure 9 for the [(Salen)Ru(NO)(HS)@Au_20_] representative complex, as well as the parameters relevant to their potential as holography materials. The table summarizes for each complex: the principal bright excitation wavelengths, λ(GS → GS*), λ(MSII → MSII*), and λ(MSI → MSI*); the corresponding oscillator-strength sums Σf_i_; the adiabatic interstate energy separations ΔE(GS–MSII) and ΔE(MSII–MSI); the vibrational coupling indicators Δν(N–O) and Δν(Ru–N/O); and the derived non-radiative coupling indices (NRCIs) and stability scores, S. These quantities collectively describe the radiative (bright-state) and non-radiative (IC/ISC) channels involved in the GS → MSII → MSI photo-rearrangement sequence. The positions of the bright transitions define the write and read spectral windows relevant for materials-based holography. Large ΔE values, reduced spectral overlap, and moderate vibrational coupling signify efficient population trapping in the metastable manifolds, whereas significant NIR absorption identifies systems suitable for theranostic or holographic–theranostic applications combining optical data storage and photoresponsive functionality. The three highest-ranking entries (gray-shaded) correspond to complexes predicted to exhibit the most favorable balance of optical brightness, stability, and non-radiative channel control. Amongst the series of complexes given in Table 3, the most favorable candidates, combining strong radiative activity (large Σf_i_), balanced adiabatic energy separations, and moderate vibrational coupling—thereby offering the optimal trade-off between optical brightness, metastable stability, and controlled non-radiative decay for holography-based theranostic applications—follow the increasing performance trend [(Salen)Ru(NO)(HS)@Au_20_] > [(Porph)Ru(NO)(HS)@Au_20_] > [(Salen)Ru(NO)(HS)@Ag_20_].

### 2.5. Dynamic Polarizability and Refractive-Index Modulation in GS, MSII, and MSI

To gain insight into the optical response of the photoactive Ru–NO systems, frequency-dependent isotropic polarizabilities α(ω) were calculated for GS as well as for MSII and MSI metastable states employing the TD-PBE0/def2-TZVP method in water solvent. From the computed α(ω) values, the refractive index η(λ) was derived using the Lorentz–Lorenz relation, which connects molecular polarizability to macroscopic optical density [57,58,59]. Under the assumption of constant molecular number density N (identical for the three states of a given compound), the refractive index can be expressed as:

$$\eta (\lambda )\;=\;\sqrt{\;\frac{1\;+\;2f(\lambda )}{1\;-\;f(\lambda )}}$$

where

*f*(*λ*) = (4/3)π*Nα*(*ω*)

The difference between the refractive indices of the metastable and ground states provides a direct measure of the optical contrast generated by the photoisomerization process:

Δ*η*(λ) = *η_MSII,MSI_*(*λ*) − *η_GS_*(λ)

Since the density term cancels when comparing isomers of similar molecular volume, the relative Δη(λ) values are directly proportional to Δα(ω) and may thus be estimated on a relative scale. Table 4 summarizes the results obtained for the representative hybrid [(Salen)Ru(NO)(HS)@Au_20_] complex, which was identified as the most efficient system from the previous analysis, and for the benchmark nitroprusside [Fe(CN)_5_NO]^2−^, which is widely used as a reference material for NO-based holographic storage. The computed values show a clear and consistent enhancement of refractive-index modulation in the Au_20_–salen complex across the visible and near-infrared regions.

## 3. Methods and Materials

All calculations were performed using the Gaussian 16 suite of programs [60]. The geometry of all species was fully optimized without imposing any symmetry constraints, employing the Perdew, Burke, and Ernzerhof PBE0 functional [61,62,63,64,65,66]. The LANL2DZ, effective core potential (ECP) basis set was used for the Ru and Au metal atoms in conjunction with the 6-31G(d,p) basis set for the non-metal atoms [67,68]. Water solvent effects were taken into account using the polarizable continuum model (PCM) as implemented in the Gaussian 16 suite of programs [69]. All optimized structures were confirmed as minima no imaginary frequencies (NImag = 0). Hence, the computational protocol employed in the optimization of the geometries and the subsequent vibrational modes calculations is symbolized as PBE0/LANL2DZ(Ru,Au)U6-31G(d,p)(E)/PCM(Water). Electronic excitations were computed using the time-dependent DFT (TDDFT) method [70] employing the same computational protocol as in geometry optimizations upon including 30 singlet excited states. Oscillator strengths and transition energies were used to analyze spectral regions relevant to the write/read windows of the photo-linkage process. Frequency-dependent polarizabilities α(ω) were calculated using CPHF=RdFreq at representative wavelengths (400–1000 nm), providing the dispersion of α(ω) necessary to evaluate refractive indices through the Lorentz–Lorenz relation. Relative refractive-index changes (Δn) were obtained by comparing GS, MSII, and MSI states at identical frequencies. Polarizabilities were calculated using a higher quality basis set, namely, the def2-TZVP [71], along with the PBE0 functional and taking into account the water solvent effects as well. All polarizability-derived refractive-index values reported in Table 4 were obtained under a uniform molecular number density assumption, which allows for the direct comparison between systems of similar molar volume. This approach has been successfully employed in previous studies of molecular photochromic materials and provides reliable qualitative trends for refractive-index modulation [58,59]. Also, it should be noted that for polarizability calculations, we used an implicit solvation environment to emulate the aqueous medium commonly used in biological and holographic systems. The solvent was modeled as water using the polarizable continuum model (PCM) with a static dielectric constant of ε_0_ = 78.36 and an optical dielectric constant of ε ∞ 1.78, corresponding to its refractive index (*n* ≈ 1.33 at 298 K). These parameters account, respectively, for the slow orientational and fast electronic components of solvent polarization and were used consistently in all frequency-dependent polarizability and refractive-index calculations. The chosen dielectric parameters for water corresponded to experimentally validated dispersion data in the visible and near-UV ranges [72]. In order to assess the sensitivity of Ru–NO metrics to the basis-set quality, benchmarking calculations were performed at a higher level of DFT theory, namely, the PBE0/LANL2TZ(Ru)/6-311G(2d,2p)/PCM(water) level [73,74,75]. Two out of the three reference Ru–NO complexes with crystallographic geometries converged smoothly at this higher level, and in both cases the resulting bond distances differed from the LANL2DZ results by ≤ 0.01–0.02 Å, with a ≤ 2° variation in the Ru–N–O angle. These values match the known DFT performance window for Ru–NO systems (typically 0.02–0.06 Å and 4–10° [76,77,78]), supporting the robustness of the original PBE0/LANL2DZ protocol. Attempts to extend the full LANL2TZ/6-311G(2d,2p) protocol to the [(NH_3_)_5_Ru(NO)]^3+^ complex and to a representative hybrid complex, [(Salen)Ru(NO)(HS)@Au_20_], were unsuccessful due to SCF/optimization instability—a known limitation for highly charged cationic complexes and large metal cluster systems with highly delocalized orbitals. Given that the systems which did converge showed only minute differences at the higher level, and that the observed GS → MSI → MSII distortions are one to two orders of magnitude larger, we conclude that the mechanistic trends reported herein are insensitive to further basis-set enlargement. Full numerical comparisons are provided in Table S1 of the Supplementary Material. Cartesian coordinates and relevant energetic data corresponding to the optimized geometries of all species studied are given in Table S2 of the Supplementary Material.

## 4. Conclusions

The present work establishes a consistent framework linking the electronic and structural aspects of Ru–NO photo-linkage isomerism to measurable optical properties relevant for holography. The ground (GS), side-on (MSII), and O-bound (MSI) states were characterized through geometry optimization, frequency analysis, and TDDFT spectroscopy. Comparison of the oscillator strengths, adiabatic energy differences, and shifts in *ν*_s_(N–O) and *ν*_s_(Ru–NO) allowed for a set of practical descriptors to be defined for radiative activity, metastable-state stability, and vibrational coupling strength. Among all complexes examined, [(Salen)Ru(NO)(HS)@Au_20_], [(Porph)Ru(NO)(HS)@Au_20_], and [(Salen)Ru(NO)(HS)@Ag_20_] emerge as the most promising materials for NO-based holography and related photonic or theranostic applications. These systems exhibit accessible isomerization energetics and optical windows extending into the red and near-infrared regions, which are favorable for both data storage and biological compatibility. A direct comparison between [(Salen)Ru(NO)(HS)@Au_20_] and the well-known nitroprusside complex was performed using frequency-dependent polarizabilities. The conversion of α(ω) into refractive indices through the Lorentz–Lorenz relation revealed that the Au_20_–salen hybrid provides a significantly stronger optical response. Across the visible and near-infrared regions, the refractive-index modulation Δ*n*(*λ*) varies from 0.04 to 0.06 for the hybrid, compared with 0.01–0.02 for nitroprusside. The largest difference, near 635 nm, coincides with the main electronic absorption associated with the GS → GS* transition. This correlation indicates that the nanoplasmonic coupling not only strengthens the optical response but also enhances spectral tunability. Overall, the results highlight that embedding Ru–NO linkage-isomer systems in metallic nanoclusters is an effective route to amplify both photochemical and optical characteristics. The derived relationships between adiabatic energy gaps, oscillator strengths, vibrational shifts, and refractive-index modulation can serve as practical guidelines for designing new molecular materials capable of high-contrast optical recording and holography-based theranostic functions.

## Figures and Tables

**Table 1:** Comparisons of selected structural parameters of the model complexes used for benchmarking the PBE0/LANL2DZ(Ru)/6-31G(d,p)/PCM(water) computational protocol ^1^.

Str. Par.	[(CN)_5_Ru(NO)]^2−^			[(NH_3_)_5_Ru(NO)]^3+^			[(CN)_5_Fe(NO)]^2−^		
	Our Method	Exptl. ^1^	Other DFT ^2^	Our Method	Exptl. ^1^	Other DFT ^2^	Our Method	Exptl. ^1^	Other DFT ^2^
*R*_e_(N-O)	1.138	1.130	1.166	1.123	1.172	1.129	1.135	1.133	1.162
*R*_e_(Ru-NO)	1.785	1.773	1.786	1.776	1.770	1.819	1.620	1.666	1.630
*R*_e_(Ru-L)_trans_	2.056	2.051	2.099	2.128	2.020	2.194	1.920	1.926	1.958
*R*_e_(Ru-L)_cis_	2.057	2.064	2.113	2.136	2.100	2.211	1.913	1.931	1.965
<Ru-N-O	180.0	174.4	180.0	178.8	172.8	179.3	180.0	179.8	180.0

^1^ Data taken from ref. [49]. ^2^ Calculated using the B3LYP/DZVP method [49].

**Table 2:** N-O stretching vibrational frequencies, *v*_s_(NO) (in cm^−1^) of the NO ligand, calculated at the PBE0/LANL2DZ(Ru)/6-31G(d,p)/PCM(water) level.

Species	*v*_s_(NO) ^1^		
	GS	MSII	MSI
[(CN)_5_Ru(NO)]^2−^	1925 (**1926**) ^2^	1771 (*1641*) ^3^	1950 (*1917*)
[(NH_3_)_5_Ru(NO)]^3+^	2025 (**1950**, *2089*)	1783 (*1865*)	2013 (**1823**, *2019*)
[(CN)_5_Fe(NO)]^2−^	1962 (**1950**, *1942*)	1712 (**1663**, *1673*)	1974 (**1834**, *1944*)
[(Salen)Ru(NO)(HS)@Au_20_]	1894	1653	1859
[(bpb)Ru(NO)(HS)@Au_20_]	1882	1680	1826
[(Porph)Ru(NO)(HS)@Au_20_]	1910	1688	1894
[(Pc)Ru(NO)(HS)@Au_20_]	1921	1711	1903
[(Salen)Ru(NO)(HS)@Ag_20_]	1866	1636	1729
[(bpb)Ru(NO)(HS)@Ag_20_]	1866	1653	1793
[(Porph)Ru(NO)(HS)@Ag_20_]	1888	1692	1729
[(Pc)Ru(NO)(HS)@Ag_20_]	1886	1697	1427
[(Pc)Ru(NO)(HS)@Ag_10_Au_10_]	1910	1696	1833
[(Pc)Ru(NO)(HS)@Au_10_Ag_10_]	1901	1704	1677
[(Salen)Ru(NO)(HS)]	1844	1659	1826

^1^ Values obtained upon multiplying by the PBE0 correction factor (0.9518) [54]. ^2^ Experimental values are given in parentheses and in bold font. All data were obtained from Ref. [50]. ^3^ Values calculated at the B3LYP/DZVP level are given in parentheses and in italics. All data were obtained from Ref. [50].

**Table 3:** Spectroscopic and photophysical parameters governing the photo-rearrangement mechanism and optical addressability of the investigated Ru–NO systems and nanoparticle hybrids.

Species	*λ* (nm)/Σ*f*_i_ ^1^			Δ*E* (eV)		Δ*v*(N-O) (cm^−1^)		Δ*v*(Ru-N/O) (cm^−1^)		*S*	NRCI
	GS → GS*	MSII → MSII*	MSI → MSI*	(GS-MSII)	(MSII-MSI)	(GS-MSII)	(MSII-MSI)	(GS-MSII)	(MSII-MSI)		
[(CN)_5_Ru(NO)]^2−^	266/0.012	305/0.004	398/0.000	0.5	0.1	252	−278	−38	142	0.53	1.44
[(NH_3_)_5_Ru(NO)]^3+^	164/0.021	261/0.012	273/0.004	0.8	18.5	256	−242	−25	180	19.28	0.44
[(CN)_5_Fe(NO)]^2−^	271/0.003	286/0.001	352/0.007	0.8	0.4	262	−276	153	−11	1.15	1.09
**[(Salen)Ru(NO)(HS)@Au_20_]** ^2^	635/0.122	871/0.032	832/0.136	1.0	0.7	253	−216	−43	210	1.65	1.03
[(bpb)Ru(NO)(HS)@Au_20_]	612/0.025	695/0.024	1874/0.004	0.6	0.1	212	−152	−12	215	0.64	1.18
**[(Porph)Ru(NO)(HS)@Au_20_]** ^2^	682/0.100	753/0.016	1069/0.090	0.8	0.2	233	−216	−14	188	0.94	1.19
[(Pc)Ru(NO)(HS)@Au_20_]	852/0.131	1230/0.004	1874/0.004	0.7	0.2	220	−200	−18	192	0.84	1.17
**[(Salen)Ru(NO)(HS)@Ag_20_]** ^2^	1130/0.140	1749/0.033	1591/0.080	0.6	0.3	241	−98	−56	319	0.84	1.27
[(bpb)Ru(NO)(HS)@Ag_20_]	1054/0.003	667/0.139	2442/0.010	0.5	0.6	237	−161	−19	232	1.05	1.04
[(Porph)Ru(NO)(HS)@Ag_20_]	1287/0.111	1848/0.009	2862/0.001	0.8	0.2	206	−39	−2	271	0.95	0.94
[(Pc)Ru(NO)(HS)@Ag_20_]	1981/0.083	3244/0.043	3965/0.000	0.9	0.1	198	268	−22	105	0.94	1.14
[(Pc)Ru(NO)(HS)@Ag_10_Au_10_]	1242/0.044	2196/0.002	2974/0.001	0.7	0.3	226	−145	32	179	0.95	1.00
[(Pc)Ru(NO)(HS)@Au_10_Ag_10_]	1176/0.005	1696/0.002	1640/0.416	0.9	0.0	207	28	−27	316	0.87	1.14
[(Salen)Ru(NO)(HS)]	515/0.018	856/0.000	762/0.030	0.4	0.0	220	−176	−26	242	0.33	1.48

^1^ Σ*f*_i_ is the oscillator-strength sum within a ±0.20 eV bandwidth window. ^2^ Amongst the top three candidates predicted to exhibit the most favorable combination of optical brightness, stability, and controlled non-radiative relaxation.

**Table 4:** Calculated dynamic refractive indices, η(λ) and relative changes, Δη(λ) for [(Salen)Ru(NO)(HS)@Au_20_] and [Fe(CN)_5_NO]^2−^ at the TD-PBE0/def2-TZVP/PCM(Water) level of theory.

*λ* (nm)	*η*_GS_	*η*_MSII_	*η*_MSI_	η_GS_	Δ*η*_(MSII-GS)_	Δ*η*_(MSI-GS)_
[(Salen)Ru(NO)(HS)@Au_20_]						
400	1.46	1.49	1.50	+0.03	+0.04	0.04
532	1.48	1.52	1.53	+0.04	+0.05	0.05
635	1.49	1.54	1.55	+0.05	+0.06	0.06
800	1.50	1.54	1.55	+0.04	+0.05	0.05
1000	1.50	1.53	1.54	+0.03	+0.04	0.04
[Fe(CN)_5_NO]^2−^						
400	1.44	1.45	1.45	+0.01	+0.01	0.01
532	1.45	1.46	1.46	+0.01	+0.01	0.01
635	1.45	1.47	1.47	+0.02	+0.02	0.02
800	1.46	1.47	1.47	+0.01	+0.01	0.01
1000	1.46	1.47	1.47	+0.01	+0.01	0.01

## Data Availability

The original contributions presented in this study are included in the article/Supplementary Materials. Further inquiries can be directed to the corresponding author.

## Supplementary Materials

The following supporting information can be downloaded at: https://www.mdpi.com/article/10.3390/ijms262412113/s1.
