# Supplementary material for: Holography Meets Theranostics: DFT/TDDFT Insights into Ru–NO@M20 (M = Au, Ag) and Ru–NO@Au10Ag10(Pc) Nanohybrids as Phase-Switchable Molecular Devices
Source: Int J Mol Sci. 2025 Dec 16;26(24):12113. doi: 10.3390/ijms262412113 (PMC12733141; doi:10.3390/ijms262412113)
Supplement: Supplementary file 1 [file ijms-26-12113-s001.zip › ijms-4007686-supplementary.pdf]

Supplementary Material

Holography Meets Theranostics: Ru–NO@M_{20} Nanohybrids as Phase-Switchable Molecular Devices from DFT/TDDFT Insights

Niq Catevas and Athanassios Tsipis*

Holography Meets Theranostics: Ru–NO@M_{20} Nanohybrids as Phase-Switchable Molecular Devices from DFT/TDDFT Insights

Niq Catevas and Athanassios Tsipis *

Prof. Dr. A. Tsipis, PhD student N. Catevas
Department of Chemistry
University of Ioannina
T.Th.1186, Campus Ioannina University, Ioannina, Greece
E-mail: attsipis@uoi.gr

Supplementary Information

Contents

Figure S1. Optimized ground state, GS geometries : (a) [(bpb)Ru(NO)(HS)@Au_{20}], (b) [(Pc)Ru(NO)(HS)@Au_{20}], (c) [(Porph)Ru(NO)(HS)@Au_{20}] calculated at the PBE0/LANL2DZ(Ru)U6-31-G(d,p)/PCM(water) level.
Figure S2. Optimized ground state, GS geometries : (a) [(bpb)Ru(NO)(HS)@Ag_{20}], (b) [(Pc)Ru(NO)(HS)@Ag_{20}], (c) [(Porph)Ru(NO)(HS)@Ag_{20}] calculated at the PBE0/LANL2DZ(Ru)U6-31-G(d,p)/PCM(water) level.
Figure S3. Optimized ground state, MSII geometries : (a) [(bpb)Ru(NO)(HS)@Au_{20}], (b) [(Pc)Ru(NO)(HS)@Au_{20}], (c) [(Porph)Ru(NO)(HS)@Au_{20}] calculated at the PBE0/LANL2DZ(Ru)U6-31-G(d,p)/PCM(water) level.
Figure S4. Optimized ground state, MSII geometries : (a) [(bpb)Ru(NO)(HS)@Ag_{20}], (b) [(Pc)Ru(NO)(HS)@Ag_{20}], (c) [(Porph)Ru(NO)(HS)@Ag_{20}] calculated at the PBE0/LANL2DZ(Ru)U6-31-G(d,p)/PCM(water) level.
Figure S5. Optimized ground state, MSI geometries : (a) [(bpb)Ru(NO)(HS)@Au_{20}], (b) [(Pc)Ru(NO)(HS)@Au_{20}], (c) [(Porph)Ru(NO)(HS)@Au_{20}] calculated at the PBE0/LANL2DZ(Ru)U6-31-G(d,p)/PCM(water) level.
Figure S6. Optimized ground state, MSI geometries : (a) [(bpb)Ru(NO)(HS)@Ag_{20}], (b) [(Pc)Ru(NO)(HS)@Ag_{20}], (c) [(Porph)Ru(NO)(HS)@Ag_{20}] calculated at the PBE0/LANL2DZ(Ru)U6-31-G(d,p)/PCM(water) level.
Figure S7. Simulated IR spectra of GS state : (a) [(bpb)Ru(NO)(HS)@Au_{20}], (b) [(Pc)Ru(NO)(HS)@Au_{20}], (c) [(Porph)Ru(NO)(HS)@Au_{20}] calculated at the PBE0/LANL2DZ(Ru)U6-31-G(d,p)/PCM(water) level.
Figure S8. Simulated IR spectra of GS state: (a) [(bpb)Ru(NO)(HS)@Ag_{20}], (b) [(Pc)Ru(NO)(HS)@Ag_{20}], (c) [(Porph)Ru(NO)(HS)@Ag_{20}] calculated at the PBE0/LANL2DZ(Ru)U6-31-G(d,p)/PCM(water) level.
Figure S9. Simulated IR spectra of MSII state: (a) [(bpb)Ru(NO)(HS)@Au_{20}], (b) [(Pc)Ru(NO)(HS)@Au_{20}], (c) [(Porph)Ru(NO)(HS)@Au_{20}] calculated at the PBE0/LANL2DZ(Ru)U6-31-G(d,p)/PCM(water) level.
Figure S10. Simulated IR spectra of MSII state: (a) [(bpb)Ru(NO)(HS)@Ag_{20}], (b) [(Pc)Ru(NO)(HS)@Ag_{20}], (c) [(Porph)Ru(NO)(HS)@Ag_{20}] calculated at the PBE0/LANL2DZ(Ru)U6-31-G(d,p)/PCM(water) level.
Figure S11. Simulated IR spectra of MSI state: (a) [(bpb)Ru(NO)(HS)@Au_{20}], (b) [(Pc)Ru(NO)(HS)@Au_{20}], (c) [(Porph)Ru(NO)(HS)@Au_{20}] calculated at the PBE0/LANL2DZ(Ru)U6-31-G(d,p)/PCM(water) level.
Figure S12. Simulated IR spectra of MSI state: (a) [(bpb)Ru(NO)(HS)@Ag_{20}], (b) [(Pc)Ru(NO)(HS)@Ag_{20}], (c) [(Porph)Ru(NO)(HS)@Ag_{20}] calculated at the PBE0/LANL2DZ(Ru)U6-31-G(d,p)/PCM(water) level.
Figure S13. Simulated UV-Vis spectra of GS state : (a) [(bpb)Ru(NO)(HS)@Au_{20}], (b) [(Pc)Ru(NO)(HS)@Au_{20}], (c) [(Porph)Ru(NO)(HS)@Au_{20}] calculated at the PBE0/LANL2DZ(Ru)U6-31-G(d,p)/PCM(water) level.
Figure S14. Simulated UV-Vis spectra of GS state: (a) [(bpb)Ru(NO)(HS)@Ag_{20}], (b) [(Pc)Ru(NO)(HS)@Ag_{20}], (c) [(Porph)Ru(NO)(HS)@Ag_{20}] calculated at the PBE0/LANL2DZ(Ru)U6-31-G(d,p)/PCM(water) level.
Figure S15. Simulated UV-Vis spectra of MSII state: (a) [(bpb)Ru(NO)(HS)@Au_{20}], (b) [(Pc)Ru(NO)(HS)@Au_{20}], (c) [(Porph)Ru(NO)(HS)@Au_{20}] calculated at the PBE0/LANL2DZ(Ru)U6-31-G(d,p)/PCM(water) level.

Figure S16. Simulated UV-Vis spectra of MSII state: (a) [(bpb)Ru(NO)(HS)@Ag_{20}], (b) [(Pc)Ru(NO)(HS)@Ag_{20}], (c) [(Porph)Ru(NO)(HS)@Ag_{20}] calculated at the PBE0/LANL2DZ(Ru)U6-31-G(d,p)/PCM(water) level.
Figure S17. Simulated UV-Vis spectra of MSI state: (a) [(bpb)Ru(NO)(HS)@Au_{20}], (b) [(Pc)Ru(NO)(HS)@Au_{20}], (c) [(Porph)Ru(NO)(HS)@Au_{20}] calculated at the PBE0/LANL2DZ(Ru)U6-31-G(d,p)/PCM(water) level.
Figure S18. Simulated UV-Vis spectra of MSI state: (a) [(bpb)Ru(NO)(HS)@Ag_{20}], (b) [(Pc)Ru(NO)(HS)@Ag_{20}], (c) [(Porph)Ru(NO)(HS)@Ag_{20}] calculated at the PBE0/LANL2DZ(Ru)U6-31-G(d,p)/PCM(water) level.
Table S1. Comparisons of selected structural parameters of the model complexes used for benchmarking the PBE0/LANL2DZ(Ru)/6-31G(d,p)/PCM(water) computational protocol.
Table S2. Cartesian coordinates and energetic data.

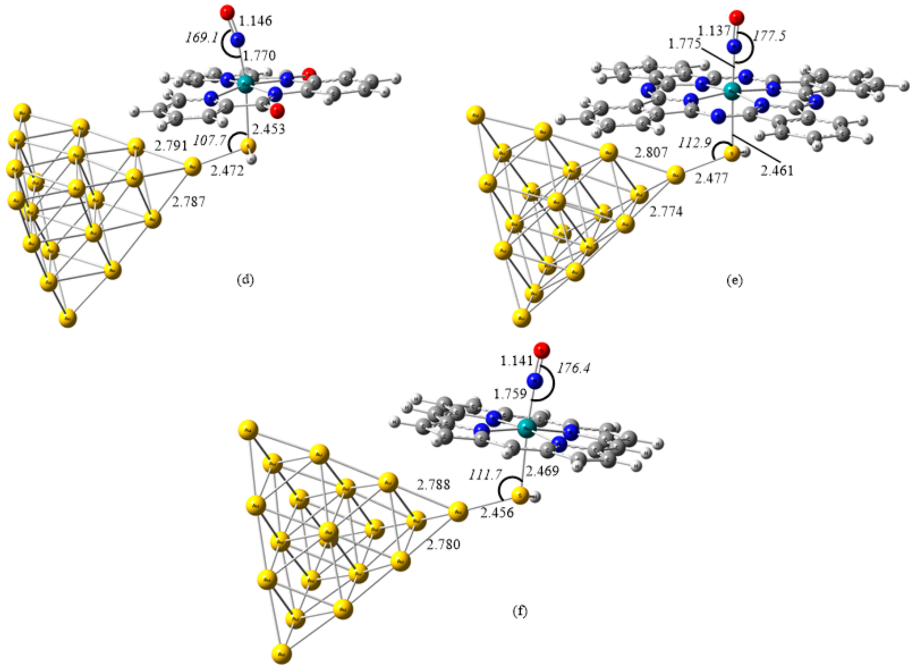

Figure S1. Optimized ground state, GS geometries : (a) [(bpb)Ru(NO)(HS)@Au_{20}], (b) [(Pc)Ru(NO)(HS)@Au_{20}], (c) [(Porph)Ru(NO)(HS)@Au_{20}] calculated at the PBE0/LANL2DZ(Ru)U6-31-G(d,p)/PCM(water) level.

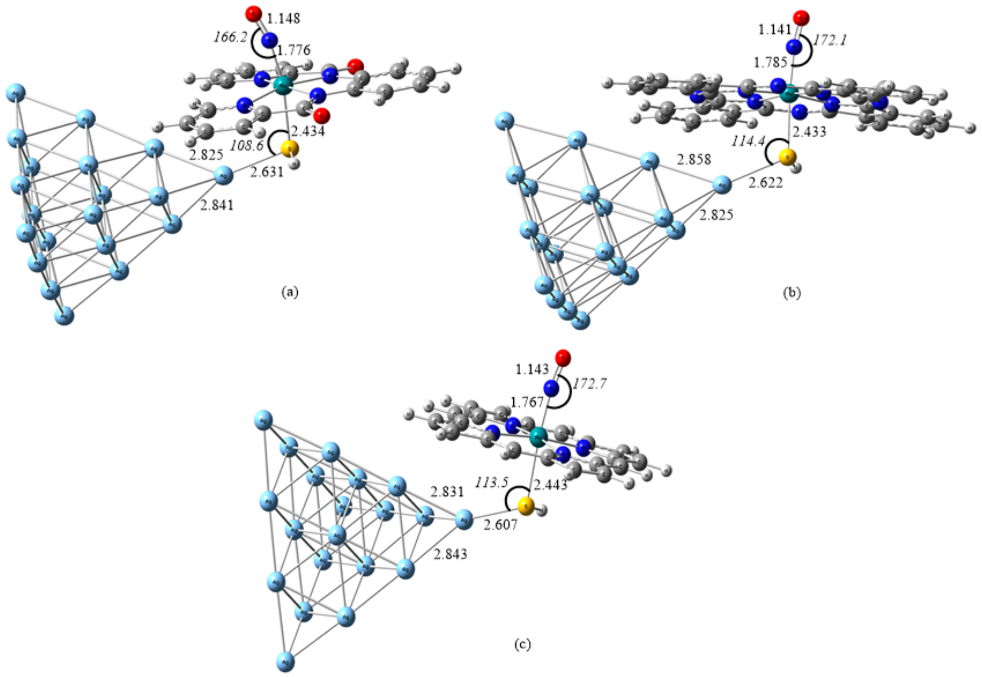

Figure S2. Optimized ground state, GS geometries : (a) [(bpb)Ru(NO)(HS)@Ag_{20}], (b) [(Pc)Ru(NO)(HS)@Ag_{20}], (c) [(Porph)Ru(NO)(HS)@Ag_{20}] calculated at the PBE0/LANL2DZ(Ru)U6-31-G(d,p)/PCM(water) level.

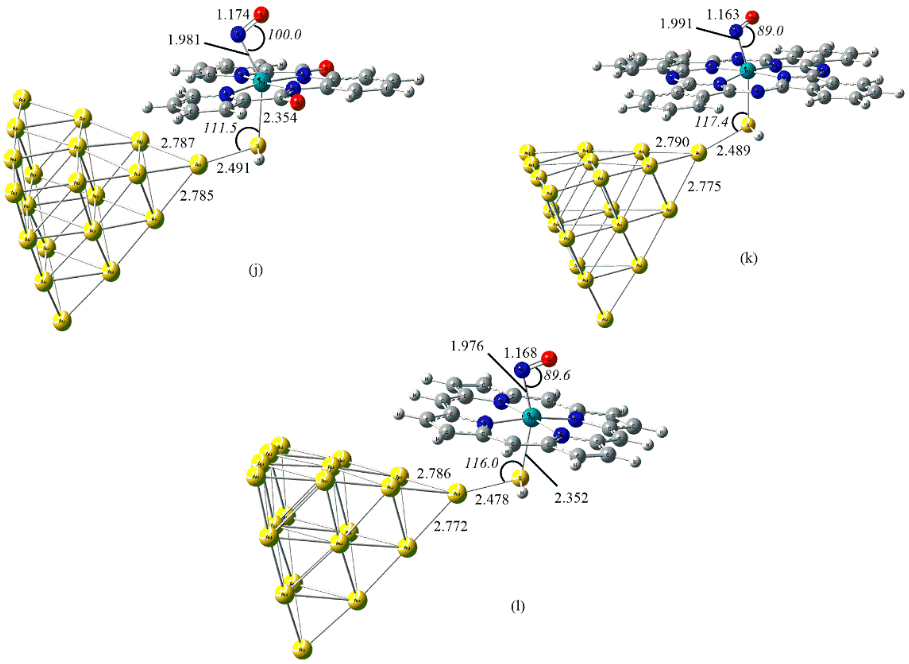

Figure S3. Optimized ground state, MSII geometries : (a) [(bpb)Ru(NO)(HS)@Au_{20}], (b) [(Pc)Ru(NO)(HS)@Au_{20}], (c) [(Porph)Ru(NO)(HS)@Au_{20}] calculated at the PBE0/LANL2DZ(Ru)U6-31-G(d,p)/PCM(water) level.

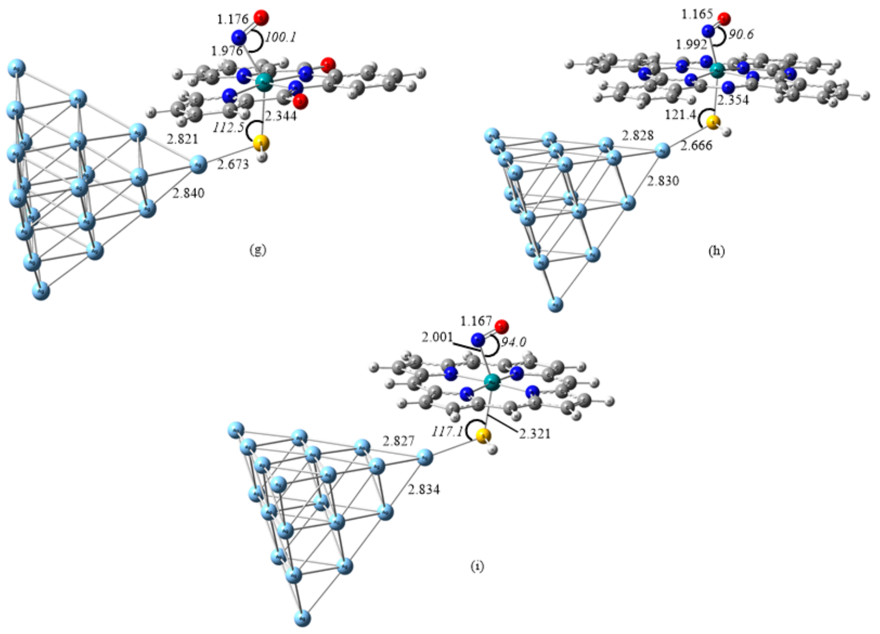

Figure S4. Optimized ground state, MSII geometries : (a) [(bpb)Ru(NO)(HS)@Ag_{20}], (b) [(Pc)Ru(NO)(HS)@Ag_{20}], (c) [(Porph)Ru(NO)(HS)@Ag_{20}] calculated at the PBE0/LANL2DZ(Ru)U6-31-G(d,p)/PCM(water) level.

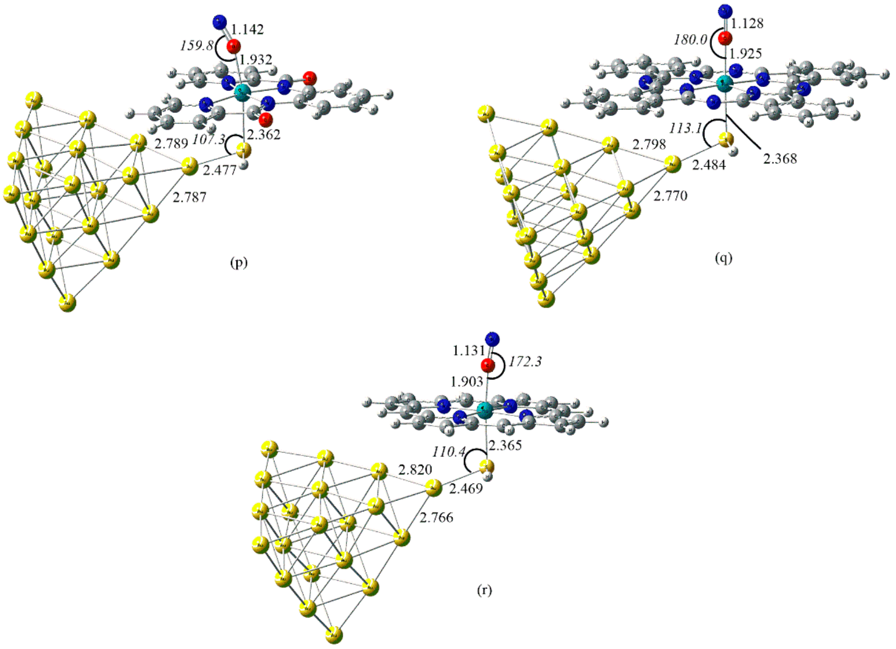

Figure S5. Optimized ground state, MSI geometries : (a) [(bpb)Ru(NO)(HS)@Au_{20}], (b) [(Pc)Ru(NO)(HS)@Au_{20}], (c) [(Porph)Ru(NO)(HS)@Au_{20}] calculated at the PBE0/LANL2DZ(Ru)U6-31-G(d,p)/PCM(water) level.

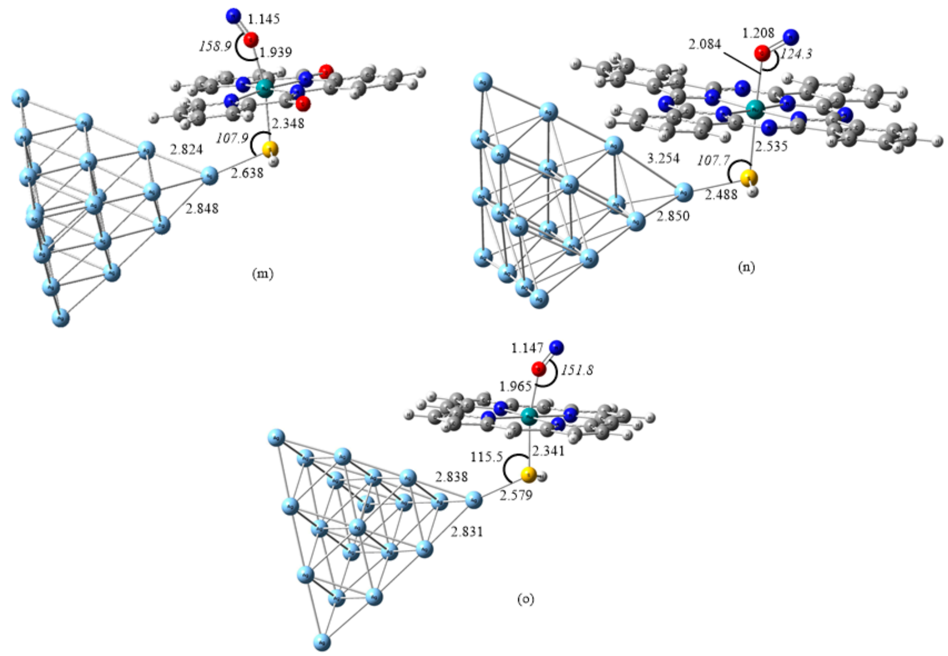

Figure S6. Optimized ground state, MSI geometries : (a) [(bpb)Ru(NO)(HS)@Ag_{20}], (b) [(Pc)Ru(NO)(HS)@Ag_{20}], (c) [(Porph)Ru(NO)(HS)@Ag_{20}] calculated at the PBE0/LANL2DZ(Ru)U6-31-G(d,p)/PCM(water) level.

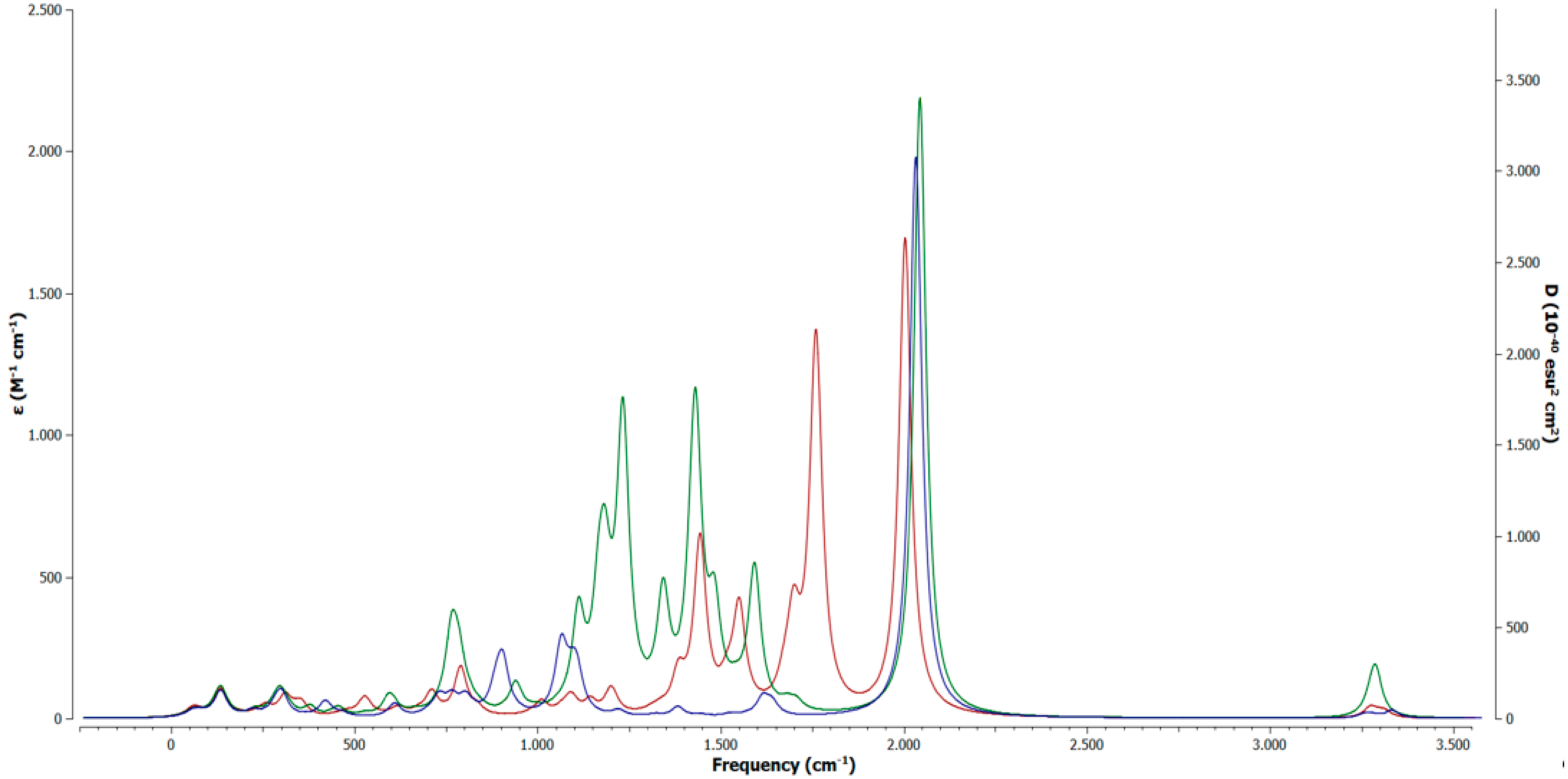

Figure S7. Simulated IR spectra of (a) [(bpb)Ru(NO)(HS)@Au_{20}] (red line), (b) [(Pc)Ru(NO)(HS)@Au_{20}], (green line) (c) [(Porph)Ru(NO)(HS)@Au_{20}] (blue line) in their GS states, calculated at the PBE0/LANL2DZ(Ru)U6-31-G(d,p)/PCM(water) level.

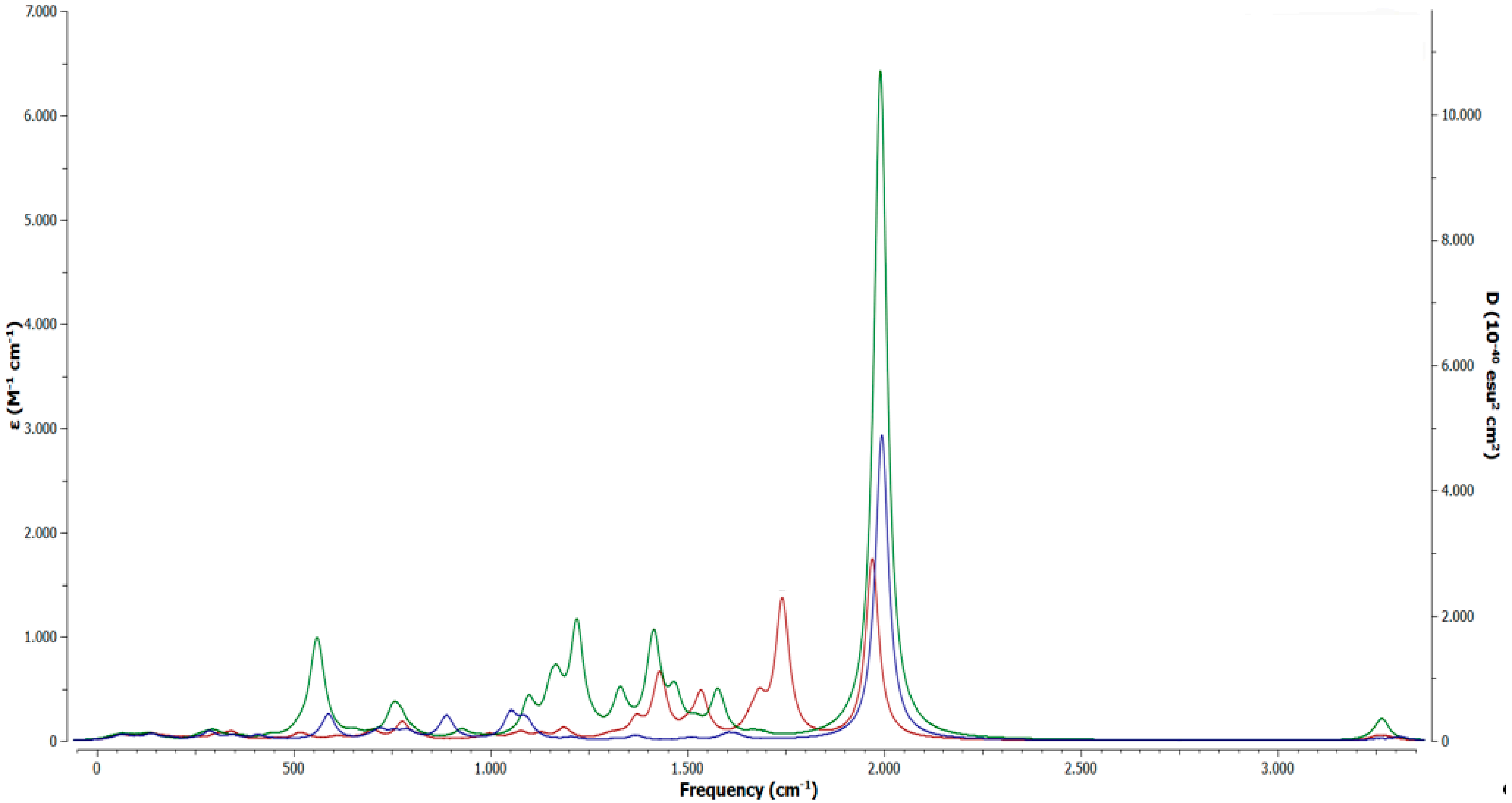

Figure S8. Simulated IR spectra of (a) [(bpb)Ru(NO)(HS)@Ag_{20}] (red line), (b) [(Pc)Ru(NO)(HS)@Ag_{20}], (green line) (c) [(Porph)Ru(NO)(HS)@Ag_{20}] (blue line) in their GS states, calculated at the PBE0/LANL2DZ(Ru)U6-31-G(d,p)/PCM(water) level.

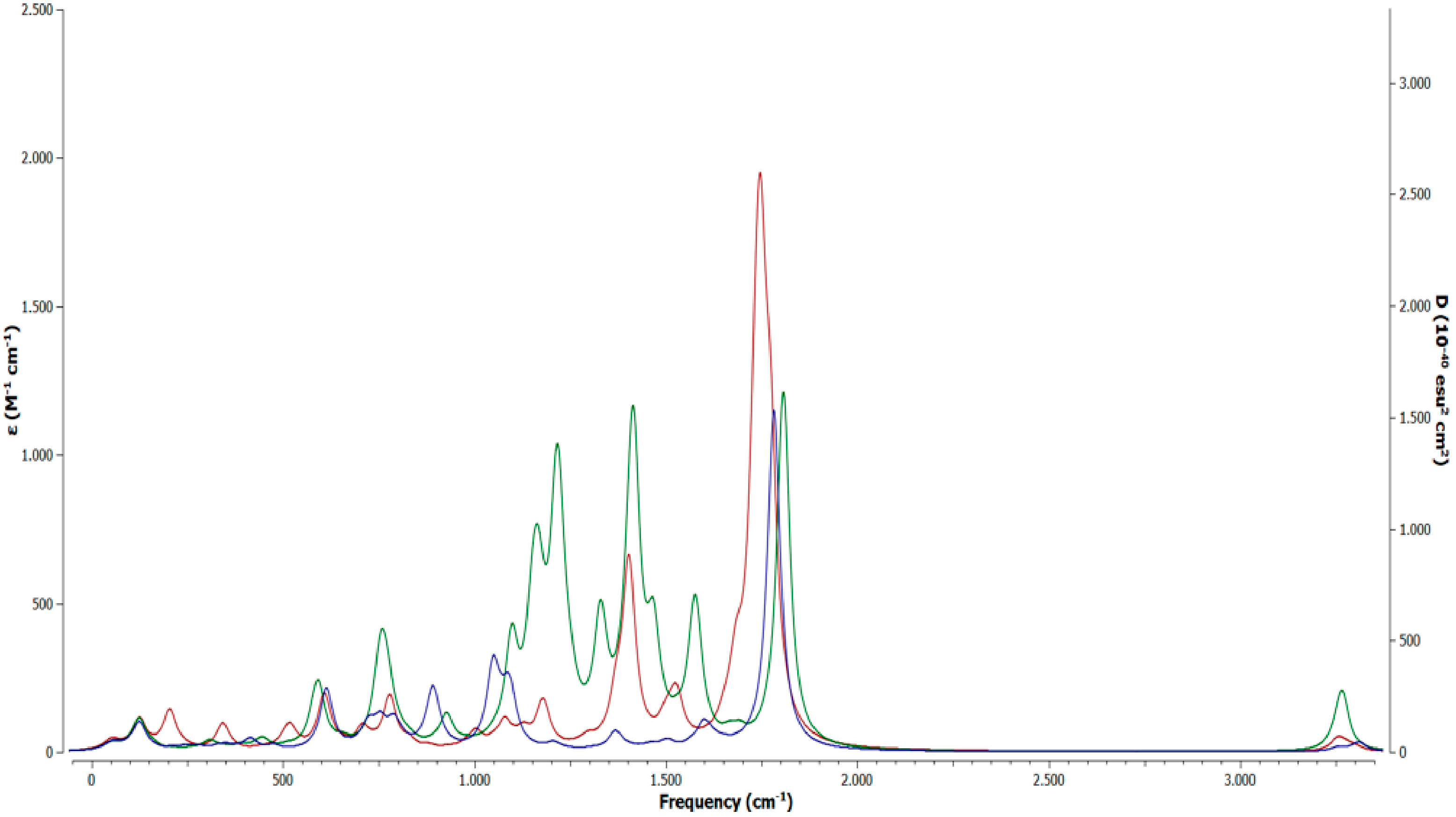

Figure S9. Simulated IR spectra of (a) [(bpb)Ru(NO)(HS)@Au20] (red line), (b) [(Pc)Ru(NO)(HS)@Au20], (green line) (c) [(Porph)Ru(NO)(HS)@Au20] (blue line) in their MSII states, calculated at the PBE0/LANL2DZ(Ru)U6-31-G(d,p)/PCM(water) level.

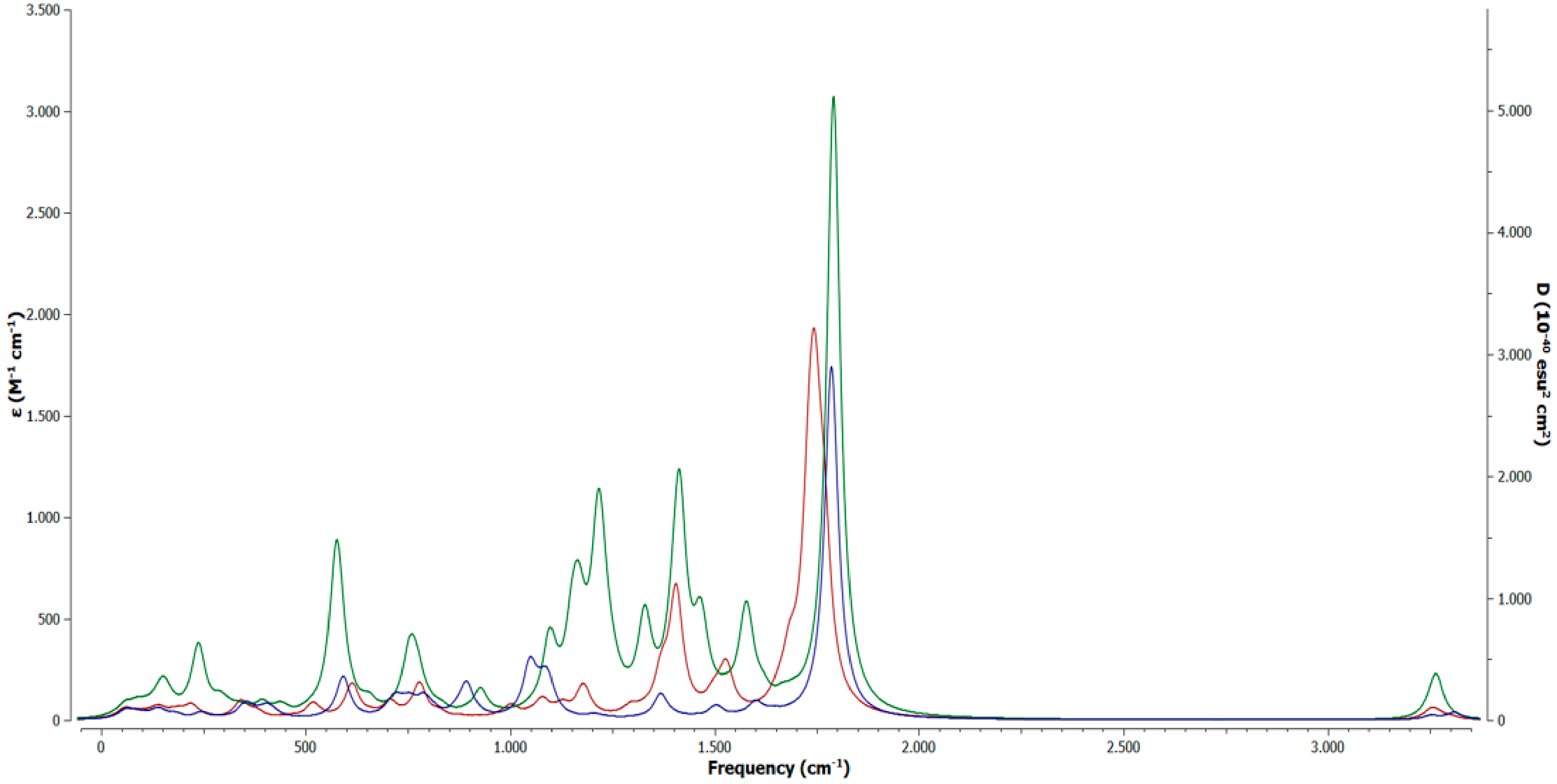

Figure S10. Simulated IR spectra of (a) [(bpb)Ru(NO)(HS)@Ag_{20}] (red line), (b) [(Pc)Ru(NO)(HS)@Ag_{20}], (green line) (c) [(Porph)Ru(NO)(HS)@Ag_{20}] (blue line) in their MSII states, calculated at the PBE0/LANL2DZ(Ru)U6-31-G(d,p)/PCM(water) level.

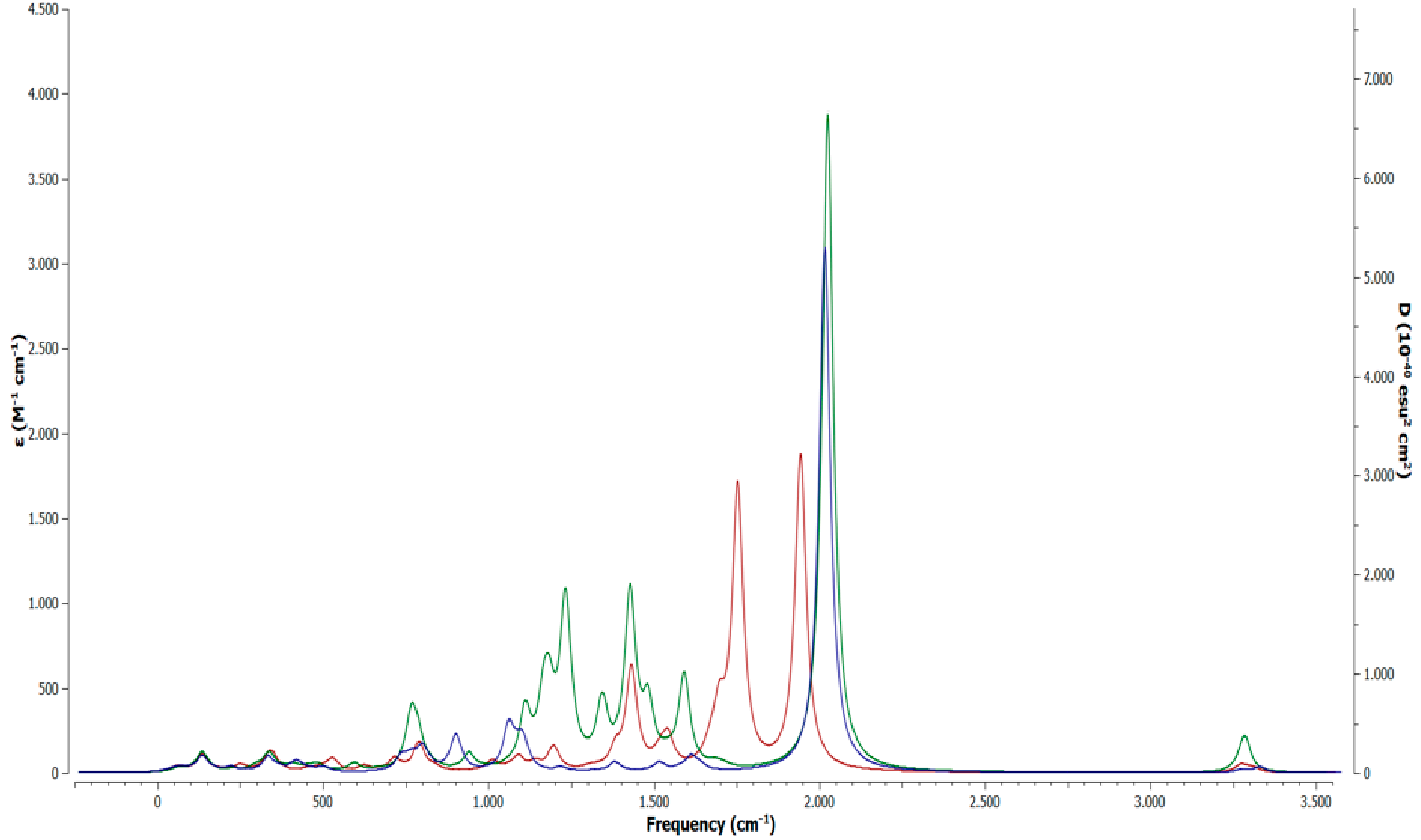

Figure S11. Simulated IR spectra of (a) [(bpb)Ru(NO)(HS)@Au20] (red line), (b) [(Pc)Ru(NO)(HS)@Au20], (green line) (c) [(Porph)Ru(NO)(HS)@Au20] (blue line) in their MSI states, calculated at the PBE0/LANL2DZ(Ru)U6-31-G(d,p)/PCM(water) level.

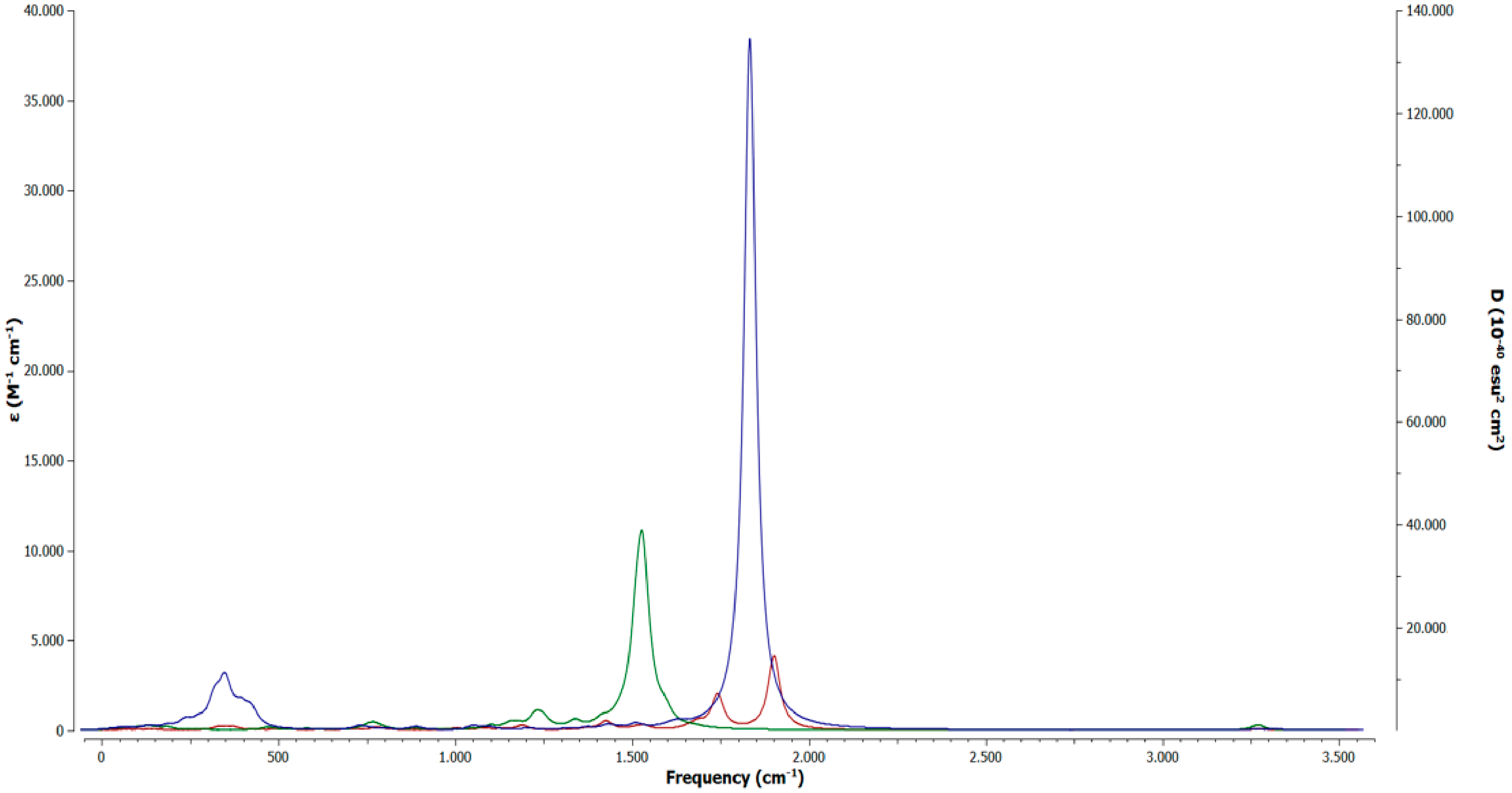

Figure S12. Simulated IR spectra of (a) [(bpb)Ru(NO)(HS)@Ag_{20}] (red line), (b) [(Pc)Ru(NO)(HS)@Ag_{20}], (green line) (c) [(Porph)Ru(NO)(HS)@Ag_{20}] (blue line) in their MSI states, calculated at the PBE0/LANL2DZ(Ru)U6-31-G(d,p)/PCM(water) level.

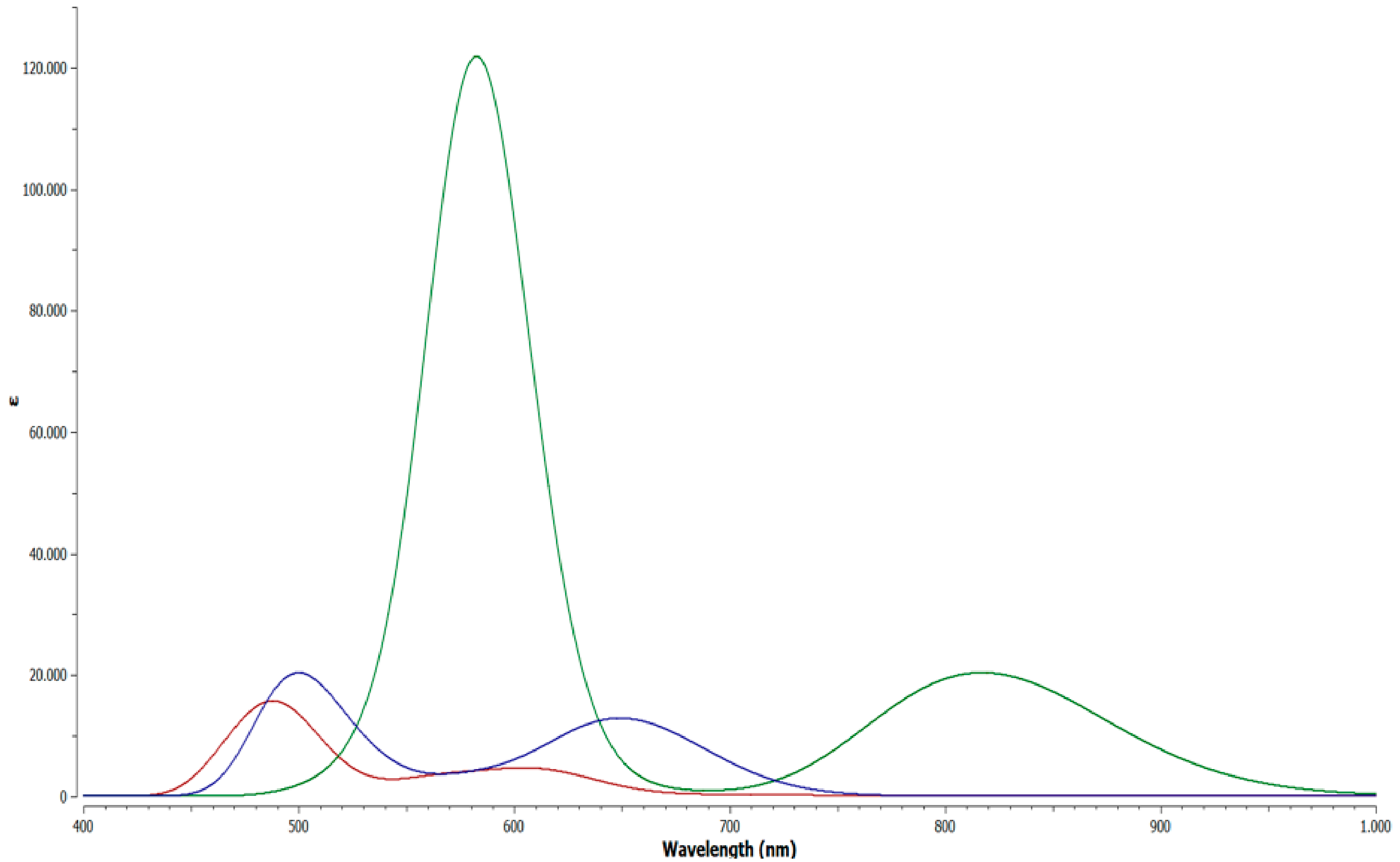

Figure S13. Simulated UV-Vis spectra of (a) [(bpb)Ru(NO)(HS)@Au_{20}] (red line), (b) [(Pc)Ru(NO)(HS)@Au_{20}], (green line) (c) [(Porph)Ru(NO)(HS)@Au_{20}] (blue line) in their GS states, calculated at the PBE0/LANL2DZ(Ru)U6-31-G(d,p)/PCM(water) level.

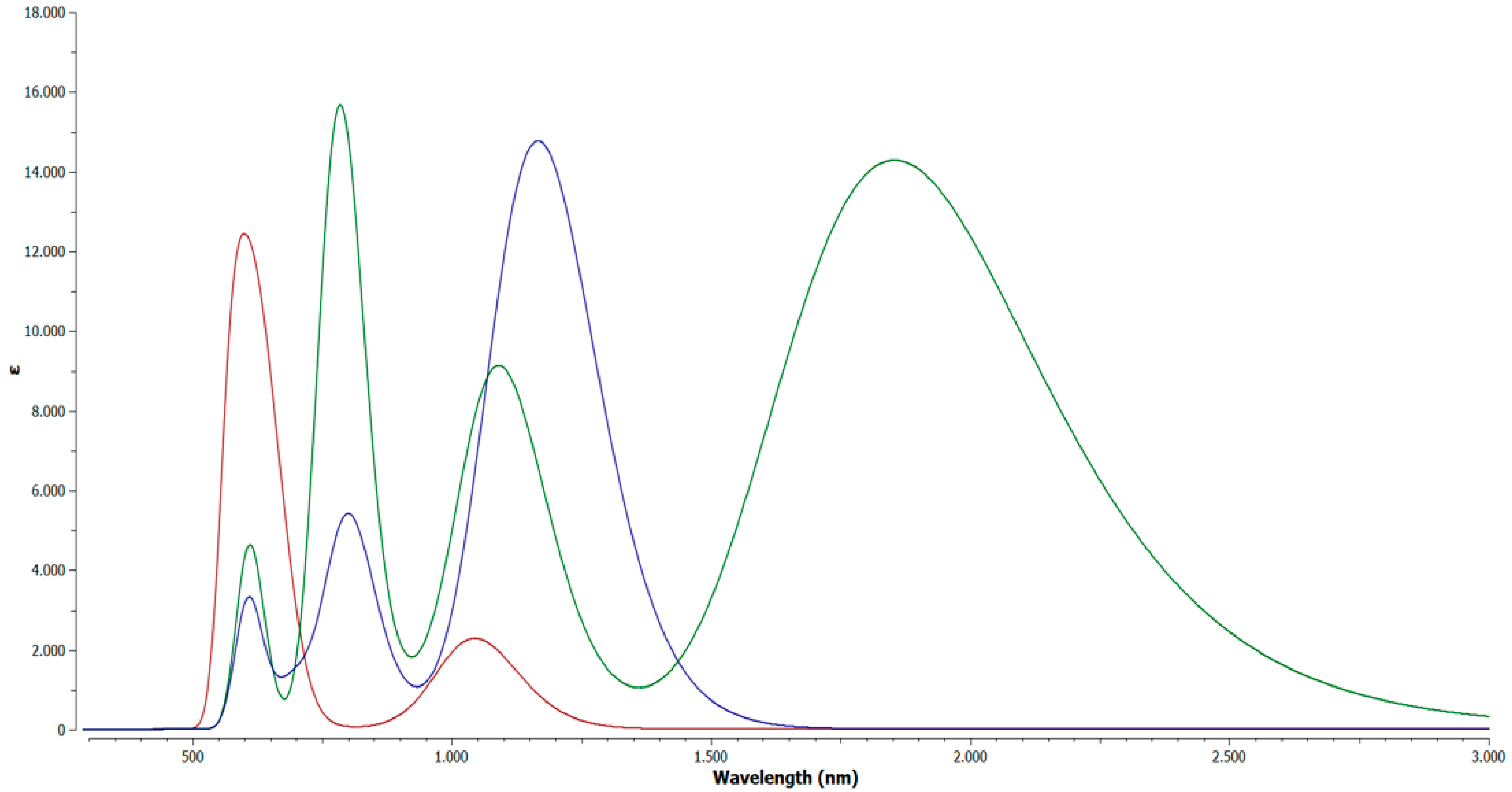

Figure S14. Simulated UV-Vis spectra of (a) [(bpb)Ru(NO)(HS)@Ag_{20}] (red line), (b) [(Pc)Ru(NO)(HS)@Ag_{20}], (green line) (c) [(Porph)Ru(NO)(HS)@Ag_{20}] (blue line) in their GS states, calculated at the PBE0/LANL2DZ(Ru)U6-31-G(d,p)/PCM(water) level.

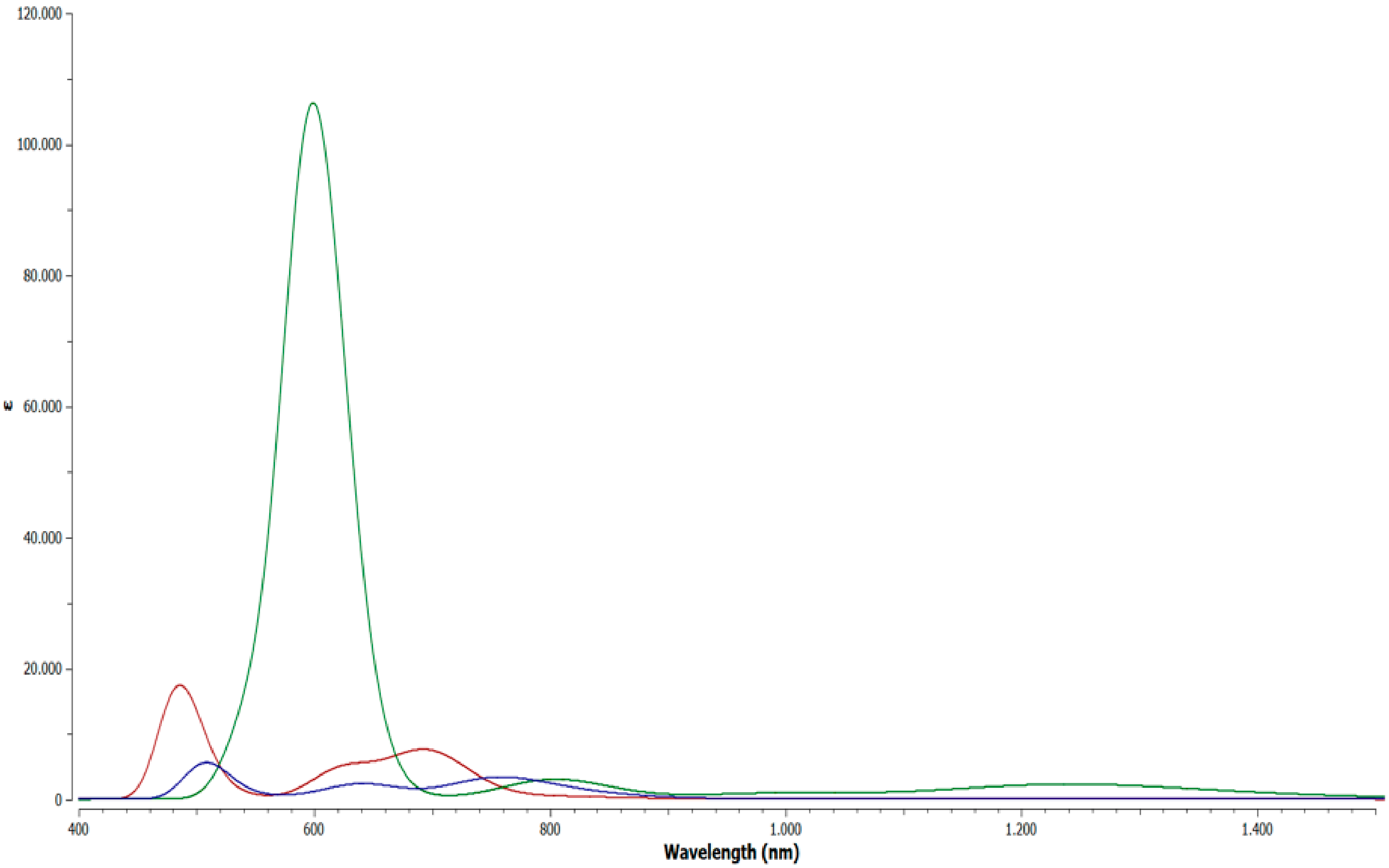

Figure S15. Simulated UV-Vis spectra of (a) [(bpb)Ru(NO)(HS)@Au_{20}] (red line), (b) [(Pc)Ru(NO)(HS)@Au_{20}], (green line) (c) [(Porph)Ru(NO)(HS)@Au_{20}] (blue line) in their MSII states, calculated at the PBE0/LANL2DZ(Ru)U6-31-G(d,p)/PCM(water) level.

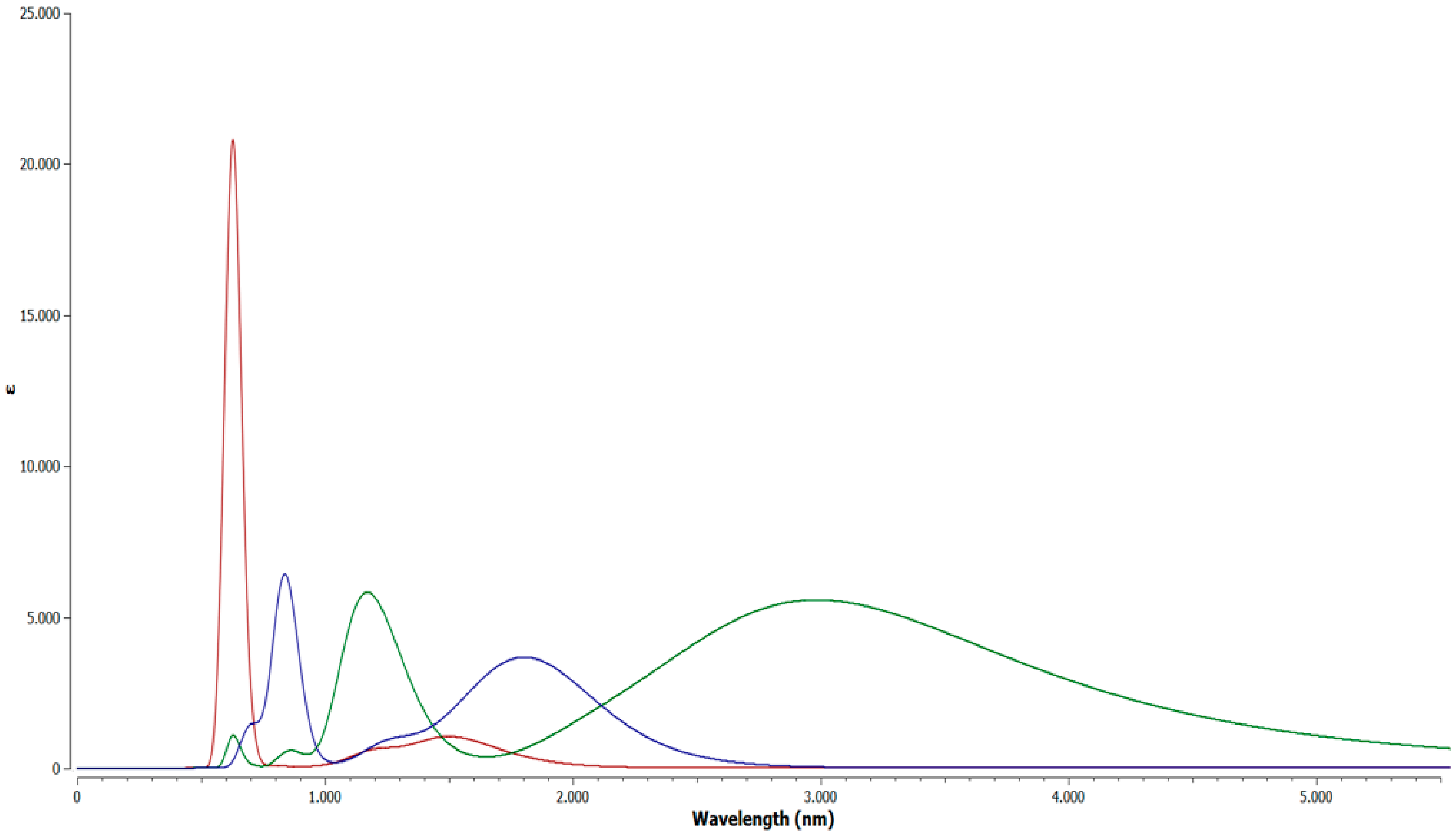

Figure S16. Simulated UV-Vis spectra of (a) [(bpb)Ru(NO)(HS)@Ag_{20}] (red line), (b) [(Pc)Ru(NO)(HS)@Ag_{20}], (green line) (c) [(Porph)Ru(NO)(HS)@Ag_{20}] (blue line) in their MSII states, calculated at the PBE0/LANL2DZ(Ru)U6-31-G(d,p)/PCM(water) level.

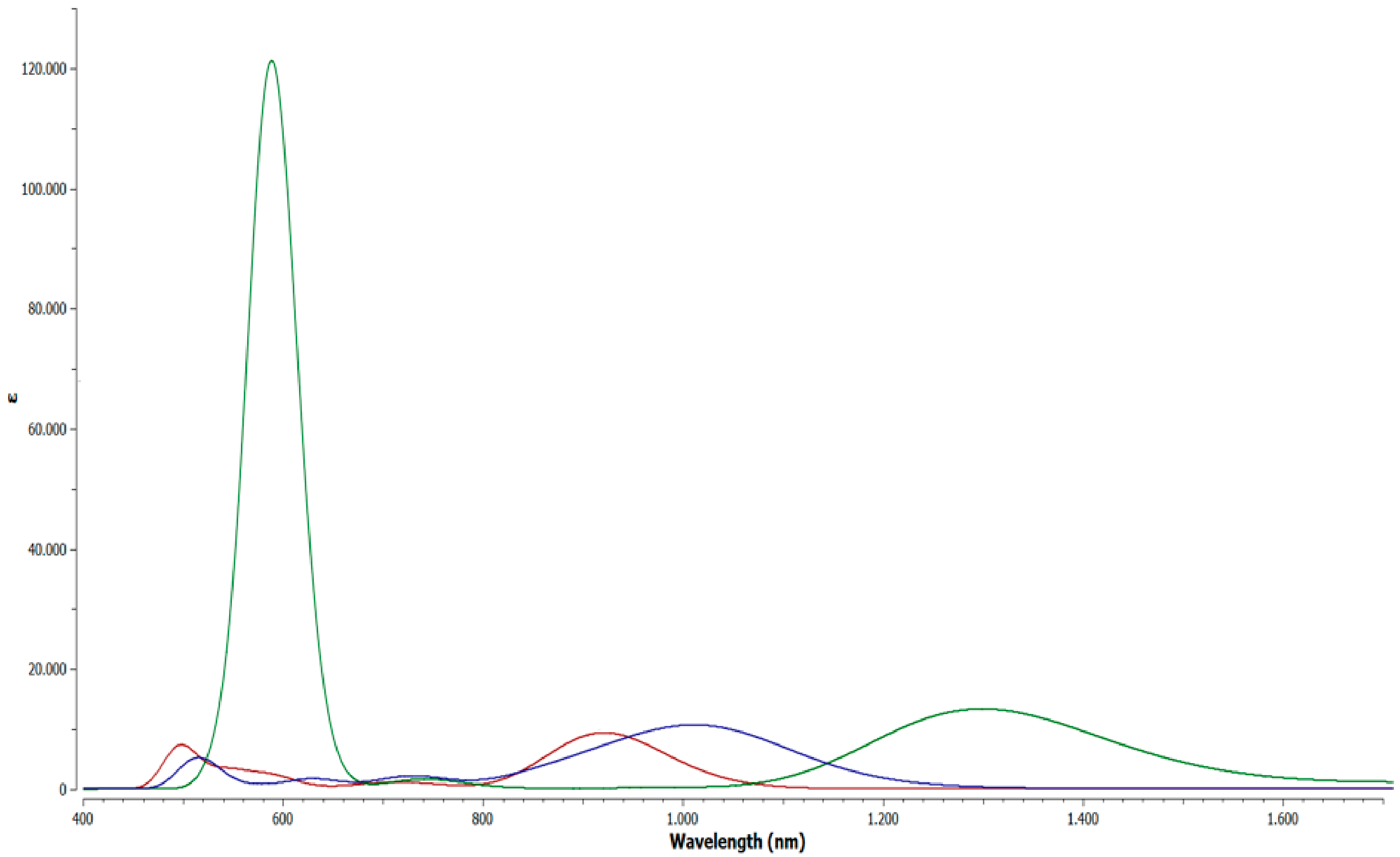

Figure S17. Simulated UV-Vis spectra of (a) [(bpb)Ru(NO)(HS)@Au_{20}] (red line), (b) [(Pc)Ru(NO)(HS)@Au_{20}], (green line) (c) [(Porph)Ru(NO)(HS)@Au_{20}] (blue line) in their MSI states, calculated at the PBE0/LANL2DZ(Ru)U6-31-G(d,p)/PCM(water) level.

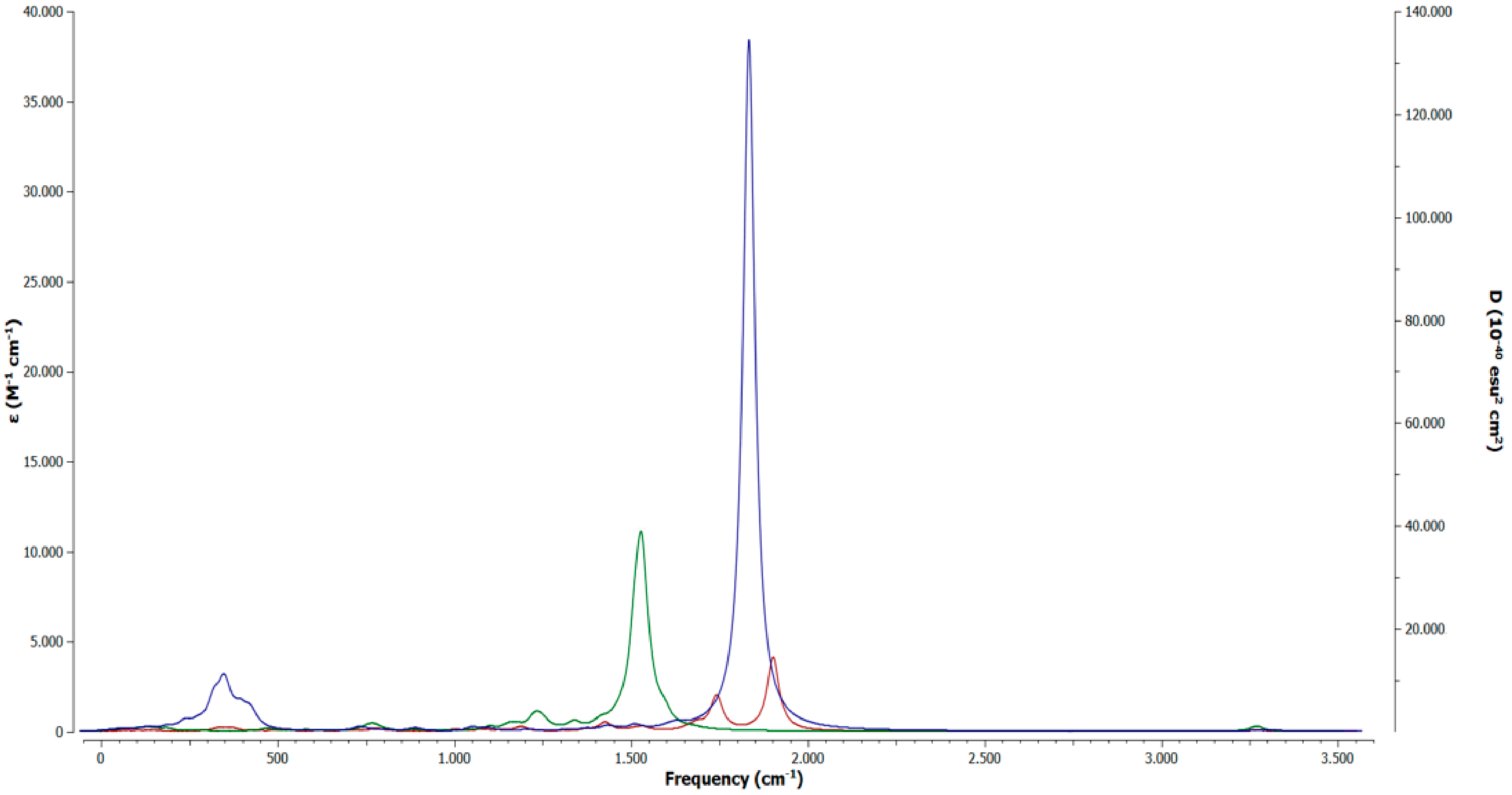

Figure S18. Simulated UV-Vis spectra of (a) [(bpb)Ru(NO)(HS)@Ag_{20}] (red line), (b) [(Pc)Ru(NO)(HS)@Ag_{20}], (green line) (c) [(Porph)Ru(NO)(HS)@Ag_{20}] (blue line) in their MSI states, calculated at the PBE0/LANL2DZ(Ru)U6-31-G(d,p)/PCM(water) level.

Table S1. Comparisons of selected structural parameters of the model complexes used for benchmarking the PBE0/LANL2DZ(Ru)/6-31G(d,p)/PCM(water) computational protocol.[1]

Str. Par.	$[(CN)_5Ru(NO)]^{2-}$,			$[(NH_3)_5Ru(NO)]^{3+}$			$[(CN)_5Fe(NO)]^{2-}$		
	Our Method	Exptl.[1]	Other DFT[2]	Our Method	Exptl.[1]	Other DFT[2]	Our Method	Exptl.[1]	Other DFT[2]
R_e(N-O)	1.138 (1.127)[3]	1.130	1.166	1.123	1.172	1.129	1.135 (1.123)	1.133	1.162
R_e(Ru-NO)	1.785 (1.778)	1.773	1.786	1.776	1.770	1.819	1.620 (1.624)	1.666	1.630
R_e(Ru-L)$_{trans}$	2.056 (2.066)	2.051	2.099	2.128	2.020	2.194	1.920 (1.931)	1.926	1.958
R_e(Ru-L)$_{cis}$	2.057 (2.066)	2.064	2.113	2.136	2.100	2.211	1.913 (1.927)	1.931	1.965
<Ru-N-O	180.0 (180.0)	174.4	180.0	178.8	172.8	179.3	180.0 (180.0)	179.8	180.0

[1] Data taken from ref. [49].

[2] Calculated using the B3LYP/DZVP method [49].

[3] Numbers in parenthesis were obtained using the higher quality PBE0/LANL2TZ(M)U6-311G(2d,2p)/PCM(Water) method.

Table S2. Cartesian coordinates and energetic data.

[(bpb)Ru(NO)(HS)@Au$_{20}$] GS

Ru	-7.37943800	-0.01691600	0.53269500
O	-9.19951900	-3.45951300	-0.77535100
O	-8.76787800	3.67329600	-0.62743200
O	-8.09789800	0.05752600	3.34521700
N	-8.73812100	-1.24666100	-0.25572600
N	-8.55226600	1.39473000	-0.25040700
N	-6.41363600	-1.91338200	0.83881100
N	-6.18110000	1.73854900	0.90046500
C	-9.82350800	-0.55586900	-0.81713900
C	-9.72583400	0.86504300	-0.81183500
C	-10.76210200	1.63015300	-1.35583000
C	-11.88126600	1.00169300	-1.89643300
C	-11.97641500	-0.38719400	-1.90271700
C	-10.95421100	-1.16719700	-1.36767400
C	-8.49147000	-2.57226800	-0.29594600
C	-7.16849800	-2.91730300	0.34152700
C	-6.74585100	-4.23759800	0.40111600
C	-5.52132900	-4.53088900	0.99249000
C	-4.75609400	-3.49066200	1.50869100
C	-5.23894100	-2.19028500	1.41172600
C	-8.16321000	2.68635500	-0.20456400
C	-6.81329700	2.84483500	0.45117700
C	-6.24417400	4.10455100	0.57247600
C	-4.99772500	4.22936400	1.17743700
C	-4.35627200	3.08523600	1.63889100
C	-4.98265600	1.85309700	1.47866100
Au	-3.81892200	-0.11241600	-1.23716900
Au	-1.77754000	1.53832900	-0.30363500
Au	-1.26126500	-0.48630300	-2.27854800
Au	-1.88289600	-1.25320700	0.41825900
Au	0.71740700	-1.73320500	-0.63340900
Au	0.83517300	1.24767000	-1.40328600
Au	0.13628000	0.43729900	1.50341100
Au	0.22544200	3.13650800	0.63201200
Au	1.26832500	-0.84641500	-3.26815100
Au	0.00125200	-2.34782600	2.05662900
Au	3.00954800	0.04496600	0.43084500
Au	2.34366000	1.98825700	2.39384700
Au	3.00923700	2.76820300	-0.38751600
Au	2.23491700	-0.63773800	3.08121000
Au	3.51477800	0.85408000	-2.24855800
Au	2.78190300	-2.72195100	1.04561100
Au	3.40597500	-2.00651500	-1.49941500
Au	2.37679100	4.60387500	1.55699700
Au	2.05442100	-3.32494000	3.62579200
Au	3.90897100	-1.16926600	-4.06154200
H	-10.67068900	2.70839500	-1.34768100
H	-12.68084900	1.60652200	-2.31395200
H	-12.85075900	-0.87350600	-2.32522600
H	-11.01067900	-2.24787600	-1.36965100
H	-7.39106100	-5.00076600	-0.01873600

H	-5.17062200	-5.55610900	1.05219300
H	-3.79490600	-3.66619400	1.97987800
H	-4.67353600	-1.35188400	1.80132300
H	-6.79742600	4.95498600	0.19080400
H	-4.53440200	5.20452700	1.28789900
H	-3.38338000	3.13001100	2.11631100
H	-4.51223800	0.93768300	1.81785600
N	-7.94400000	0.02334300	2.21015900
S	-6.25650400	-0.14205600	-1.64477200
H	-6.37987700	1.14578500	-2.01556700

Sum of electronic and zero-point Energies= -4393.058163
Sum of electronic and thermal Energies= -4392.988324
Sum of electronic and thermal Enthalpies= -4392.987380
Sum of electronic and thermal Free Energies= -4393.194308

[(Pc)Ru(NO)(HS)@Au$_{20}$] GS

Ru	7.20637100	-0.00226100	0.65710000
O	8.84312400	0.06400300	3.06425500
C	4.33938200	4.56684800	2.03902700
C	3.14320800	4.78902000	2.71592400
C	2.38459600	3.72167900	3.22270300
C	2.80037600	2.40160900	3.06696800
C	3.99787700	2.17743600	2.39169600
C	4.70663500	0.95643500	2.03499400
N	4.27225800	-0.26859300	2.30642300
C	4.86211000	-1.39672300	1.93122100
C	4.32576200	-2.72750500	2.17976200
C	3.17073500	-3.16602800	2.82363400
C	2.93843900	-4.53844500	2.86754200
C	3.83410500	-5.44834700	2.28294300
C	4.98686200	-5.01199000	1.63667800
C	5.22208700	-3.64061700	1.59218600
C	6.29366600	-2.85495900	0.99717100
N	6.02636800	-1.53836800	1.23763700
N	7.31565700	-3.36849700	0.32925400
C	8.27424300	-2.66954100	-0.26120100
C	9.35713800	-3.25271900	-1.04115600
C	9.68734400	-4.57129000	-1.33924200
C	10.80832000	-4.79106500	-2.13465100
C	11.57629300	-3.72205500	-2.62221300
C	11.24532700	-2.40233900	-2.32860900
C	10.12729800	-2.18051400	-1.53024100
C	9.50230900	-0.95840700	-1.04342900
N	8.42502100	-1.31552400	-0.28724200
N	9.92722200	0.26322000	-1.32873000
C	9.34351200	1.38935000	-0.94617600
C	9.80487400	2.72077600	-1.31201100
C	10.88761300	3.15669000	-2.07036100
C	11.04682500	4.52902400	-2.23746700
C	10.14646500	5.44177500	-1.66530500
C	9.06111300	5.00839300	-0.90972900
C	8.90179200	3.63628900	-0.73830200
C	7.90189600	2.85125400	-0.02902300

N	8.22399700	1.53412200	-0.17999500
N	6.86295100	3.36384600	0.61433300
C	5.92221100	2.66503000	1.23199700
N	5.83678700	1.30945700	1.36266900
C	4.75789000	3.24823800	1.88354800
H	4.92765600	5.38944800	1.64513400
H	2.78913100	5.80561100	2.85785700
H	1.45679700	3.93164000	3.74688200
H	2.20951900	1.57497600	3.45093500
H	2.47429400	-2.46038300	3.26792000
H	2.04818500	-4.91434700	3.36309900
H	3.62041000	-6.51147100	2.33726600
H	5.67948400	-5.71200400	1.18050900
H	9.09004200	-5.39560800	-0.96297300
H	11.09577800	-5.80767500	-2.38523600
H	12.44371200	-3.93146300	-3.24078000
H	11.83541600	-1.57351400	-2.70636700
H	11.58056000	2.44842700	-2.51311800
H	11.88183900	4.90388400	-2.82149900
H	10.30193600	6.50550600	-1.81759100
H	8.36239400	5.71148200	-0.46780500
Au	3.49184200	-0.12305000	-1.11177200
Au	1.06504000	-1.31773900	-1.72847300
Au	1.22994800	1.50395700	-1.21028600
Au	1.61071500	-0.39283300	0.95411700
Au	-0.66336800	1.32042200	0.90968500
Au	-1.26131700	0.31894700	-1.94659300
Au	-0.84209600	-1.69415400	0.36561200
Au	-1.33750300	-2.49135400	-2.31464900
Au	-1.00013700	3.07712400	-1.29691400
Au	-0.25835200	-0.63035800	2.93179400
Au	-3.40295400	0.04913600	0.28056300
Au	-3.41655800	-2.75702400	-0.18962800
Au	-3.82273200	-0.82427400	-2.39265500
Au	-2.90222500	-1.87013600	2.32313000
Au	-3.65925000	1.84355700	-1.90683300
Au	-2.72200800	1.04260600	2.85506400
Au	-3.07581300	2.82043700	0.83459900
Au	-3.86748000	-3.53806700	-2.78159700
Au	-2.27940000	-0.84976200	4.80072200
Au	-3.37240600	4.50818000	-1.31018800
N	8.18452900	0.01950100	2.13814000
S	5.94348700	-0.05292300	-1.45500800
H	6.00639100	1.25846600	-1.75134700

Sum of electronic and zero-point Energies= -4996.750746
Sum of electronic and thermal Energies= -4996.672391
Sum of electronic and thermal Enthalpies= -4996.671447
Sum of electronic and thermal Free Energies= -4996.896512

[(Porph)Ru(NO)(HS)@Au$_{20}$] GS

Ru	7.54951900	-0.00570600	0.32668100
O	9.29051100	0.08071700	2.64282800
C	7.03914000	3.39068400	0.46363700

C	10.26220800	0.48930600	-1.71521800
C	7.84312700	-3.40037400	-0.12031500
C	4.71945900	-0.50033200	2.20409500
C	5.07103800	0.82410600	1.93876300
C	4.35837800	1.98427200	2.40879000
C	4.99926700	3.07638100	1.90613200
C	6.11489800	2.59334700	1.13277000
N	6.12311000	1.22777800	1.17258600
C	8.13265300	2.95260900	-0.28044500
C	9.06932500	3.80136800	-0.97012800
C	9.97684000	2.98685100	-1.57659000
C	9.60296700	1.63127200	-1.26745400
N	8.48594700	1.64950800	-0.48238200
C	9.90078800	-0.83236600	-1.46918400
C	10.57087200	-1.99277100	-1.99832200
C	9.88886000	-3.08477000	-1.55595800
C	8.79704600	-2.60310800	-0.74863400
N	8.84025900	-1.24074100	-0.71363600
C	6.76706100	-2.96146800	0.64563600
C	5.80533800	-3.81141800	1.29860400
C	4.93741300	-2.99793200	1.96269700
C	5.35492700	-1.64221900	1.71368000
N	6.45865800	-1.65680300	0.91319400
H	6.89378600	4.46425600	0.52896400
H	11.13696300	0.64625400	-2.33815800
H	7.94848600	-4.47368300	-0.24197000
H	3.87751300	-0.65849700	2.87287300
H	3.47584700	1.95160900	3.03487200
H	4.75231100	4.12091700	2.04458600
H	9.02580000	4.88249400	-0.98287400
H	10.82548500	3.26675500	-2.18683600
H	11.44632100	-1.96105200	-2.63359300
H	10.09312200	-4.12863800	-1.75485200
H	5.80819900	-4.89256500	1.25242500
H	4.08240600	-3.27594300	2.56568100
Au	3.82995200	-0.09755700	-1.33387900
Au	1.31412700	-1.02474400	-2.06650600
Au	1.64634200	1.59727500	-0.96023000
Au	2.04471600	-0.74200800	0.70870300
Au	-0.13470300	1.03178700	1.19138900
Au	-0.94215200	0.70812500	-1.78998600
Au	-0.50244900	-1.77836100	0.00096900
Au	-1.17371700	-1.94710600	-2.75455900
Au	-0.49948800	3.23529700	-0.56335400
Au	0.27050900	-1.34018700	2.69907100
Au	-2.96456500	0.03971600	0.46131300
Au	-3.15433300	-2.59043300	-0.60810400
Au	-3.58246200	-0.20361400	-2.30164900
Au	-2.46353800	-2.29596900	2.00036400
Au	-3.24974600	2.28142800	-1.25558900
Au	-2.10493500	0.41392800	3.14580100
Au	-2.46910100	2.60516800	1.58741600
Au	-3.78275800	-2.76107000	-3.27237500

Au	-1.66806000	-1.87659600	4.59845200
Au	-2.77796500	4.73677000	-0.11601700
N	8.57835300	0.02026200	1.75359200
S	6.24591200	-0.02549300	-1.76989500
H	6.30426400	1.29470600	-2.02537100

Sum of electronic and zero-point Energies= -4318.869696
Sum of electronic and thermal Energies= -4318.802562
Sum of electronic and thermal Enthalpies= -4318.801618
Sum of electronic and thermal Free Energies= -4319.001116

[(bpb)Ru(NO)(HS)@Ag_{20}] GS

Ru	-6.81087900	-0.02724800	0.48092300
O	-8.79947700	-3.42077900	-0.70658300
O	-8.20422400	3.70236700	-0.54518100
O	-7.20056900	0.05378600	3.35769800
N	-8.25595000	-1.22319600	-0.19863800
N	-8.00851000	1.41645300	-0.20003000
N	-5.87445400	-1.94891800	0.71971200
N	-5.54971600	1.70182200	0.76891800
C	-9.35688200	-0.50431400	-0.68933800
C	-9.22616500	0.91453300	-0.68691800
C	-10.27751500	1.70441800	-1.16304900
C	-11.44280100	1.10413800	-1.63400700
C	-11.57002900	-0.28208000	-1.63778500
C	-10.53408400	-1.08678600	-1.16967600
C	-8.04080200	-2.55233800	-0.27008400
C	-6.68482600	-2.93215700	0.27085000
C	-6.29054400	-4.26229000	0.29666200
C	-5.03579200	-4.58778300	0.80264700
C	-4.21353200	-3.56885600	1.27045600
C	-4.67180700	-2.25725100	1.21229600
C	-7.59101400	2.69881500	-0.17396400
C	-6.19462100	2.82547000	0.38354700
C	-5.59647100	4.07365700	0.47995600
C	-4.30396800	4.16893100	0.98703200
C	-3.64642600	3.00702100	1.37357600
C	-4.30635700	1.78770300	1.24817900
Ag	-3.21133600	-0.20279700	-1.50655600
Ag	-1.10068900	1.56002100	-0.79207300
Ag	-0.55834200	-0.81852500	-2.34391100
Ag	-1.33158000	-1.05870400	0.41997900
Ag	1.33530600	-1.78049400	-0.35524400
Ag	1.59413200	0.97297200	-1.60306800
Ag	0.72941500	0.73113000	1.28961000
Ag	0.98978300	3.28787000	0.00906200
Ag	2.11594600	-1.43186300	-3.08974900
Ag	0.47691400	-1.91069600	2.40627600
Ag	3.57447600	0.06921600	0.49992300
Ag	2.93144900	2.40148300	2.05614500
Ag	3.77155900	2.65101500	-0.75001500
Ag	2.68041700	-0.14627900	3.22488100
Ag	4.32042200	0.33890200	-2.26785300
Ag	3.25869100	-2.54794800	1.64195700

Ag	4.07082100	-2.31467700	-1.04526500
Ag	3.14818500	4.91286000	0.81054200
Ag	2.39103000	-2.72520600	4.31025600
Ag	4.80709600	-2.00759100	-3.73863800
H	-10.16093800	2.78026900	-1.15766200
H	-12.25285700	1.72886500	-1.99887600
H	-12.48003500	-0.74696900	-2.00573000
H	-10.61504900	-2.16592400	-1.17077400
H	-6.98105500	-5.00795000	-0.08049000
H	-4.70661800	-5.62142300	0.83455000
H	-3.22622200	-3.76792700	1.67381600
H	-4.06245000	-1.43490000	1.56902400
H	-6.16331100	4.93893200	0.15582500
H	-3.81712800	5.13493100	1.07607600
H	-2.63288700	3.02535600	1.76029100
H	-3.82270400	0.86015200	1.53256000
N	-7.21481700	0.02067900	2.21003200
S	-5.83162400	-0.16867100	-1.74321100
H	-5.93762900	1.12612700	-2.09177500

Sum of electronic and zero-point Energies= -4599.052442
Sum of electronic and thermal Energies= -4598.984502
Sum of electronic and thermal Enthalpies= -4598.983558
Sum of electronic and thermal Free Energies= -4599.178293

[(Pc)Ru(NO)(HS)@Ag$_{20}$] GS

Ru	-6.58554300	0.00030600	0.63149600
O	-8.35327600	-0.12733500	2.95125000
C	-4.00198300	-4.79607400	1.78309000
C	-2.81329300	-5.12282100	2.43007600
C	-1.97781400	-4.12806400	2.96266300
C	-2.30823800	-2.77839000	2.86482000
C	-3.49912200	-2.44965100	2.22115700
C	-4.13180600	-1.17181500	1.92497900
N	-3.61436600	0.01064600	2.23863500
C	-4.14425000	1.18884900	1.93169000
C	-3.52626700	2.47171000	2.23801200
C	-2.34045000	2.80889000	2.88672700
C	-2.02489600	4.16133800	2.99435600
C	-2.86979300	5.15056100	2.46634200
C	-4.05352500	4.81535000	1.81462700
C	-4.37173800	3.46441500	1.70682700
C	-5.49670900	2.77326600	1.09164700
N	-5.30847400	1.43345800	1.26826300
N	-6.49350200	3.37938500	0.46366400
C	-7.50051400	2.76835600	-0.14473100
C	-8.55113000	3.45166100	-0.88764700
C	-8.80129000	4.79973100	-1.12620000
C	-9.91206200	5.12300300	-1.90047600
C	-10.74858800	4.12576000	-2.42574100
C	-10.49799100	2.77669100	-2.19156400
C	-9.39026700	2.45126700	-1.41457200
C	-8.83810500	1.17242500	-0.98713400
N	-7.73545400	1.42956000	-0.22820300

N	-9.34193500	-0.00702800	-1.32002300
C	-8.82758700	-1.18404300	-0.99333600
C	-9.37295500	-2.46650600	-1.41800800
C	-10.48312000	-2.79931800	-2.18860700
C	-10.72637600	-4.14989600	-2.42117600
C	-9.88051500	-5.14185100	-1.90059000
C	-8.76749200	-4.81148500	-1.13283900
C	-8.52440200	-3.46173400	-0.89581900
C	-7.47405500	-2.77251200	-0.15872900
N	-7.71656300	-1.43376800	-0.24435200
N	-6.46098100	-3.37600700	0.44664800
C	-5.46794600	-2.76424200	1.07618300
N	-5.29231700	-1.42383000	1.25975800
C	-4.33505900	-3.44797600	1.68486400
H	-4.64873400	-5.56242900	1.36786900
H	-2.52496600	-6.16522000	2.52662100
H	-1.05702800	-4.41852700	3.46037600
H	-1.65669200	-2.00744900	3.26658200
H	-1.68164300	2.04233300	3.28508100
H	-1.10878900	4.45857000	3.49656700
H	-2.59305500	6.19534100	2.57066900
H	-4.70792100	5.57733700	1.40337000
H	-8.15115700	5.56863600	-0.72107300
H	-10.13727800	6.16541800	-2.10462000
H	-11.60562500	4.41489100	-3.02647300
H	-11.14104700	2.00287100	-2.59868100
H	-11.13354100	-2.02973000	-2.59200400
H	-11.58501200	-4.44459500	-3.01688900
H	-10.10035500	-6.18570100	-2.10325100
H	-8.11020500	-5.57614600	-0.73125200
Ag	-2.76299200	0.16714700	-1.21839200
Ag	-0.32782500	1.56975300	-1.51195200
Ag	-0.39559200	-1.32585400	-1.60362600
Ag	-0.86835600	0.05424400	0.91820700
Ag	1.52217600	-1.51038400	0.52612600
Ag	2.09942300	0.07261800	-1.99143700
Ag	1.59867100	1.51080100	0.63456000
Ag	2.13089000	2.94121000	-1.80930500
Ag	1.98689300	-2.80277500	-1.99504500
Ag	1.06318800	-0.06506200	2.98009300
Ag	4.16012400	-0.05725600	0.21823400
Ag	4.14551500	2.81747500	0.33416300
Ag	4.63134400	1.41974800	-2.21239200
Ag	3.62925000	1.33770600	2.68178100
Ag	4.55733600	-1.39063400	-2.30522800
Ag	3.54602400	-1.59868700	2.57434300
Ag	4.00654200	-2.92819700	0.13831300
Ag	4.63537600	4.22676000	-2.05369700
Ag	3.04471800	-0.21290800	4.97400900
Ag	4.42062300	-4.19806700	-2.33809700
N	-7.59469300	-0.04355500	2.10325800
S	-5.36979800	0.05100200	-1.47573800
H	-5.38247700	-1.26873700	-1.73671700

Sum of electronic and zero-point Energies=	-5202.746345
Sum of electronic and thermal Energies=	-5202.669898
Sum of electronic and thermal Enthalpies=	-5202.668953
Sum of electronic and thermal Free Energies=	-5202.880175

[(Porph)Ru(NO)(HS)@Ag$_{20}$] GS

Ru	7.12239300	-0.01453100	0.30604500
O	8.93339900	0.15225100	2.57091100
C	6.76340500	3.40471800	0.32872200
C	9.89385400	0.29536600	-1.69139200
C	7.30197300	-3.43506800	0.00921200
C	4.22012500	-0.32547300	2.10613000
C	4.63781000	0.97272400	1.81631000
C	3.95825400	2.17701300	2.22106200
C	4.66678600	3.22423100	1.71376800
C	5.78766800	2.66905700	0.99762600
N	5.73681600	1.30776900	1.08297000
C	7.85161700	2.89657500	-0.37767500
C	8.83646800	3.68090700	-1.07833900
C	9.71582800	2.80761400	-1.64269800
C	9.27680600	1.47995700	-1.29536400
N	8.15048400	1.57421300	-0.53126900
C	9.47553900	-1.00156900	-1.40407000
C	10.11273100	-2.20661300	-1.87284800
C	9.38455000	-3.25434400	-1.39838900
C	8.29632700	-2.70028000	-0.63253500
N	8.38793900	-1.34059000	-0.65405700
C	6.22489900	-2.92437800	0.72928100
C	5.20861700	-3.70994100	1.38148600
C	4.34856700	-2.83698100	1.97709600
C	4.82903000	-1.50920500	1.69070200
N	5.96196500	-1.59986500	0.93696500
H	6.66208600	4.48508500	0.35603100
H	10.78486400	0.39355800	-2.30356700
H	7.36913900	-4.51580000	-0.06602100
H	3.32193100	-0.42629100	2.70889200
H	3.05129900	2.20362600	2.81145700
H	4.46122600	4.28242800	1.81124200
H	8.84172900	4.76198700	-1.12658600
H	10.58571100	3.02985500	-2.24683100
H	11.00032400	-2.23271900	-2.49136400
H	9.55507100	-4.31230100	-1.54932900
H	5.17097800	-4.79156600	1.37993600
H	3.45907100	-3.05671100	2.55398700
Ag	3.26688700	-0.07190800	-1.41449400
Ag	0.64825300	-0.72917000	-2.30367800
Ag	1.11567700	1.62916300	-0.69726200
Ag	1.38205400	-1.01438000	0.47583100
Ag	-0.74757600	0.71842900	1.31599700
Ag	-1.55449300	1.01315900	-1.59243100
Ag	-1.25660800	-1.75423100	-0.37971400
Ag	-1.99350000	-1.37680700	-3.12687700
Ag	-1.03077500	3.29787200	0.08222000

Ag	-0.47830500	-1.93482600	2.38989400
Ag	-3.56510000	0.02259000	0.45266800
Ag	-3.96992000	-2.33380800	-1.14576200
Ag	-4.25679000	0.33428300	-2.32297100
Ag	-3.22593500	-2.60535100	1.55709000
Ag	-3.78274300	2.62590400	-0.75274400
Ag	-2.72479000	-0.21788600	3.19290900
Ag	-2.99626100	2.34033600	2.05994500
Ag	-4.66255200	-1.98749500	-3.84867900
Ag	-2.41901000	-2.80775100	4.24331800
Ag	-3.24084700	4.87062000	0.86087200
N	8.16413700	0.04299900	1.73205800
S	5.84776800	-0.04221300	-1.77750800
H	5.90491100	1.27713800	-2.03427100

Sum of electronic and zero-point Energies= -4524.861977
Sum of electronic and thermal Energies= -4524.795727
Sum of electronic and thermal Enthalpies= -4524.794782
Sum of electronic and thermal Free Energies= -4524.985848

[(bpb)Ru(NO)(HS)@Au_{20}] MSII

Ru	7.46777300	-0.01184800	-0.43078400
O	9.34005000	-3.51196200	0.59039500
O	9.12677100	3.63622800	0.45776500
N	8.84310900	-1.27221900	0.20993700
N	8.75136200	1.35686200	0.18890200
N	6.48197100	-1.90655300	-0.80320500
N	6.34346200	1.81183700	-0.81847700
C	9.95864400	-0.62581600	0.76506300
C	9.91096400	0.79924800	0.75000200
C	10.97439700	1.53124300	1.29100200
C	12.07043900	0.86674600	1.83120100
C	12.11692800	-0.52609400	1.84624500
C	11.06806800	-1.27351100	1.32105700
C	8.59983800	-2.61281500	0.20102200
C	7.25215100	-2.93329200	-0.38743600
C	6.83356500	-4.25205700	-0.49843500
C	5.58853800	-4.52033400	-1.05803500
C	4.80500300	-3.45737000	-1.49402300
C	5.29055300	-2.16160000	-1.35101200
C	8.43332400	2.67905800	0.12211300
C	7.05943500	2.89602400	-0.45409000
C	6.55911400	4.18342200	-0.59666800
C	5.28724000	4.35779700	-1.13157900
C	4.55704900	3.23552700	-1.50804800
C	5.12347700	1.97625900	-1.33680900
Au	3.80517200	-0.11956900	1.18585700
Au	1.76007900	1.53785400	0.27427600
Au	1.26151300	-0.49117700	2.25618200
Au	1.85698800	-1.25982300	-0.44813800
Au	-0.73412500	-1.73303400	0.62695400
Au	-0.84071700	1.24582400	1.39914600
Au	-0.17031600	0.43632700	-1.51190600
Au	-0.24787300	3.13710700	-0.64107800

Au	-1.25833900	-0.84632200	3.26851200
Au	-0.04346900	-2.34817700	-2.06923900
Au	-3.03156000	0.04811100	-0.41530000
Au	-2.38319500	1.99129700	-2.38390500
Au	-3.02061900	2.77115800	0.40529800
Au	-2.28244500	-0.63418500	-3.07399600
Au	-3.51291800	0.85627000	2.26918900
Au	-2.81506300	-2.71865500	-1.03466500
Au	-3.41357800	-2.00471000	1.51685200
Au	-2.40353500	4.60702800	-1.54538200
Au	-2.10928200	-3.32174000	-3.62106500
Au	-3.88937900	-1.16882100	4.08513500
S	6.26289700	-0.08830100	1.59038000
H	10.92534700	2.61174300	1.27396100
H	12.89350500	1.44473600	2.24092400
H	12.97635800	-1.03909300	2.26763400
H	11.09127800	-2.35490600	1.32772000
H	7.49645700	-5.03411600	-0.14633600
H	5.23772600	-5.54246400	-1.15655400
H	3.82730100	-3.61193900	-1.93823400
H	4.71175500	-1.30677100	-1.68291700
H	7.18193100	5.01483500	-0.28709800
H	4.87308600	5.35330200	-1.25451200
H	3.55955500	3.31824400	-1.92647600
H	4.58398100	1.07755500	-1.61508400
H	6.32775200	1.21654300	1.92241200
O	8.55286900	-0.01654600	-2.65199300
N	7.40525000	0.03203700	-2.41018500

Sum of electronic and zero-point Energies= -4393.028612
Sum of electronic and thermal Energies= -4392.958218
Sum of electronic and thermal Enthalpies= -4392.957274
Sum of electronic and thermal Free Energies= -4393.165652

[(Pc)Ru(NO)(HS)@Au_{20}] MSII

Ru	7.30438800	-0.01365100	-0.63131700
Au	3.48652900	0.03177800	0.98685000
Au	1.42560600	1.46342300	-0.21577300
Au	1.00965200	0.01523100	2.23830500
Au	1.43825000	-1.43688200	-0.21015500
Au	-1.09006800	-1.54561000	1.08733300
Au	-1.10441900	1.55161700	1.08341100
Au	-0.62333600	0.00302000	-1.55180500
Au	-0.60946000	2.83940700	-1.39944500
Au	-1.44587800	0.00368300	3.43742400
Au	-0.58259800	-2.83272300	-1.39450400
Au	-3.42091300	-0.00920900	-0.23462900
Au	-2.86168800	1.34965600	-2.67396300
Au	-3.32716700	2.82876600	-0.14534400
Au	-2.84881500	-1.36517700	-2.67237600
Au	-3.72777500	1.46991900	2.17254000
Au	-3.29922200	-2.84575200	-0.14214900
Au	-3.71416400	-1.48870900	2.17431800
Au	-2.79567700	4.09385500	-2.52889900

Au	-2.75327000	-4.10865100	-2.52480400
Au	-4.02504900	-0.00940400	4.46235500
S	5.95504700	0.02202000	1.30821800
O	8.78074000	-0.25585600	-2.36254000
N	7.87345500	0.46260500	-2.47840100
H	6.10078100	1.31532200	1.65396000
C	4.78629500	4.80528100	-1.87560700
C	3.63770600	5.13837500	-2.58884400
C	2.82410000	4.14831700	-3.16181000
C	3.13574100	2.79615300	-3.03829100
C	4.28460600	2.45971900	-2.32612500
C	4.89516600	1.17721200	-2.00005800
N	4.42127300	-0.00533100	-2.37658400
C	4.92786900	-1.18612000	-2.05770200
C	4.30478700	-2.46330200	-2.38503500
C	3.13923700	-2.78544600	-3.07497700
C	2.80769700	-4.13435200	-3.18991300
C	3.61519000	-5.13254800	-2.62436200
C	4.77775500	-4.81129600	-1.92693600
C	5.11362500	-3.46565800	-1.81677200
C	6.22475700	-2.78497000	-1.16294400
N	6.07555600	-1.44790600	-1.36003700
N	7.18174300	-3.39556700	-0.47631100
C	8.15692500	-2.78374600	0.17595700
C	9.18347100	-3.46981000	0.94425600
C	9.42045600	-4.82133100	1.18255900
C	10.51224400	-5.15335100	1.97749200
C	11.34694200	-4.16221000	2.52046000
C	11.11279500	-2.81186200	2.28360700
C	10.01970900	-2.47652100	1.48855800
C	9.49437300	-1.19381700	1.04791300
N	8.39721000	-1.43889600	0.26682900
N	10.02000100	-0.02360800	1.37109100
C	9.53950100	1.15869000	1.00955900
C	10.08302400	2.43822200	1.44623100
C	11.17655500	2.76572100	2.24160900
C	11.42077000	4.11570800	2.47916800
C	10.59045000	5.11107000	1.94132500
C	9.49207700	4.78507600	1.15057000
C	9.25009500	3.43657600	0.90650900
C	8.20870800	2.75217200	0.15195100
N	8.45817500	1.40678000	0.22067700
N	7.20119200	3.36200200	-0.45333000
C	6.20053600	2.76390400	-1.09005600
N	6.00055800	1.42896000	-1.25060300
C	5.10098300	3.45500900	-1.75018400
H	5.41838600	5.56905400	-1.43387100
H	3.36582500	6.18283300	-2.70835900
H	1.93523100	4.44378300	-3.71171600
H	2.50622700	2.03020500	-3.48029000
H	2.51154900	-2.01230000	-3.50692000
H	1.90671400	-4.41939900	-3.72545100
H	3.32744500	-6.17377000	-2.73462100

H	5.40313100	-5.58114600	-1.48619600
H	8.77347200	-5.58398800	0.76121700
H	10.72636400	-6.19748100	2.18446900
H	12.19006300	-4.45951800	3.13662400
H	11.75556500	-2.04370200	2.70142600
H	11.81459300	1.99353400	2.65949800
H	12.26791900	4.40656400	3.09291700
H	10.81018500	6.15397000	2.14873800
H	8.84533000	5.55218600	0.73676500

Sum of electronic and zero-point Energies= -4996.708408
Sum of electronic and thermal Energies= -4996.629854
Sum of electronic and thermal Enthalpies= -4996.628910
Sum of electronic and thermal Free Energies= -4996.852604

[(Porph)Ru(NO)(HS)@Au$_{20}$] MSII

Ru	-7.63973300	0.01692400	-0.31203300
Au	-3.84086700	0.04104400	1.22099700
Au	-1.91403600	-1.32582500	-0.26235000
Au	-1.29901100	-0.15889800	2.30972300
Au	-1.80766800	1.56155900	0.07358100
Au	0.79914700	1.44538800	1.22291600
Au	0.67982300	-1.62698000	0.86861800
Au	0.11105500	0.21190800	-1.54723600
Au	0.00022900	-2.62235600	-1.70741400
Au	1.22434400	-0.37046900	3.35606900
Au	0.19589300	3.01303200	-1.07734700
Au	2.97374600	-0.01919200	-0.41372800
Au	2.22587700	-1.07435800	-2.94682300
Au	2.77155200	-2.84409900	-0.62938200
Au	2.32179000	1.62117400	-2.64542800
Au	3.36183400	-1.76797200	1.79237500
Au	2.97532300	2.79438200	-0.00520000
Au	3.48075200	1.17365300	2.11646200
Au	2.06458100	-3.81241800	-3.10374800
Au	2.35794600	4.32893500	-2.19919600
Au	3.86613300	-0.56350400	4.20371400
S	-6.28651400	-0.02998400	1.61069100
O	-9.12768000	0.21352900	-2.04026500
N	-8.14526700	-0.40370300	-2.17520400
H	-6.37524100	-1.34454200	1.89202800
C	-7.24210000	-3.40183300	-0.34086700
C	-10.41323800	-0.33151700	1.66121900
C	-7.82889200	3.43486000	0.00192300
C	-4.76029400	0.35710900	-2.13959700
C	-5.12117500	-0.95138300	-1.80317400
C	-4.42317700	-2.14742600	-2.20903600
C	-5.11319900	-3.20593100	-1.68977000
C	-6.23748400	-2.66336000	-0.97221100
N	-6.18607800	-1.30549500	-1.03805400
C	-8.36345600	-2.90424300	0.31576600
C	-9.34756700	-3.70465600	1.00170700
C	-10.24246000	-2.84608900	1.56088000
C	-9.81494200	-1.51106900	1.22249800

N	-8.69609400	-1.58082000	0.44779700
C	-9.96846100	0.96346200	1.41910900
C	-10.58924000	2.16022800	1.92025800
C	-9.86205000	3.21547500	1.45949400
C	-8.78814200	2.67944200	0.66690200
N	-8.88048100	1.31118300	0.66144200
C	-6.77781200	2.94242300	-0.76974900
C	-5.76535900	3.73549100	-1.42393000
C	-4.91230000	2.86680200	-2.03244400
C	-5.39062600	1.53495200	-1.74796800
N	-6.53350200	1.62324300	-1.00097300
H	-7.13392300	-4.48182200	-0.36240500
H	-11.30055400	-0.43302400	2.27802400
H	-7.90085500	4.51295200	0.10429600
H	-3.86552800	0.46700500	-2.74515600
H	-3.53538000	-2.16536700	-2.82875000
H	-4.89651600	-4.26118600	-1.79360700
H	-9.33525500	-4.78561100	1.05111700
H	-11.11098500	-3.08121300	2.16203200
H	-11.47309000	2.17835700	2.54437200
H	-10.03166100	4.27048800	1.63044700
H	-5.72372400	4.81679100	-1.40916700
H	-4.02845900	3.09163200	-2.61570200

Sum of electronic and zero-point Energies= -4318.828138
Sum of electronic and thermal Energies= -4318.759873
Sum of electronic and thermal Enthalpies= -4318.758929
Sum of electronic and thermal Free Energies= -4318.961296

[(bpb)Ru(NO)(HS)@Ag_{20}] MSII

Ru	-6.89542300	-0.02231600	0.36454000
O	-8.95444600	-3.46594500	-0.48795900
O	-8.52531400	3.67670500	-0.36258100
N	-8.35780200	-1.24307500	-0.15314700
N	-8.18908400	1.38668100	-0.13459800
N	-5.93698300	-1.94846200	0.64288900
N	-5.69134800	1.77104700	0.65991000
C	-9.49865500	-0.56065500	-0.60641300
C	-9.40875200	0.86238300	-0.59326000
C	-10.49506300	1.62585200	-1.03580700
C	-11.65396800	0.99567400	-1.47848000
C	-11.74172600	-0.39463200	-1.49138300
C	-10.67169600	-1.17339700	-1.06168800
C	-8.15648700	-2.58876300	-0.16504300
C	-6.77126800	-2.95120100	0.29910600
C	-6.38106900	-4.28142200	0.36917600
C	-5.09563900	-4.58629800	0.80650600
C	-4.24353900	-3.54705300	1.16333100
C	-4.70573100	-2.23833900	1.07087600
C	-7.83240200	2.69819600	-0.08937100
C	-6.40936300	2.87622500	0.36892200
C	-5.86656700	4.14943500	0.47833600
C	-4.54715600	4.28755800	0.89726200
C	-3.81239300	3.14378200	1.18965100

C	-4.42407600	1.90027600	1.06140300
Ag	-3.16789000	-0.23710500	-1.50763100
Ag	-1.07368700	1.54921000	-0.79620900
Ag	-0.51966100	-0.84117900	-2.33777900
Ag	-1.29763900	-1.06842000	0.43423000
Ag	1.37324600	-1.77937400	-0.34061700
Ag	1.62266300	0.96562300	-1.60258800
Ag	0.75528900	0.73405200	1.29212300
Ag	1.00810500	3.28649400	-0.00038500
Ag	2.15554300	-1.44269500	-3.07868800
Ag	0.51488900	-1.90576000	2.42053200
Ag	3.60560400	0.08010500	0.50624700
Ag	2.95159700	2.41516100	2.05321700
Ag	3.79227500	2.65732100	-0.75468400
Ag	2.70963800	-0.12919900	3.23107700
Ag	4.35191400	0.34085800	-2.26296400
Ag	3.29753600	-2.53581600	1.65796200
Ag	4.10975800	-2.30989600	-1.02945700
Ag	3.15813600	4.92252100	0.79715100
Ag	2.43043100	-2.70554900	4.32754700
Ag	4.84694200	-2.01226500	-3.72378700
S	-5.83049200	-0.12751700	-1.72134400
H	-10.41320300	2.70442200	-1.02159500
H	-12.49265400	1.59911100	-1.81313700
H	-12.64917400	-0.88171400	-1.83604700
H	-10.72619300	-2.25375300	-1.06784400
H	-7.09601000	-5.04352800	0.08084200
H	-4.76605700	-5.61829600	0.87026200
H	-3.23015300	-3.72799400	1.50685100
H	-4.07283400	-1.40020100	1.34261100
H	-6.49414900	4.99878500	0.23374700
H	-4.09950300	5.27168800	0.99248200
H	-2.77502500	3.19554700	1.50446600
H	-3.87994200	0.98622000	1.27513700
H	-5.85757100	1.18243600	-2.03503400
O	-7.77493100	-0.00721500	2.67275200
N	-6.65201700	0.00857000	2.32546300

Sum of electronic and zero-point Energies= -4599.024703
Sum of electronic and thermal Energies= -4598.956300
Sum of electronic and thermal Enthalpies= -4598.955356
Sum of electronic and thermal Free Energies= -4599.150109

[(Pc)Ru(NO)(HS)@Ag$_{20}$] MSII

Ru	-6.73392500	0.01009100	-0.60483800
Ag	-2.71914200	-0.15048700	1.14238400
Ag	-0.59094400	-1.55077400	-0.08306200
Ag	-0.16767600	-0.05201400	2.36237300
Ag	-0.69076000	1.36118500	-0.12134700
Ag	1.90227900	1.50485800	1.10686100
Ag	2.00860900	-1.51761400	1.15454300
Ag	1.45230100	-0.05344500	-1.43776100
Ag	1.54298300	-2.92466900	-1.31811400
Ag	2.40797400	0.05267700	3.53931300

Ag	1.36082600	2.82218500	-1.39264500
Ag	4.22052100	0.05225500	-0.21205000
Ag	3.68750900	-1.40707800	-2.64398600
Ag	4.23909600	-2.82169700	-0.11655300
Ag	3.59624400	1.40934000	-2.68398000
Ag	4.66543100	-1.36727600	2.26345900
Ag	4.04893000	2.92481700	-0.19931200
Ag	4.56202500	1.56148600	2.21435900
Ag	3.73745400	-4.21846800	-2.51501400
Ag	3.45910300	4.22551700	-2.62952700
Ag	5.01899400	0.15746500	4.60731600
S	-5.37620500	-0.01720300	1.31808000
O	-8.18488400	0.23637500	-2.39971600
N	-7.25241300	-0.45778200	-2.47078500
H	-5.61676500	-1.27344000	1.73849200
C	-4.15945500	-4.80145600	-1.77609400
C	-2.98132000	-5.13008600	-2.44240400
C	-2.14918100	-4.13644700	-2.98055400
C	-2.47306000	-2.78552300	-2.87140100
C	-3.65321200	-2.45407200	-2.21080900
C	-4.28165600	-1.17394000	-1.90674400
N	-3.79484200	0.01162900	-2.25793000
C	-4.31796200	1.19097800	-1.95790600
C	-3.68695200	2.46963900	-2.26194000
C	-2.49378200	2.79525000	-2.90111300
C	-2.16079300	4.14543000	-3.00196500
C	-2.99384700	5.14131100	-2.47135700
C	-4.18236600	4.81627200	-1.82016800
C	-4.51917400	3.47001700	-1.72348000
C	-5.65035100	2.78525700	-1.10884100
N	-5.49014200	1.44917300	-1.30124200
N	-6.63071800	3.39281800	-0.45208300
C	-7.62187500	2.77743500	0.17238300
C	-8.66718500	3.46049300	0.91905400
C	-8.91208100	4.81070600	1.15662100
C	-10.02074600	5.13866800	1.92987300
C	-10.86395000	4.14437200	2.45320300
C	-10.62137200	2.79511000	2.21768500
C	-9.51199900	2.46398200	1.44382100
C	-8.97240600	1.18335900	1.01223400
N	-7.86130500	1.43227700	0.25380100
N	-9.49860300	0.01061300	1.32620100
C	-9.00258000	-1.16980600	0.97838500
C	-9.55194400	-2.45124900	1.40277100
C	-10.66282900	-2.78279300	2.17220200
C	-10.90833500	-4.13345200	2.40435000
C	-10.06245000	-5.12610500	1.88593200
C	-8.94715800	-4.79618000	1.12095800
C	-8.70325500	-3.44683300	0.88264400
C	-7.64667000	-2.75862700	0.15253600
N	-7.90097600	-1.41379600	0.21728200
N	-6.62508300	-3.36632400	-0.43120100
C	-5.61079500	-2.76508700	-1.04363500

N	-5.41340100	-1.43076900	-1.20160000
C	-4.48600000	-3.45292000	-1.66520700
H	-4.80438300	-5.56807500	-1.35829100
H	-2.69975000	-6.17338300	-2.54960400
H	-1.23475800	-4.42717800	-3.48981300
H	-1.82659900	-2.01691300	-3.28333100
H	-1.84493300	2.02412500	-3.30438200
H	-1.23718800	4.43209200	-3.49689300
H	-2.70496000	6.18344700	-2.57007800
H	-4.82610300	5.58446400	-1.40345500
H	-8.25798800	5.57601800	0.75119500
H	-10.24122000	6.18186300	2.13511100
H	-11.72011900	4.43800400	3.05299800
H	-11.27029800	2.02434700	2.62111600
H	-11.31297600	-2.01263400	2.57505500
H	-11.76858900	-4.42700700	2.99834400
H	-10.28347800	-6.16979800	2.08814200
H	-8.28863700	-5.56130300	0.72218100

Sum of electronic and zero-point Energies= -5202.705080
Sum of electronic and thermal Energies= -5202.627264
Sum of electronic and thermal Enthalpies= -5202.626320
Sum of electronic and thermal Free Energies= -5202.842846

[(Porph)Ru(NO)(HS)@Ag_{20}] MSII

Ru	-7.30842000	0.01082900	-0.23015000
Ag	-3.27868900	0.08225700	1.13406600
Ag	-1.25055100	-1.40332700	-0.15726600
Ag	-0.69898300	0.03029000	2.30522400
Ag	-1.19485700	1.50370100	-0.14150600
Ag	1.42788200	1.51599600	1.04459100
Ag	1.36999300	-1.50430800	1.02091600
Ag	0.84342400	0.02618000	-1.53549400
Ag	0.79350500	-2.84611700	-1.46177500
Ag	1.90449900	-0.02523700	3.43212600
Ag	0.89271200	2.89632400	-1.43191700
Ag	3.63087100	-0.02724100	-0.35483100
Ag	2.99739900	-1.41451700	-2.80143900
Ag	3.51447900	-2.90111900	-0.31667600
Ag	3.04801900	1.40519600	-2.78636200
Ag	4.05270200	-1.51432500	2.07798900
Ag	3.61543300	2.84653600	-0.28129500
Ag	4.10957100	1.41319100	2.09911600
Ag	2.89703300	-4.23001600	-2.72083600
Ag	3.04764300	4.21860400	-2.67365500
Ag	4.53876500	-0.07690400	4.44719900
S	-5.89624100	-0.01215700	1.61169400
O	-8.77482500	0.07165900	-2.11031500
N	-7.62240700	-0.08603100	-2.20442800
H	-5.90202100	-1.33830500	1.85270100
C	-7.31696400	-3.44303000	-0.10171400
C	-10.18265600	0.10223600	1.57122400
C	-7.13866300	3.46078100	-0.17349300
C	-4.37412800	-0.08374900	-1.98697500

C	-4.91001000	-1.32490300	-1.63038200
C	-4.32716700	-2.60818500	-1.93972300
C	-5.14551300	-3.55841300	-1.40312800
C	-6.23542200	-2.86140100	-0.76726700
N	-6.05310900	-1.52296300	-0.91786700
C	-8.37313500	-2.77286400	0.50469600
C	-9.46742500	-3.40436800	1.19537300
C	-10.27549400	-2.41241700	1.65999900
C	-9.68377600	-1.16078700	1.26099500
N	-8.54213200	-1.40942000	0.55132900
C	-9.61004000	1.32978900	1.24990000
C	-10.13012900	2.61749700	1.63175000
C	-9.27263500	3.55712900	1.14681500
C	-8.21863500	2.85764600	0.45984400
N	-8.45824300	1.50620500	0.53246600
C	-6.09943500	2.80885400	-0.84028000
C	-4.98105800	3.43585000	-1.50095600
C	-4.21527200	2.43432400	-2.01925500
C	-4.85827900	1.18869200	-1.67365600
N	-5.99155500	1.46038400	-0.96565300
H	-7.33491300	-4.52721700	-0.04978300
H	-11.10367500	0.13205500	2.14512300
H	-7.09954000	4.54527600	-0.14420400
H	-3.44744900	-0.11239900	-2.55317400
H	-3.40453400	-2.75053200	-2.48850500
H	-5.03258600	-4.63436400	-1.43340200
H	-9.58926400	-4.47402600	1.30583500
H	-11.19171700	-2.50714200	2.22828800
H	-11.03711600	2.77097000	2.20196800
H	-9.33725100	4.63341100	1.23964200
H	-4.81451100	4.50388100	-1.55545000
H	-3.28930300	2.51710600	-2.57462100

Sum of electronic and zero-point Energies= -4524.823054
Sum of electronic and thermal Energies= -4524.755673
Sum of electronic and thermal Enthalpies= -4524.754728
Sum of electronic and thermal Free Energies= -4524.948697

[(bpb)Ru(NO)(HS)@Au_{20}] MSI

Ru	7.32840500	-0.02122100	-0.44613600
O	9.23703100	-3.47386600	0.68210200
O	8.82152600	3.68368100	0.51749900
O	7.87021600	0.03030300	-2.29960600
N	8.72304600	-1.24691300	0.25780500
N	8.55079400	1.39182100	0.23833100
N	6.40059700	-1.92085000	-0.83773600
N	6.17440800	1.73996200	-0.89694700
C	9.80261100	-0.56243800	0.83184700
C	9.71137800	0.86301900	0.81827500
C	10.74545600	1.62490700	1.37508200
C	11.85327600	0.99399600	1.93241400
C	11.94205000	-0.39688800	1.94668300
C	10.92456900	-1.17477100	1.40313300
C	8.50289200	-2.58279500	0.25261900

C	7.18039000	-2.92964200	-0.38665600
C	6.79255100	-4.25705900	-0.50242900
C	5.57354500	-4.55739000	-1.10249900
C	4.78098500	-3.51585400	-1.57156700
C	5.23208200	-2.20858800	-1.41891700
C	8.18850300	2.69222100	0.14951200
C	6.83486600	2.85210800	-0.50025400
C	6.29769800	4.11987600	-0.67571900
C	5.05051200	4.25055600	-1.27840600
C	4.37762200	3.10389300	-1.68510800
C	4.97506800	1.86471700	-1.47344500
Au	3.81797000	-0.11182500	1.24417700
Au	1.78007800	1.53875100	0.30237500
Au	1.25934400	-0.47988000	2.28574600
Au	1.88407900	-1.25873800	-0.40656300
Au	-0.71802100	-1.73101800	0.64126200
Au	-0.83346900	1.25325600	1.40125700
Au	-0.13125800	0.43080900	-1.50179600
Au	-0.22096000	3.13357000	-0.64119200
Au	-1.27171600	-0.83450100	3.27232100
Au	0.00251300	-2.35645800	-2.04515900
Au	-3.00607000	0.04467500	-0.43282000
Au	-2.33586600	1.98023800	-2.40191000
Au	-3.00537400	2.77065200	0.37612000
Au	-2.22766700	-0.64825000	-3.07973300
Au	-3.51462400	0.86357300	2.24331500
Au	-2.78018500	-2.72475600	-1.03755000
Au	-3.40768700	-1.99960700	1.50396600
Au	-2.36895100	4.59911100	-1.57419200
Au	-2.04845800	-3.33768200	-3.61433600
Au	-3.91338200	-1.15336800	4.06270500
N	7.79404700	0.09826000	-3.43654200
S	6.26039400	-0.11148500	1.65891400
H	10.66081200	2.70366500	1.35778300
H	12.65101100	1.59628200	2.35724100
H	12.80929600	-0.88408300	2.38269400
H	10.97836200	-2.25555000	1.40862600
H	7.45924300	-5.02149700	-0.12006000
H	5.24927000	-5.58798000	-1.20485400
H	3.82239000	-3.69553300	-2.04663000
H	4.64258700	-1.36885400	-1.76944700
H	6.87502700	4.97257800	-0.33715300
H	4.61135000	5.23161000	-1.42879100
H	3.40204600	3.15204500	-2.15685300
H	4.47806300	0.94723600	-1.76792500
H	6.35435600	1.18604300	2.00970900

Sum of electronic and zero-point Energies= -4392.995525
Sum of electronic and thermal Energies= -4392.925388
Sum of electronic and thermal Enthalpies= -4392.924444
Sum of electronic and thermal Free Energies= -4393.131869

[(Pc)Ru(NO)(HS)@Au$_{20}$] MSI

Ru	7.09679200	-0.00178700	-0.54961300
O	8.09501500	-0.00031900	-2.19514300

Au	3.46429600	-0.13163100	1.23310700
Au	1.33565900	1.59866700	0.73468900
Au	0.95828900	-0.87115900	2.15210400
Au	1.58176700	-0.95428400	-0.66674300
Au	-0.96703300	-1.79369300	0.25595400
Au	-1.24027800	0.90483600	1.73757500
Au	-0.54160300	0.84225000	-1.27054900
Au	-0.76189600	3.26010300	0.21037200
Au	-1.53004600	-1.59331200	3.03575000
Au	-0.28918700	-1.70886700	-2.50230000
Au	-3.36746200	0.04682100	-0.33127500
Au	-2.83229100	2.44163200	-1.77594600
Au	-3.51021000	2.49394800	1.10704700
Au	-2.60296500	0.06813800	-3.07028500
Au	-3.89167200	0.16914600	2.45847400
Au	-3.02020000	-2.48000600	-1.59434800
Au	-3.61625800	-2.42547800	1.05486500
Au	-3.01067500	4.77506700	-0.34030700
Au	-2.30969700	-2.39620900	-4.25086800
Au	-4.13909600	-2.24086400	3.74300300
N	8.67991600	0.00056600	-3.15936700
S	5.93154300	-0.06339400	1.51110700
H	6.00025000	1.24267500	1.83317200
C	10.58635800	3.64877200	1.77776500
C	10.62711800	5.03817600	1.84791000
C	9.63268300	5.82653000	1.24761900
C	8.56982400	5.24744000	0.56039800
C	8.52712200	3.85809000	0.48812700
C	7.58292200	2.93992600	-0.13450500
N	6.49234800	3.31403900	-0.78928700
C	5.61381500	2.49392500	-1.34873100
C	4.41441800	2.92800400	-2.05343200
C	3.89345700	4.19115600	-2.32066100
C	2.69711900	4.26112500	-3.03014200
C	2.03816400	3.09874000	-3.46093300
C	2.55816000	1.83378600	-3.19585300
C	3.75519800	1.76154500	-2.48759600
C	4.55694400	0.63444500	-2.02925000
N	5.64528000	1.12991400	-1.37527700
N	4.23528900	-0.64002100	-2.21684200
C	4.92557300	-1.68404500	-1.77490400
C	4.52309100	-3.07296700	-1.95454500
C	3.41996900	-3.65643700	-2.57350100
C	3.32824300	-5.04593500	-2.55488800
C	4.31276800	-5.83229500	-1.93588500
C	5.41593500	-5.25130400	-1.31678500
C	5.50964200	-3.86247700	-1.33226300
C	6.49964700	-2.94377900	-0.78522900
N	6.09984800	-1.67282400	-1.08217300
N	7.58069500	-3.31777100	-0.11595200
C	8.48219700	-2.49761800	0.40652300
C	9.64610600	-2.93170700	1.16794600
C	10.12043400	-4.19426400	1.51098900

C	11.28763800	-4.26272500	2.26641300
C	11.96219500	-3.09983100	2.67007000
C	11.48845600	-1.83590000	2.32989600
C	10.32276800	-1.76484500	1.57309700
C	9.56012700	-0.63672100	1.05400200
N	8.49738900	-1.13484300	0.35910800
N	9.87155700	0.63536500	1.25819900
C	9.17438200	1.67775100	0.82829100
N	8.02498700	1.66851700	0.09166800
C	9.52486900	3.06709000	1.09047500
H	11.35329300	3.03652500	2.24155800
H	11.44172500	5.52409700	2.37644600
H	9.69616900	6.90785100	1.32286500
H	7.79907900	5.85438800	0.09594800
H	4.40616700	5.08819000	-1.98800800
H	2.26589700	5.23122800	-3.25897200
H	1.10567200	3.18940900	-4.01063200
H	2.04481700	0.93389300	-3.52113500
H	2.65613300	-3.04646900	-3.04756900
H	2.48056100	-5.53272100	-3.02820900
H	4.20918600	-6.91313800	-1.94163300
H	6.17808600	-5.85635700	-0.83608300
H	9.59568000	-5.09157800	1.19851800
H	11.68556500	-5.23217100	2.55069000
H	12.86976300	-3.19095600	3.25919100
H	12.00710400	-0.93489700	2.64191200

Sum of electronic and zero-point Energies= -4996.691351
Sum of electronic and thermal Energies= -4996.612735
Sum of electronic and thermal Enthalpies= -4996.611791
Sum of electronic and thermal Free Energies= -4996.837177

[(Porph)Ru(NO)(HS)@Au$_{20}$] MSI

Ru	7.55328200	-0.01908700	-0.28270500
O	8.73022800	-0.03688500	-1.77819500
Au	3.86298000	0.09051800	1.17518300
Au	1.72456700	1.67648200	0.38147100
Au	1.35845000	-0.53428000	2.16775800
Au	2.00433900	-1.02942100	-0.62599300
Au	-0.54714200	-1.73858600	0.44831600
Au	-0.84635300	1.15883600	1.51892500
Au	-0.17365900	0.61903700	-1.45598300
Au	-0.39105600	3.23297600	-0.36884500
Au	-1.11126900	-1.12508000	3.17732600
Au	0.09562000	-2.07953000	-2.29083700
Au	-2.98718800	-0.02624000	-0.38140500
Au	-2.48363600	2.11690400	-2.18058200
Au	-3.13456300	2.61314900	0.67036400
Au	-2.25218300	-0.42707600	-3.09796500
Au	-3.48197500	0.51870700	2.36518900
Au	-2.62618400	-2.71236300	-1.23950000
Au	-3.19130800	-2.25322800	1.37136200
Au	-2.65530600	4.64230500	-1.11750200
Au	-1.94633400	-3.04580900	-3.88139100
Au	-3.71083300	-1.67105200	4.00001400

N	9.53247300	0.01552700	-2.57402200
S	6.27342900	0.01895100	1.70511100
H	6.39896300	1.32935000	1.99196200
C	8.65394800	3.13071700	0.53768900
C	10.02873400	-1.38405900	1.65524700
C	6.33980300	-3.16515000	-0.94298300
C	5.01331900	1.35159700	-2.13033200
C	5.87868600	2.24400200	-1.50205000
C	5.78924300	3.68115900	-1.57011900
C	6.80774500	4.18064800	-0.81639500
C	7.53212200	3.05372800	-0.28506900
N	6.94456300	1.89909500	-0.72100600
C	9.37264000	2.06139400	1.06793600
C	10.51963000	2.17013100	1.93362800
C	10.90631100	0.90211800	2.24460200
C	9.99796500	0.00627400	1.57424900
N	9.09159300	0.74074200	0.86443500
C	9.14751500	-2.27695900	1.04956300
C	9.19321500	-3.71140700	1.18039800
C	8.15659800	-4.21242700	0.45338800
C	7.46836400	-3.08883100	-0.13036300
N	8.09807600	-1.93614700	0.24413500
C	5.64888300	-2.09539800	-1.50853400
C	4.48274800	-2.20604300	-2.34474800
C	4.11247000	-0.93358800	-2.68586700
C	5.05477700	-0.03947600	-2.05740200
N	5.97125400	-0.77458600	-1.36171100
H	8.99983400	4.12649400	0.79677600
H	10.81452400	-1.81707800	2.26645100
H	5.95695400	-4.15873700	-1.15444300
H	4.21348700	1.78342000	-2.72426300
H	5.04092800	4.22621400	-2.13099600
H	7.05977100	5.21716500	-0.63368800
H	10.96208100	3.10346100	2.25663100
H	11.72895700	0.58832600	2.87390000
H	9.92908000	-4.25424200	1.75925800
H	7.87269700	-5.24780300	0.31681600
H	4.01490900	-3.13724700	-2.63726900
H	3.30240200	-0.62436700	-3.33452700

Sum of electronic and zero-point Energies= -4318.807230

Sum of electronic and thermal Energies= -4318.738932

Sum of electronic and thermal Enthalpies= -4318.737988

Sum of electronic and thermal Free Energies= -4318.941907

[(bpb)Ru(NO)(HS)@Ag$_{20}$] MSI

Ru	-6.75179600	-0.02887500	0.38273900
O	-8.79852700	-3.45424000	-0.58310800
O	-8.27042700	3.70185800	-0.43785100
O	-7.14645700	0.02328700	2.28080100
N	-8.21835100	-1.23527400	-0.20153800
N	-8.00661900	1.40497600	-0.19174700
N	-5.83031300	-1.94498200	0.71414000
N	-5.54426700	1.71734800	0.75580000
C	-9.32370600	-0.53252600	-0.69946200

C	-9.21080500	0.89191400	-0.69143800
C	-10.27073600	1.66874700	-1.17444200
C	-11.42389200	1.05541800	-1.65480900
C	-11.53382700	-0.33373200	-1.66340600
C	-10.49219900	-1.12667700	-1.19132400
C	-8.02093100	-2.57303800	-0.21038800
C	-6.65990200	-2.94150700	0.32909000
C	-6.28875300	-4.27536500	0.42380200
C	-5.03328100	-4.59612100	0.93216900
C	-4.18868800	-3.56750400	1.33283700
C	-4.62710300	-2.25293600	1.20726900
C	-7.62470600	2.69923600	-0.12192900
C	-6.22522800	2.84022700	0.42779500
C	-5.66820900	4.10190500	0.58274200
C	-4.37641300	4.21653000	1.08811100
C	-3.67964700	3.05972000	1.41629800
C	-4.30105400	1.82704200	1.23421400
N	-6.96060100	0.08204600	3.40863400
S	-5.83052500	-0.12888700	-1.77446900
H	-10.16911700	2.74616600	-1.16246800
H	-12.23988600	1.67021300	-2.02353300
H	-12.43601200	-0.80796800	-2.03879700
H	-10.56184400	-2.20666000	-1.19316200
H	-6.99671200	-5.02886600	0.09797700
H	-4.72165700	-5.63231600	1.01664700
H	-3.19875000	-3.76087300	1.73298800
H	-3.99623700	-1.42276700	1.50659200
H	-6.26528700	4.96294500	0.30490100
H	-3.92131300	5.19297000	1.22111300
H	-2.66479100	3.09261200	1.79873000
H	-3.78453700	0.90305500	1.46990300
H	-5.87975400	1.17856600	-2.09394000
Ag	-3.20490800	-0.20734700	-1.53366300
Ag	-1.10313200	1.56311700	-0.80511700
Ag	-0.54909600	-0.81829300	-2.36036400
Ag	-1.33564000	-1.06069100	0.40377800
Ag	1.33157800	-1.78114000	-0.35999800
Ag	1.59438800	0.97505100	-1.60388400
Ag	0.71607700	0.73099000	1.28535200
Ag	0.98288500	3.28903400	0.00780600
Ag	2.12701200	-1.42948900	-3.09120800
Ag	0.46013100	-1.91262400	2.39920500
Ag	3.56293900	0.06940800	0.50650700
Ag	2.91418300	2.40047800	2.06258200
Ag	3.76683200	2.65302000	-0.74085500
Ag	2.65878800	-0.14882200	3.22827700
Ag	4.32282500	0.34244800	-2.25818700
Ag	3.24474300	-2.54979100	1.64538200
Ag	4.07002700	-2.31367800	-1.03805800
Ag	3.13774700	4.91304500	0.81938500
Ag	2.36593600	-2.72927600	4.30996000
Ag	4.82056300	-2.00370400	-3.72720800

Sum of electronic and zero-point Energies= -4598.990796

Sum of electronic and thermal Energies= -4598.921553
Sum of electronic and thermal Enthalpies= -4598.920608
Sum of electronic and thermal Free Energies= -4599.121011

[(Pc)Ru(NO)(HS)@Ag_{20}] MSI

Ru	-6.22007700	0.00235700	0.41719000
O	-7.11267100	-0.00611400	2.29982900
Ag	-2.76443300	-0.44691900	-1.65710700
Ag	-0.55487100	1.35026600	-1.56741800
Ag	-0.36148000	-1.76219700	-1.53447200
Ag	-0.81231500	-0.30834300	0.94227600
Ag	1.66345400	-1.58177700	0.66549500
Ag	1.87779600	0.03477400	-1.92296800
Ag	1.39948400	1.47635200	0.63894500
Ag	1.70488600	2.98673100	-1.80385200
Ag	2.22758600	-3.01925500	-1.87406300
Ag	1.13843400	-0.10925800	3.04052300
Ag	4.01456200	0.12481000	0.20742500
Ag	3.83114900	3.00995600	0.24689000
Ag	4.17933000	1.62606700	-2.32125500
Ag	3.49838200	1.56466900	2.64191300
Ag	4.34661200	-1.23378900	-2.36925600
Ag	3.74394700	-1.38720100	2.64755100
Ag	4.21380700	-2.78793300	0.24825200
Ag	4.08611200	4.44502100	-2.16743400
Ag	3.13163500	0.05128200	5.00045100
Ag	4.73071500	-4.08284200	-2.25255500
N	-8.30569700	0.02484600	2.48719300
S	-5.19516900	0.02190000	-1.90111000
H	-5.03319100	1.34964900	-2.05863000
C	-10.58807900	2.93613700	-1.38509800
C	-10.87591900	4.29715500	-1.46578100
C	-9.94659800	5.25901300	-1.04297100
C	-8.70682800	4.88367100	-0.52942500
C	-8.41692900	3.52458600	-0.44956300
C	-7.24811900	2.78967900	0.02433300
N	-6.15362600	3.36618200	0.50057900
C	-5.08634500	2.72491200	0.95755800
C	-3.89757500	3.39074700	1.47263000
C	-3.57240400	4.73557100	1.63187800
C	-2.33603300	5.05102800	2.19122100
C	-1.44763500	4.04169300	2.59122600
C	-1.77313900	2.69475200	2.43631700
C	-3.00177300	2.37426500	1.86153400
C	-3.66477300	1.10744800	1.55746700
N	-4.89565400	1.37291700	1.04119400
N	-3.12698400	-0.09885700	1.77031300
C	-3.74471000	-1.26801900	1.55863000
C	-3.17194400	-2.57368200	1.88216800
C	-1.98794100	-2.97069500	2.50351500
C	-1.75451700	-4.33544900	2.66738800
C	-2.68768600	-5.28699200	2.22913000
C	-3.88103900	-4.89476000	1.62738800
C	-4.11650400	-3.53193700	1.46165000

C	-5.24766300	-2.79278900	0.91852800
N	-4.97748900	-1.45502400	1.01581600
N	-6.33967700	-3.36446800	0.42907500
C	-7.39089800	-2.71813000	-0.05506900
C	-8.58807300	-3.37805200	-0.56736600
C	-8.94285900	-4.71773500	-0.69765500
C	-10.19213000	-5.01398900	-1.23907500
C	-11.06768300	-3.99375800	-1.63907000
C	-10.71509000	-2.65206200	-1.50739400
C	-9.46666900	-2.35375300	-0.96927300
C	-8.78758200	-1.09005700	-0.69143100
N	-7.56276600	-1.36679100	-0.16031300
N	-9.31676400	0.10077300	-0.94102700
C	-8.73148100	1.25451300	-0.64707800
N	-7.49074900	1.45299100	-0.11691800
C	-9.34975000	2.55888400	-0.87394500
H	-11.30635700	2.18934700	-1.71044200
H	-11.83441700	4.62092100	-1.86144400
H	-10.20027800	6.31269900	-1.11731500
H	-7.98630100	5.62660000	-0.20000700
H	-4.26925100	5.51290200	1.33280300
H	-2.05796400	6.09202500	2.32941000
H	-0.49295700	4.31358600	3.03370800
H	-1.08075000	1.91772700	2.75178200
H	-1.26235000	-2.23997900	2.85523200
H	-0.83922700	-4.66742300	3.14980300
H	-2.48004200	-6.34332100	2.37416600
H	-4.61444700	-5.62665600	1.30216800
H	-8.26411400	-5.50631900	-0.38627000
H	-10.49533000	-6.05084200	-1.35374500
H	-12.03509300	-4.25639700	-2.05767200
H	-11.39167200	-1.86032900	-1.81545900

Sum of electronic and zero-point Energies= -5202.711317
Sum of electronic and thermal Energies= -5202.633087
Sum of electronic and thermal Enthalpies= -5202.632143
Sum of electronic and thermal Free Energies= -5202.850958

[(Porph)Ru(NO)(HS)@Ag$_{20}$] MSI

Ru	6.93801100	-0.01228300	0.23319700
O	8.23570800	0.02548300	1.70856600
C	6.37765600	3.36338300	0.52219500
C	9.76697100	0.61199300	-1.59126300
C	7.43199300	-3.39000200	-0.16170100
C	4.15526400	-0.63968700	2.12645500
C	4.46717700	0.70094200	1.89906500
C	3.71212600	1.82699900	2.39102400
C	4.32734100	2.95097700	1.92845200
C	5.46729400	2.52074000	1.15626100
N	5.52577400	1.15338600	1.16454500
C	7.50669000	2.97968800	-0.20170200
C	8.44947400	3.87483600	-0.82650200
C	9.40671500	3.10102600	-1.40908100
C	9.05281500	1.72642200	-1.15170300
N	7.90087200	1.69201400	-0.41956500

C	9.43924500	-0.72951800	-1.39560400
C	10.18357600	-1.85516200	-1.90642700
C	9.52249700	-2.97942500	-1.51523800
C	8.37321400	-2.54964400	-0.75623300
N	8.35585400	-1.18743700	-0.70425800
C	6.32476100	-3.00309000	0.58987200
C	5.38682100	-3.89985300	1.21980200
C	4.47424600	-3.12780000	1.87286700
C	4.84327600	-1.75248800	1.64116000
N	5.96665400	-1.71154000	0.86721900
Ag	3.20367900	-0.21617900	-1.59366900
Ag	0.55604800	-0.84575600	-2.37482600
Ag	1.13031100	1.57492500	-0.84113200
Ag	1.40574000	-1.06734700	0.42993600
Ag	-0.65172400	0.74240200	1.26145800
Ag	-1.56486500	0.96746700	-1.63331200
Ag	-1.26554500	-1.77710700	-0.34605600
Ag	-2.11214300	-1.46195300	-3.08294700
Ag	-0.94903600	3.29901300	-0.03762600
Ag	-0.40047800	-1.89100000	2.42104700
Ag	-3.49275500	0.07538400	0.50545300
Ag	-4.01002900	-2.32826900	-1.00753600
Ag	-4.29343900	0.32052400	-2.25349100
Ag	-3.16998200	-2.53661900	1.67225300
Ag	-3.72930100	2.64967000	-0.76373500
Ag	-2.57650000	-0.11163300	3.23304400
Ag	-2.83902800	2.42498500	2.04215900
Ag	-4.81746000	-2.04429300	-3.67987200
Ag	-2.29713500	-2.68494500	4.34359500
Ag	-3.09942000	4.92427600	0.77783100
N	9.30413500	0.12698900	2.11241500
S	5.76698400	-0.02127000	-1.79414400
H	6.19579500	4.43037800	0.61036700
H	10.66832800	0.81081500	-2.16343900
H	7.58249200	-4.45769600	-0.29083200
H	3.28166200	-0.83820400	2.74203000
H	2.82384500	1.75294500	3.00588000
H	4.04911800	3.98375100	2.09619600
H	8.38023100	4.95491100	-0.81008400
H	10.27728900	3.42026700	-1.96747900
H	11.09193100	-1.78137100	-2.49051600
H	9.78167300	-4.01162600	-1.71298300
H	5.43659200	-4.98009900	1.17042500
H	3.62215900	-3.44675200	2.46008200
H	5.72490400	1.30709100	-2.01614200

Sum of electronic and zero-point Energies= -4524.803216
Sum of electronic and thermal Energies= -4524.736638
Sum of electronic and thermal Enthalpies= -4524.735694
Sum of electronic and thermal Free Energies= -4524.926808
